# HDAC1/2-Dependent P0 Expression Maintains Paranodal and Nodal Integrity Independently of Myelin Stability through Interactions with Neurofascins

**DOI:** 10.1371/journal.pbio.1002258

**Published:** 2015-09-25

**Authors:** Valérie Brügger, Stefanie Engler, Jorge A. Pereira, Sophie Ruff, Michael Horn, Hans Welzl, Emmanuelle Münger, Adrien Vaquié, Páris N. M. Sidiropoulos, Boris Egger, Peter Yotovski, Luis Filgueira, Christian Somandin, Tessa C. Lühmann, Maurizio D’Antonio, Teppei Yamaguchi, Patrick Matthias, Ueli Suter, Claire Jacob

**Affiliations:** 1 Department of Biology, University of Fribourg, Fribourg, Switzerland; 2 Institute of Molecular Health Sciences, Department of Biology, ETH Zurich, Zurich, Switzerland; 3 Department of Anatomy, University of Zurich, Zurich, Switzerland; 4 Anatomy, Department of Medicine, University of Fribourg, Fribourg, Switzerland; 5 Department of Materials, Laboratory for Biologically Oriented Materials, ETH Zurich, Zurich, Switzerland; 6 Division of Genetics and Cell Biology, San Raffaele Scientific Institute, DIBIT, Milano, Italy; 7 FMI for Biomedical Research, Novartis Research Foundation, Basel, Switzerland; Stanford University School of Medicine, UNITED STATES

## Abstract

The pathogenesis of peripheral neuropathies in adults is linked to maintenance mechanisms that are not well understood. Here, we elucidate a novel critical maintenance mechanism for Schwann cell (SC)–axon interaction. Using mouse genetics, ablation of the transcriptional regulators histone deacetylases 1 and 2 (HDAC1/2) in adult SCs severely affected paranodal and nodal integrity and led to demyelination/remyelination. Expression levels of the HDAC1/2 target gene myelin protein zero (P0) were reduced by half, accompanied by altered localization and stability of neurofascin (NFasc)155, NFasc186, and loss of Caspr and septate-like junctions. We identify P0 as a novel binding partner of NFasc155 and NFasc186, both in vivo and by in vitro adhesion assay. Furthermore, we demonstrate that HDAC1/2-dependent P0 expression is crucial for the maintenance of paranodal/nodal integrity and axonal function through interaction of P0 with neurofascins. In addition, we show that the latter mechanism is impaired by some P0 mutations that lead to late onset Charcot-Marie-Tooth disease.

## Abstract 2

The well-studied Schwann cell protein P0 is revealed to have an unsuspected function critical for the stability of paranodes and nodes in adult nerves. This function is specifically impaired by P0 mutations that lead to late-onset forms of Charcot-Marie-Tooth disease.

## Author Summary

Peripheral nerves consist mainly of axons and Schwann cells, which form myelin sheaths around axons. Peripheral neuropathies primarily affect axons, their myelin, or both. Etiologies are multiple: they can be inherited, autoimmune, infectious, metabolic (e.g., diabetes), or be due to tumors or toxic agents. However, the pathogenesis mechanisms of these disorders are not well understood. Here, we elucidate a novel critical mechanism in peripheral nerves for the stability of two adjacent structures of major importance for axonal function, the paranodes and nodes of Ranvier. We find that disruption of these structures causes a form of peripheral neuropathy. Ablation of the transcriptional regulators histone deacetylases (HDAC)1 and 2 in adult Schwann cells results in motor and sensory dysfunction, disruption of paranodal/nodal integrity, and loss of myelin. Expression of the HDAC1/2 target gene myelin protein zero (P0) was reduced by half, leading to altered localization of paranodal and nodal neurofascins, loss of paranodal Caspr, and impairment of axon–Schwann cell interaction in paranodal/nodal regions. We demonstrate that P0, the most abundant protein of peripheral compact myelin, extends to paranodes/nodes to maintain their stability by binding neurofascins. P0–neurofascins binding is affected by P0 mutations responsible for late onset forms of the inherited peripheral neuropathy Charcot-Marie-Tooth disease, identifying a pathogenesis mechanism of these disorders.

## Introduction

Myelinated axons are organized in distinct molecular domains, the axon initial segment, the internodes, juxtaparanodes, paranodes, and the node of Ranvier. This organization ensures proper clustering of ion channels, which is critical for the induction and fast propagation of electric signals along axons. In the peripheral nervous system (PNS), formation of these specialized domains requires the tight association of axons with Schwann cells (SCs), the myelinating glia of the PNS, except for the axon initial segment that forms independently of glial cells.

Disruption of these domains causes or aggravates many human disorders of the nervous system, including multiple sclerosis, motor and sensory neuropathies, Guillain-Barré syndrome, and most likely cognitive disorders [1–6]. Thus, extensive research has been carried out to determine the identity and functions of the molecular components of these domains [7–9]. However, our knowledge is incomplete and more work is required to fully understand the mechanisms that control their formation and preservation.

In this study, we elucidate the functions of histone deacetylases (HDACs) 1 and 2 and their target gene *myelin protein zero* (*P0*) in the maintenance of PNS integrity. By deacetylating histones at specific loci, HDACs locally modify the architecture of chromatin to control the transcription of their target genes [10,11]. HDACs can also deacetylate transcription factors and thereby modulate their activity [12,13]. These enzymes are thus very powerful transcriptional regulators. We have previously shown that the two class I enzymes HDAC1 and HDAC2 are critical for the specification of peripheral glia [14] and for postnatal development of SCs [15]. HDAC1 and HDAC2 are highly homologous nuclear enzymes that have important functions in myelination and survival [16]. Although they can have primary functions, they usually efficiently compensate for the loss of each other [14–17]. We showed in previous studies that HDAC2 and, to a lesser extent, HDAC1, interact with the major transcription factor of SC differentiation Sox10 to activate Sox10 target genes, including Sox10 itself and the other major transcription factor regulating myelination Krox20, as well as P0 [14,15]. In addition, we found that HDAC1 maintains survival of early postnatal SCs by preventing precocious activation of beta-catenin [15].

We show here that HDAC1/2 are not necessary to maintain Sox10 and Krox20 expression, nor SC survival in the adult PNS. Instead, HDAC1/2 are critical for the maintenance of paranodes and nodes of Ranvier integrity through their target gene *P0*. P0, the most abundant protein of PNS compact myelin, maintains the cohesion between two adjacent myelin wraps by homophilic adhesion properties [18]. Unexpectedly, we identify a novel function of P0 that is not linked to myelin compaction. Indeed, we demonstrate that P0 belongs to both paranodal and nodal adhesion complexes, where it is essential for the maintenance of these domains in the adult PNS.

Many mutations in the *P0* gene lead to peripheral neuropathies classified as Charcot-Marie-Tooth disease (CMT), with either early onset during childhood or late onset in adults [19]. In early-onset CMTs, demyelination and/or dysmyelination are prominent, whereas late-onset CMTs are usually characterized by altered axon–SC interaction and mild demyelination [19]. We show here that at least three P0 mutants that lead to late-onset CMT display homophilic adhesion properties comparable to wild type P0, but are unable to interact with components of the paranodal and nodal complexes, resulting in disruption of these structures, while myelin stability is maintained.

## Results

### HDAC1/2 Are Required in SCs for the Maintenance of Motor and Sensory Functions in Adult Mice

**Fig 1:**
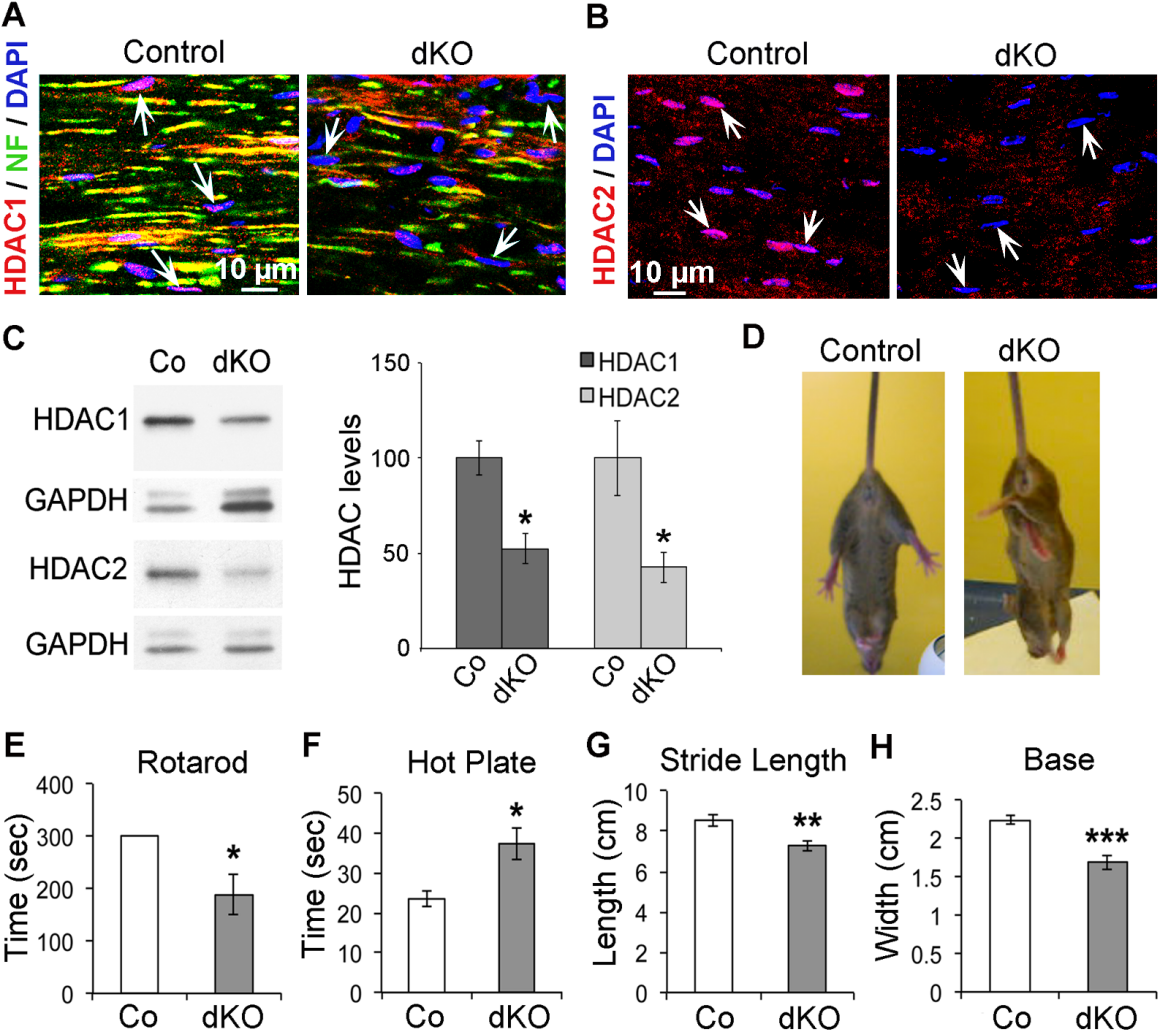
HDAC1/2 depletion in adult Schwann cells leads to motor and sensory dysfunction in peripheral nerves. Immunofluorescence of HDAC1 (A, red) and neurofilament (NF, green) or HDAC2 (B, red) in sciatic nerve cryosections of control and dKO mice at 8 wk post-tamoxifen showing efficient loss of HDAC1 and HDAC2 in dKO sciatic nerves. Nuclei are labeled in blue with DAPI. Arrows show elongated nuclei (presumably SC nuclei) expressing HDAC1 (A, pink) or HDAC2 (B, pink) in control, but are devoid of HDAC1 (A, blue) or HDAC2 (B, blue) in dKO sciatic nerves. Images of dKO sciatic nerves where HDAC1/2 were ablated in 85% of SCs are shown. (C) Western blot of HDAC1 and HDAC2 and quantification normalized to GAPDH (loading control) in sciatic nerves of adult HDAC1/2 dKO mice compared to control littermates (Co), showing protein loss at 7 d post-tamoxifen in dKO sciatic nerve lysates. (D) Representative photograph of tail-suspension test at 6 wk post-tamoxifen showing abnormal hind limb crossing in dKO mice, while control littermates show normal extension reflex. (E) Rotarod test showing impaired motor performances and (F) hot plate test identifying reduced sensitivity to heat of dKO mice compared to control littermates at 8 wk post-tamoxifen. Gait analysis showing affected stride length (G) and base (H) in dKO mice compared to control littermates (average of ten steps per mouse). Three to six animals per group were used for each experiment. *P*-values (two-tailed unpaired Student's *t* test): * = *p* < 0.05, ** = *p* < 0.01, *** = *p* < 0.001, error bars = standard error of the mean (SEM).

To determine whether HDAC1 and/or HDAC2 are required for the maintenance of peripheral nerves, we ablated HDAC1 and/or HDAC2 specifically in adult SCs. Mice expressing a tamoxifen-inducible Cre recombinase under the control of the *P0* promoter (P0CreERT2) [20] were crossed with animals carrying floxed *Hdac1* and/or floxed *Hdac2* alleles (H1fl/fl and/or H2fl/fl) [21]. Single heterozygous (P0CreERT2-H1fl/wt or-H2fl/wt, called thereafter H1HTZ and H2HTZ), single homozygous (P0CreERT2-H1fl/fl or-H2fl/fl, called thereafter H1KO and H2KO), and double homozygous (P0CreERT2-H1fl/fl-H2fl/fl, called thereafter dKO) mutants were generated. At three months of age, HDAC1 and/or HDAC2 were ablated in SCs by tamoxifen injections. At 8 wk post-tamoxifen, elongated nuclei (presumably SC nuclei) had lost HDAC1 (Fig 1A) and/or HDAC2 (Fig 1B) in dKO nerves. Consistent with previous studies using the same P0CreERT2 mouse line [22], recombination efficiency was variable between animals: HDAC1/2 were ablated in 45% to 85% of SCs. To determine the onset of protein loss, we carried out western blot analyses on sciatic nerve lysates of control and dKO mice. HDAC1/2 protein levels were reduced already 7 d post-tamoxifen injection (Fig 1C). While single HDAC mutants did not display obvious defects, dKO mice developed a strong maintenance phenotype of hindlimb weakness (Fig 1D), which started at 6 wk post-tamoxifen and remained stable until at least 12 months. We carried out functional analyses at 8 wk post-tamoxifen and found that both motor and sensory functions were affected in dKO mice, as evidenced by reduced performance on the rotarod (Fig 1E) and decreased sensitivity to heat in the hot plate test (Fig 1F). In addition, gait analysis tests identified significantly reduced stride length (Fig 1G) and base (Fig 1H) in dKO mice compared to controls. These data demonstrate that HDAC1/2 are essential in SCs to maintain adult PNS function.

### Loss of HDAC1/2 in Adult SCs Leads to Demyelination/Remyelination and Decreased P0 Expression

**Fig 2:**
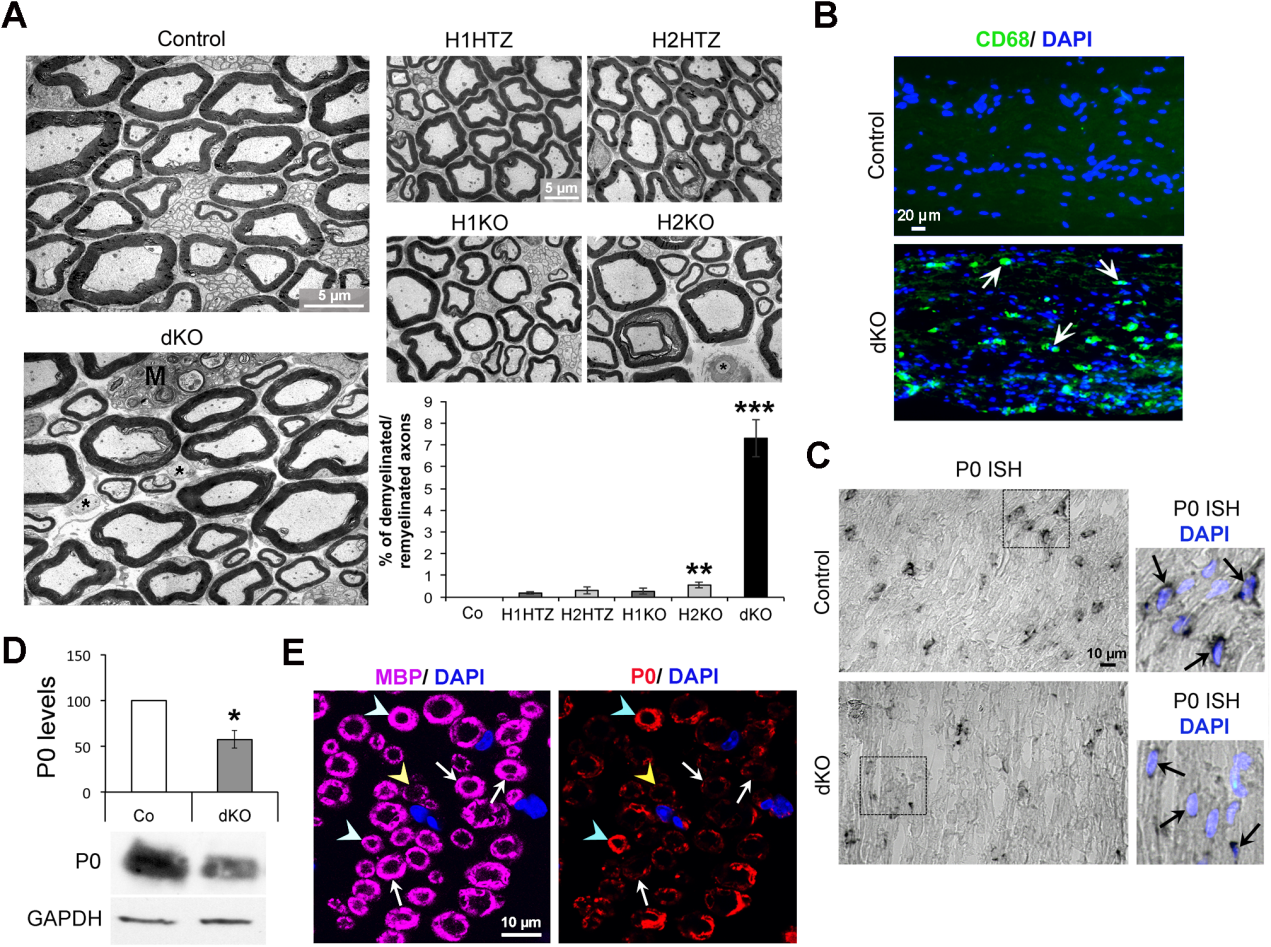
Demyelination/remyelination and decreased P0 expression in dKO mice. (A) Electron micrographs of ultrathin cross sections of control, dKO, H1HTZ, H2HTZ, H1KO, and H2KO sciatic nerves, at 8 wk post-tamoxifen, and percentage of demyelinated/remyelinated axons (3 animals per group, at least 700 axons counted per mouse), identifying a demyelination/remyelination phenotype in dKO sciatic nerves. Asterisks indicate demyelinated axons and “M” macrophages. (B) CD68 (green) immunofluorescence in longitudinal cryosections of control and dKO sciatic nerves labeled with DAPI (blue = nuclei) showing increased presence of macrophages in dKO sciatic nerves, consistent with the demyelination phenotype. Arrows indicate macrophages. (C) In situ hybridization (ISH) of P0 on longitudinal cryosections of control and dKO sciatic nerves identifying a reduction of P0 at the transcript level at 5 wk post-tamoxifen, before the onset of demyelination and when macrophages are not present in the nerve. Pictures on the right are magnifications of black boxes depicted on the left. Black arrows show SC nuclei. (D) Western blot of P0 and quantification normalized to GAPDH (loading control) in sciatic nerve lysates of control and dKO mice at 8 wk post-tamoxifen (3 mice per group), showing reduced P0 protein levels in dKO sciatic nerves. (E) Confocal images of MBP (magenta) and P0 (red) coimmunofluorescence in paraffin cross sections of dKO mice at 8 wk post-tamoxifen, showing reduced P0 levels in most myelin rings, while MBP levels remain high. Nuclei are labeled in blue by DAPI. A single optical section is shown. White arrows indicate MBP positive/P0 negative myelin rings, the yellow arrowhead shows an MBP/P0 double negative SC, and blue arrowheads MBP/P0 double positive myelin rings. Three animals per group were used for each experiment. *P*-values (two-tailed unpaired (A) or paired (D) Student's *t* test): * = *p* < 0.05, ** = *p* < 0.01, *** = *p* < 0.001, error bars = SEM.

To identify the defects responsible for the loss of function due to ablation of HDAC1/2 in adult SCs, we carried out morphological analyses. We found that 7% of axons were either demyelinated or remyelinated (thin myelin) in dKO sciatic nerves (Fig 2A), while axon diameters were unaffected (S1A Fig). Indeed, we measured a slight increase in the percentage of axons of middle range caliber, but no significant difference in axon diameter average (controls: 4.81 ± 0.54 μm and dKO: 4.23 ± 0.37 μm, *p*-value = 0.21). We also examined potential defects in single heterozygous and homozygous mutants, but found no defect in H1HTZ, H2HTZ or H1KO, and only 0.5% of demyelinated or remyelinated axons in H2KO (Fig 2A). This indicates that in adult SCs, such as in other systems [23,24], HDAC1 and HDAC2 can efficiently compensate for the loss of each other. Indeed, HDAC2 levels were up-regulated in H1KO nerves compared to controls (S1B Fig), but HDAC1 levels were not up-regulated in H2KO nerves (S1C Fig). This suggests that efficient compensation of HDAC1 function may require the up-regulation of HDAC2, while HDAC1 compensates HDAC2 function without up-regulation. However, the small but significant percentage of demyelinated/remyelinated axons in H2KO nerves (Fig 2A) shows that compensation by HDAC1 is incomplete. Consistent with myelin breakdown, we detected increased presence of macrophages in dKO nerves by morphological analyses (Fig 2A, labeled M) and immunofluorescence using the macrophage marker CD68 (Fig 2B, arrows). We previously found that HDAC1/2 interact with the transcription factor Sox10 to induce the expression of P0 in SC precursors and postnatal SCs [14,15]. In postnatal SCs, we also showed that the Sox10/HDAC complex controls the expression of Sox10 itself and Krox20 [15]. Consistently, P0 expression was reduced at the transcript levels at 5 wk post-tamoxifen (Fig 2C) before the influx of macrophages in the nerves and when no demyelinated/remyelinated axon was found (S2A Fig). P0 was also reduced at the protein level at 5 wk (S2B Fig) and 8 wk post-tamoxifen (Fig 2D) in dKO nerves, but remained unaffected in H1HTZ, H2HTZ, H1KO, and was slightly decreased in H2KO nerves at 8 wk post-tamoxifen (S2C Fig). However, myelin basic protein (MBP) levels were not significantly affected at 5 wk (S2D Fig) or 8 wk post-tamoxifen (S3A Fig). The transcription factors Sox10 and Krox20 were not affected either (S3B and S3C Fig), and myelin-associated glycoprotein (MAG) was slightly increased (S3D Fig). By immunofluorescence, we detected many cells that had lost P0 but expressed high levels of myelin basic protein (MBP) (Fig 2E, white arrows) in dKO nerves at 8 wk post-tamoxifen, indicating that loss of P0 precedes the loss of MBP in demyelinated cells. A few SCs had only residual levels of both P0 and MBP (Fig 2E, yellow arrowhead), and a few others expressed high levels of both P0 and MBP (Fig 2E, blue arrowheads). In early postnatal SCs, we previously showed that HDAC1/2 limit the levels of active beta-catenin (ABC) to prevent apoptosis. However, we did not find differences in ABC levels (S3E Fig) or apoptosis (S4A Fig) between dKO and control nerves. Proliferation of SCs, analyzed by BrdU incorporation at 5 wk post-tamoxifen, before the influx of macrophages, was also not affected (S4B Fig). Taken together, these data show that HDAC1/2 are necessary in adult SCs for the maintenance of high P0 expression and for optimal myelination but not for SC survival.

### Loss of HDAC1/2 in Adult SCs Results in Disruption of Paranodal and Nodal Structures

**Fig 3:**
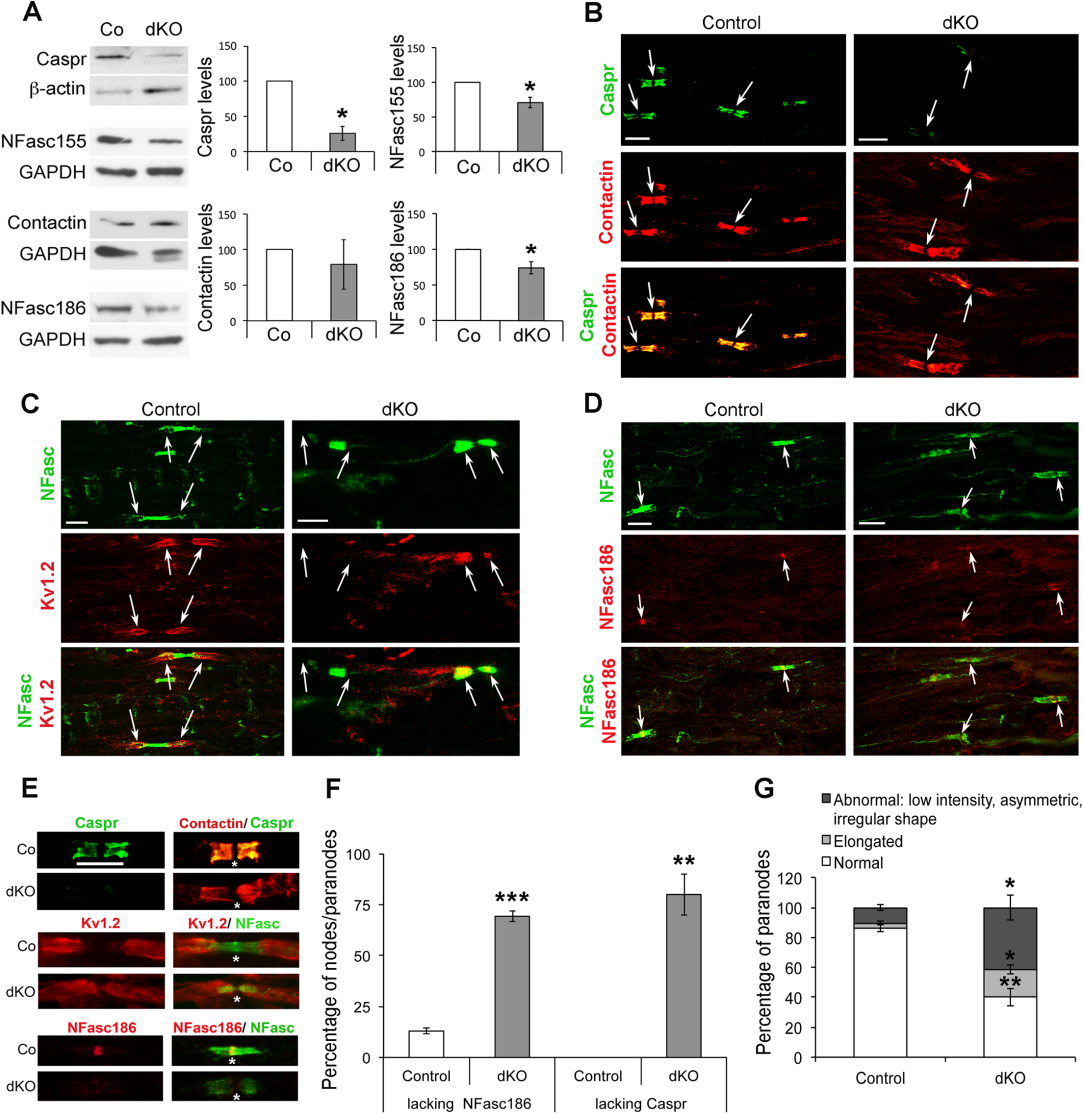
Loss of neurofascins and Caspr in dKO mice. (A) Western blots of Caspr, NFasc155, Contactin, NFasc186, and quantification normalized to the loading control β-actin or GAPDH on sciatic nerve lysates of dKO mice compared to control littermates (set at 100%) at 8 wk post-tamoxifen (three mice per group) identifying reduced levels of Caspr and neurofascins. Coimmunofluorescence of (B) Contactin (red) with Caspr (green) or (C) total neurofascins (green) with Kv1.2 (red) or (D) total neurofascins (green) with NFasc186 (red), and (E) magnifications of representative single nodal/paranodal structures, on longitudinal cryosections of control and dKO sciatic nerves at 8 wk post-tamoxifen, showing loss of Caspr in paranodes and of NFasc186 in nodes and mislocalization or loss of Kv1.2 in dKO sciatic nerves. Overlays appear yellow. Representative images of three control and three dKO mice are shown. Arrows show the position of nodes in B, D, and of Kv1.2 signal (in juxtaparanodes in control, and in paranodes or absence of signal in dKO) in C. In E, asterisks mark the position (lateral dimension) of the node of Ranvier. Scale bars = 5 μm. (F–G) Percentage of nodes lacking NFasc186 and paranodes lacking Caspr (F), and of normal, elongated, and abnormal (low intensity, asymmetric, irregular shape) paranodes based on NFasc staining in paranodes (G) in control and dKO nerves at 8 wk post-tamoxifen, demonstrating paranodal/nodal defects in dKO sciatic nerves. Three animals per group were used for each experiment. In F and G, 50 to 100 nodes/paranodes counted per animal, 180 to 230 counted per genotype. *P*-values (two-tailed paired (A) or unpaired (F,G) Student's *t* test): * = *p* < 0.05, ** = *p* < 0.01, *** = *p* < 0.001, error bars = SEM.

To detect potential additional defects in HDAC1/2 mutants, we analyzed the molecular composition and the morphology of nodes of Ranvier and paranodes on cryosections of mutant sciatic nerves at 8 wk post-tamoxifen. Strikingly, the paranodal axonal protein Caspr was nearly lost in dKO sciatic nerves (Fig 3A and 3B), while Caspr was unaffected in H1HTZ, H2HTZ, H1KO, and slightly affected in H2KO paranodes (S5A and S5C Fig). To detect the paranodal SC-localized neurofascin (NFasc)155 and the nodal axonal NFasc186, we used a pan-neurofascin antibody. In dKO nerves, NFasc155 protein levels were decreased (Fig 3A), and in the majority of paranodes, NFasc155 localization appeared widened and its distribution diffused (Fig 3C–3E). Protein levels of Contactin, a third known component of the paranodal complex, localized on the axonal side, were not significantly affected compared to control nerves (Fig 3A), and Contactin was present in dKO paranodes (Fig 3B and 3E). Consistent with the loss of Caspr [25], K_v_1.2 voltage-gated K^+^ channels were no longer restricted to juxtaparanodes, but had moved to paranodes in dKO nerves (Fig 3C and 3E) and protein levels were significantly reduced (Fig 3C and S6A Fig). In addition, NFasc186 protein levels were also decreased (Fig 3A), and immunoreactivity was not detectable or weak in the node of Ranvier of dKO nerves (Fig 3C–3E), while NFasc186 was unaffected in H1HTZ, H2HTZ, H1KO, and slightly affected in H2KO paranodes (S5B and S5C Fig). Magnifications of a representative paranodal/nodal structure for each staining are shown in Fig 3E. Quantification revealed that 70 ± 3% of nodes lack NFasc186 and 80 ± 10% of paranodes lack Caspr in dKO sciatic nerves (Fig 3F). We used NFasc staining in paranodes to quantify the frequency of normal, elongated, and abnormal (low intensity, asymmetric, irregular shape) structures. In dKO nerves, the percentage of elongated and abnormal paranodes was significantly increased compared to control nerves (Fig 3G).

Interestingly, Nav1.6 voltage-gated Na^+^ channels (S6A Fig), Gliomedin (S6A Fig) and Ankyrin G (S6B Fig) protein levels were increased in dKO nerves. Clustering of Ankyrin G (S6B Fig), Nav1.6 (S6C Fig), and NrCam (S6D Fig) in the node, and of beta-Dystroglycan (S6E Fig), phosphorylated ezrin/radixin/moesin (pERM, S6F Fig), and Gliomedin (S6G Fig) in SC microvilli were maintained in dKO nerves.

**Fig 4:**
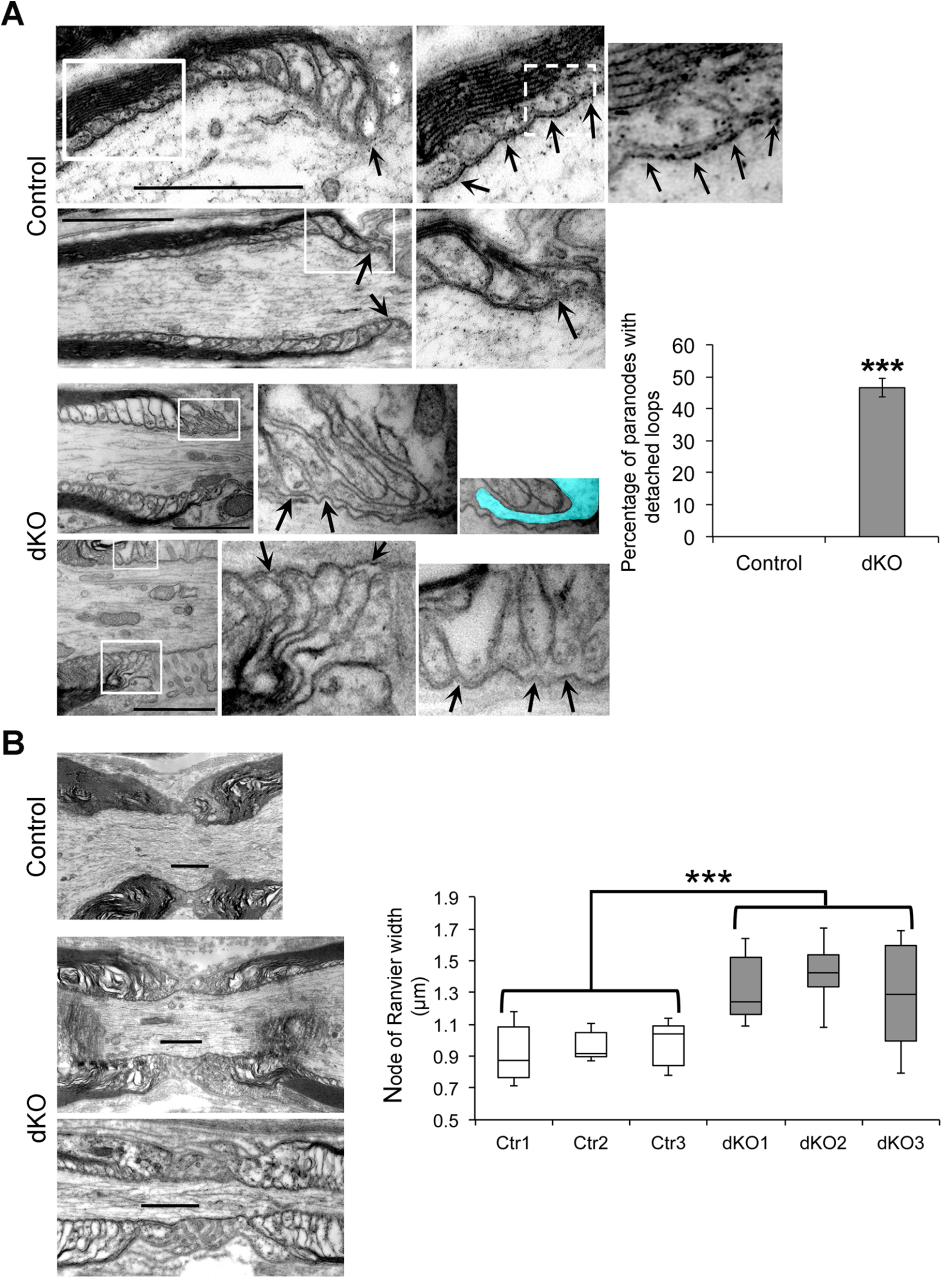
Detached paranodal loops and wider nodes in dKO. Electron micrographs of ultrathin longitudinal control and dKO sciatic nerve sections at 8 wk post-tamoxifen showing in (A) paranodal loops attached to the axolemma and septate-like junctions (arrows) in control nerves, and detached paranodal loops devoid of septate-like junctions (arrows) in dKO nerves. In some dKO nodes, microvilli (highlighted in blue, image on the right) invaded the space between paranodal loops and the axolemma. Images on the right are magnifications of white boxes depicted on the left images. The graph representing the percentage of paranodes with detached loops in control and dKO demonstrates frequent occurrence of these defects in dKO sciatic nerves. Three animals per genotype were used, 11 to 38 paranodes were counted per animal, and 56 to 72 were counted per genotype. In (B), electron micrographs represent nodes of control (Ctr in the graph) and dKO nerves, and the quantification of nodal widths in the graph shows significant widening of the nodal region in dKO sciatic nerves. Three animals per genotype were used for quantification. The average width of 7 to 17 nodes of Ranvier was calculated per animal (*n* = 3), a total of 32 to 42 nodes were measured per genotype. Scale bars = 1 μm. In (A), error bar = SEM. In (B), the graph is a box plot where the lower box (Median − Quartile 1) and the upper box (Quartile 3 − Median) are separated by the Median value and flanked by top and bottom Whiskers. *P*-values (unpaired two-tailed Student's *t* test): *** = *p* < 0.001, *n* = 3.

Septate-like junctions in paranodes are formed by the paranodal complex NFasc155/Caspr/Contactin. Each of these three proteins is necessary for the formation of septate-like junctions [25–31] and thus for the fence function of paranodes to prevent voltage-gated K^+^ channels to invade paranodes. To analyze the morphology of paranodes in dKO nerves, we carried out electron microscopy on longitudinal ultrathin sections of control and dKO sciatic nerves at 8 wk post-tamoxifen. While we could detect septate-like junctions in the paranodes of control nerves, we could rarely detect them in dKO nerves (Fig 4A), and in some cases microvilli were invading the space between paranodal loops and the axolemma (Fig 4A, region highlighted in blue). We quantified the percentage of paranodes with detached loops, which is a direct consequence of loss of septate-like junctions. We found 46 ± 3% of paranodes with detached loops in dKO nerves, whereas we did not detect paranodes with detached loops in control nerves (Fig 4A). In addition, nodes of Ranvier were significantly wider in dKO (1.35 ± 0.03 μm) compared to control (0.95 ± 0.01 μm) nerves (Fig 4B). This is consistent with the consequences of loss of Caspr in the PNS [25,26].

At 5 wk post-tamoxifen, we did not detect demyelinated axons in dKO nerves (S2A Fig). However, we found a significant increase in the percentage of nodes lacking NFasc186, and of paranodes with low levels or asymmetric distributions of Caspr in dKO compared to control nerves (S7A and S7B Fig). In addition, there was an increased percentage of abnormal (low intensity, asymmetric, irregular shape) paranodes (S7C Fig), as shown by NFasc staining in paranodes (S7A Fig). This indicates that the disruption of paranodes/nodes of Ranvier precedes demyelination, and is therefore independent of the demyelination phenotype identified in dKO nerves.

### Exogenous P0 Maintains Myelination, Caspr, and Neurofascins in dKO DRG Cultures

To identify the molecular mechanisms by which HDAC1/2 control the maintenance of paranodal and nodal integrity, and optimal myelination, we carried out in vitro myelination assays using dorsal root ganglion (DRG) explants of dKO embryos and control littermates. We cultured DRG explants until they reached a myelination plateau, and then added tamoxifen to induce ablation of HDAC1/2. For these experiments, we generated HDAC1/2 dKO using a tamoxifen-inducible Cre recombinase under control of the *PLP* promoter (PLPCreERT2) [20], because efficiency of in vitro recombination was high with PLPCreERT2 (S8A Fig). These mutants are called *plp* **-**dKO thereafter.

**Fig 5:**
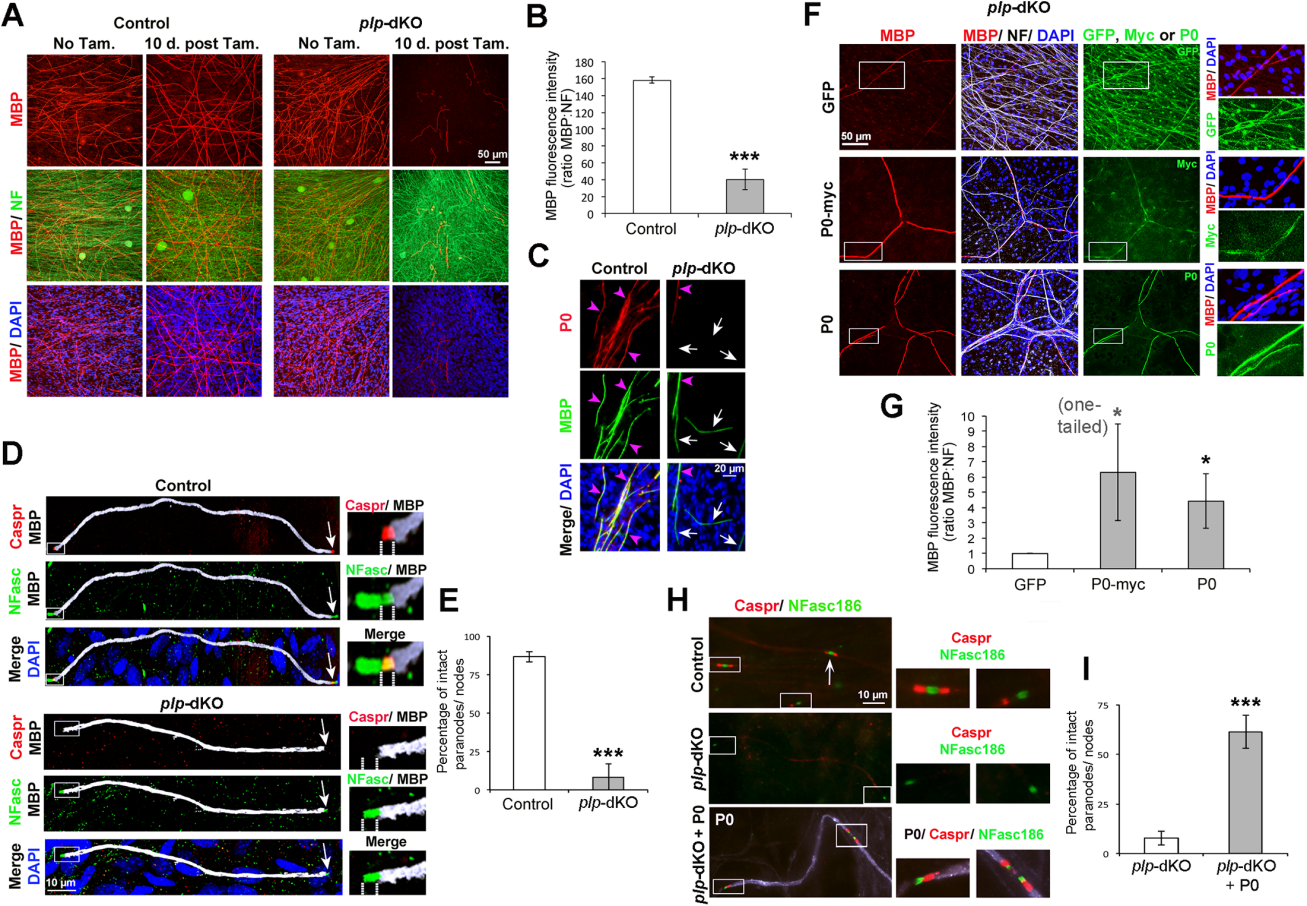
P0 rescues myelination, Caspr, and neurofascins in *plp*-dKO DRG cultures. Confocal coimmunofluorescence images of (A) MBP (red) and neurofilament (NF, green), or (C) P0 (red) and MBP (green), or (D) Caspr (red), total neurofascins (NFasc, green), and MBP (white), or (F) MBP (red), NF (white), and either GFP, P0, or Myc (green), or (H) Caspr (red) and NFasc186 (false-colored green) in myelinated control and *plp* **-**dKO DRG cultures with (A,C,D,F,H) or without (A) tamoxifen. Briefly, A–D demonstrate demyelination, loss of P0 and paranodal/nodal defects in *plp* **-**dKO DRG cultures, mimicking the in vivo phenotype of dKO sciatic nerves. In (F,H), *plp* **-**dKO DRG cultures were transduced with doxycycline-inducible lentiviruses expressing either GFP, P0, or P0-myc. (B,G) Quantification of MBP fluorescence intensity normalized to NF. (E,I) Percentage of intact nodes/paranodes expressing Caspr and high levels of NFasc. F–I show that exogenously delivered P0 significantly rescues demyelination and paranodal/ nodal defects of *plp* **-**dKO DRG cultures. In (C), white arrows indicate MBP positive/P0 negative fibers, and magenta arrowheads MBP/P0 double positive fibers. Merges MBP/P0 (C) or Caspr/NFasc (D) appear yellow. In (D), dashed lines delineate the paranodal region. In (H), control and *plp* **-**dKO were transduced with lentiviruses expressing GFP, and *plp* **-**dKO + P0 with lentiviruses expressing P0 (white). Z-series projections (A,C,F) and single optical sections (D,H) are shown. In (D,H), arrows indicate heminodes or full nodes. Images on the right are magnifications of white boxes depicted on the left images. At least three control and three *plp* **-**dKO embryos were used for quantification (average of three coverslips per embryos), representative images are shown. Nuclei are labeled in blue with DAPI. *P*-values (unpaired [B,E,I] or paired [G] two-tailed Student's *t* test, unless stated otherwise in the figure): * = *p* < 0.05, *** = *p* < 0.001, error bars = SEM.

Ten days after tamoxifen addition, *plp* **-**dKO DRG cultures had severely demyelinated, as shown by reduction of MBP fluorescence intensity (Fig 5A and 5B) and the presence of myelin debris (S8B Fig). Consistent with our in vivo data (Fig 2E), most of the remaining MBP-expressing fibers in *plp* **-**dKO DRG had lost P0 expression (Fig 5C). In addition, while Caspr and neurofascins were robustly present in myelinated fibers of control DRG cultures, Caspr was either not or rarely detected in the remaining MBP-expressing fibers of *plp* **-**dKO DRG cultures, and neurofascins were either not detectable or reduced compared to control cultures (Fig 5D and 5E). P0 transcription is directly activated by the complex Sox10/HDAC1/2 [14,15], and ablation of HDAC1/2 in adult SCs in vivo leads to a similar phenotype as for P0-null mice, i.e., motor and sensory loss of function, demyelination/remyelination, presence of macrophages, and loss of Caspr in sciatic nerves [32–34]. In addition, we found that all paranodes/nodes in adult P0 homozygous knockout (P0 KO) and 77 ± 6% of paranodes/nodes in adult P0 heterozygous mutant (P0 HTZ) sciatic nerves exhibited diffused and widened NFasc155 in paranodes and loss of NFasc186 in nodes of Ranvier (S9A–S9C Fig). Thus, we hypothesized that P0 is the main HDAC1/2-dependent gene that is essential for maintenance of paranodes/nodes integrity and of optimal myelination in adult SCs. To test this hypothesis, we generated doxycycline-inducible lentiviruses expressing either untagged P0, P0 myc-tagged at the intracellular C-terminus (P0-myc), or green fluorescent protein (GFP) as a control. We transduced SCs with these lentiviruses at the start of DRG cultures and induced P0 expression only at the myelinated stage, just before ablating HDAC1/2 in SCs. P0 myc-tagged at the intracellular C-terminus is inserted into the membrane and functions comparable to wild type P0 [35]. Indeed, both untagged P0 and P0-myc were able to prevent demyelination in *plp* **-**dKO DRG cultures (Fig 5F and 5G). Both P0 and P0-myc also rescued Caspr and increased neurofascins in paranodes/nodes of *plp* **-**dKO DRG cultures (Fig 5H and 5I, and S10 Fig). In summary, myelinating cultures using DRG explants of *plp* **-**dKO embryos allowed us to mimic the in vivo phenotype resulting from loss of HDAC1/2 in adult SCs. We therefore used this system for rescue experiments and demonstrated that P0 is responsible for HDAC1/2-dependent maintenance of paranodes and nodes of Ranvier integrity, and for optimal myelination in adult SCs.

### P0 Is a Component of Paranodal and Nodal Complexes in the Adult PNS

The rescue of Caspr and neurofascins by P0 in MBP-expressing fibers was intriguing and prompted us to seek the molecular mechanism of P0 function in the maintenance of paranodal and nodal structures.

**Fig 6:**
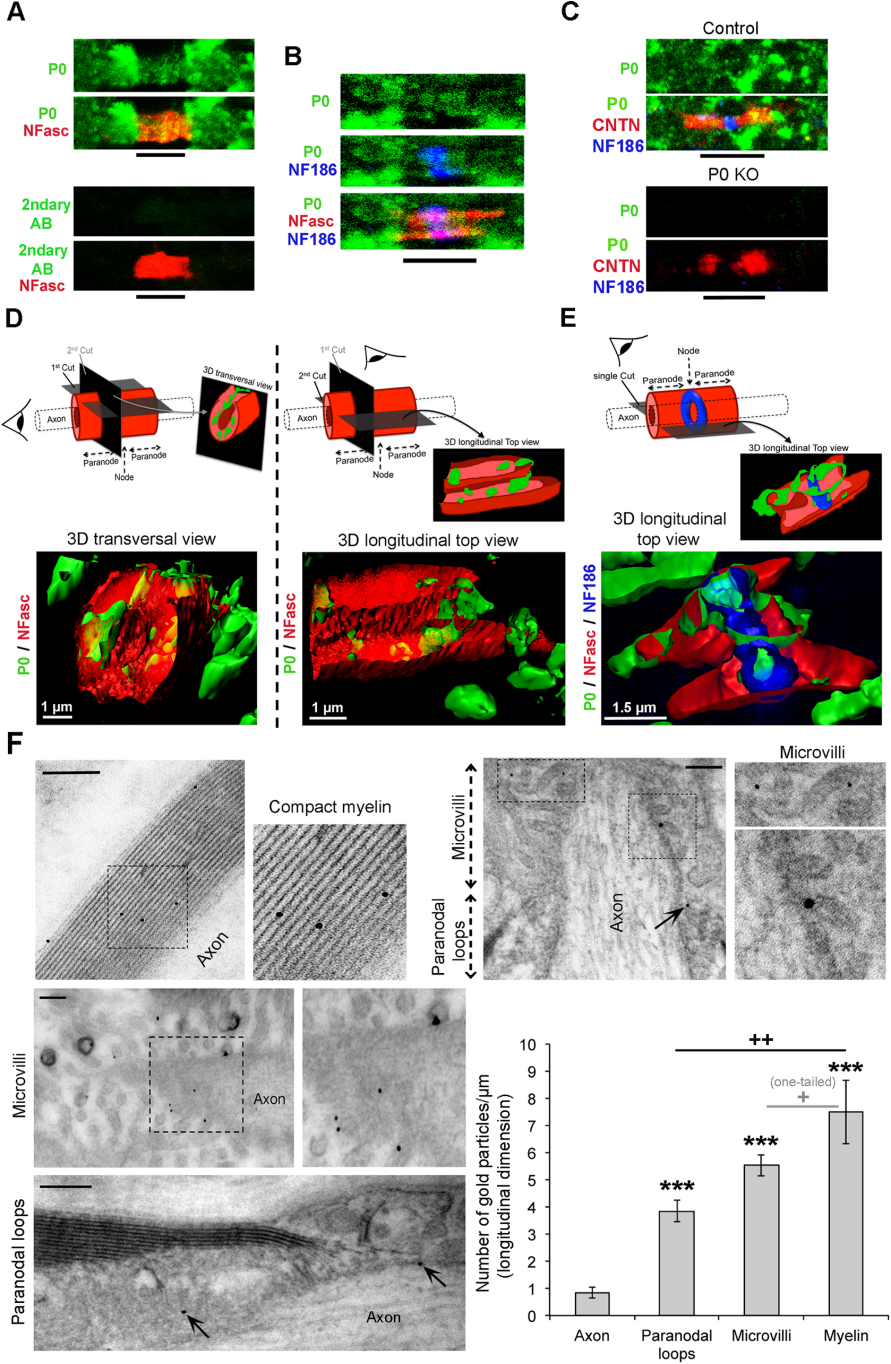
P0 is present in internodes, paranodes, and nodes of Ranvier of the PNS. Confocal images (z-series projections) of (A) P0 (green) or Alexa Fluor 488-AffiniPure Goat Anti-chicken IgG (2ndary AB) as negative control and total neurofascins (NFasc, red) coimmunofluorescence or (B) P0 (green), total NFasc (red) and NFasc186 (blue), or (C) P0 (green), Contactin (CNTN, red), and NFasc186 (blue) coimmunofluorescence on longitudinal cryosections (5-μm thick) of wild type (A,B) or P0 KO and control littermate (C) adult (3 months old in A,B; 10 months old in C) mouse sciatic nerves. These stainings show robust P0 signal in internodal, paranodal, and nodal regions of wild type and control sciatic nerves, while no P0 signal is detected in P0KO sciatic nerves, demonstrating the specificity of the P0 antibody used. 3-D reconstruction by Imaris software of (D) P0 (green) and total NFasc (red) coimmunofluorescence and (E) P0 (green), total NFasc (red) and NFasc186 (blue) coimmunofluorescence in wild type nerves, showing colocalization of P0 with neurofascins. Interestingly, there is no visible gap between NFasc155 and NFasc186 signals. A 3-D transversal view (D) and 3-D longitudinal views (D,E) are shown together with schematic representations of optical cuts through the structure and the angle of view (schematic eye) above each 3-D image. Surfaces of P0 and total NFasc (D) or of P0, total NFasc and NFasc186 (E) are represented in combination with volumes (raw staining signal) of total NFasc (D, red punctuated staining) or NFasc186 (E, blue punctuated staining). Six paranodes/nodes have been analyzed by 3-D reconstruction and representative images are shown. (F) P0 detection by immunoelectron microscopy using ultrasmall gold particles plus silver enhancement carried out on whole adult mouse sciatic nerves before embedding and sectioning. Immunogold density is low using this protocol, but specific to compact myelin, paranodes (arrows) and microvilli. Scale bars = 200 nm. Images on the right are magnifications of dashed-line boxes depicted on left images. Sciatic nerves of three wild-type mice were analyzed and representative pictures are shown. Quantification of the number of gold particles per μm (longitudinal length) found in paranodal loops, microvilli, or myelin shows significant P0 staining in these structures compared to associated axons (background staining), with most abundant signal in myelin sheaths, followed by microvilli and then paranodal loops. Thirteen paranodes, 15 microvilli, 6 myelin sheaths, and 24 associated axons were quantified. No gold particles were found labeling endoneurial fibroblasts or nonmyelinating SCs associated with small caliber axons. *P*-values (unpaired two-tailed Student's *t* test, unless stated otherwise in the figure): **+** = *p* < 0.05, **++** = *p* < 0.01, *** = *p* < 0.001 (asterisks show significance compared to axons), error bars = SEM.

Unexpectedly, we found that P0 localization was not restricted to internodes such as MBP, but extended further to the region between two internodes in DRG myelinating cultures (S11A Fig), suggesting the presence of P0 in paranodes and nodes of Ranvier. We confirmed the presence of P0 in paranodes and nodes of Ranvier in vivo by coimmunofluorescence of P0 with total NFasc, NFasc186, and Contactin on cryosections of adult mouse sciatic nerves (Fig 6A–6C) and adult human peripheral nerves (S11B and S11C Fig) and 3-D reconstruction of immunofluorescence signals in mouse sciatic nerves (Fig 6D and 6E). In addition, by immunoelectron microscopy on adult mouse sciatic nerves, we detected P0 specifically localized in compact myelin as expected, and also in paranodal loops and microvilli (Fig 6F).

**Fig 7:**
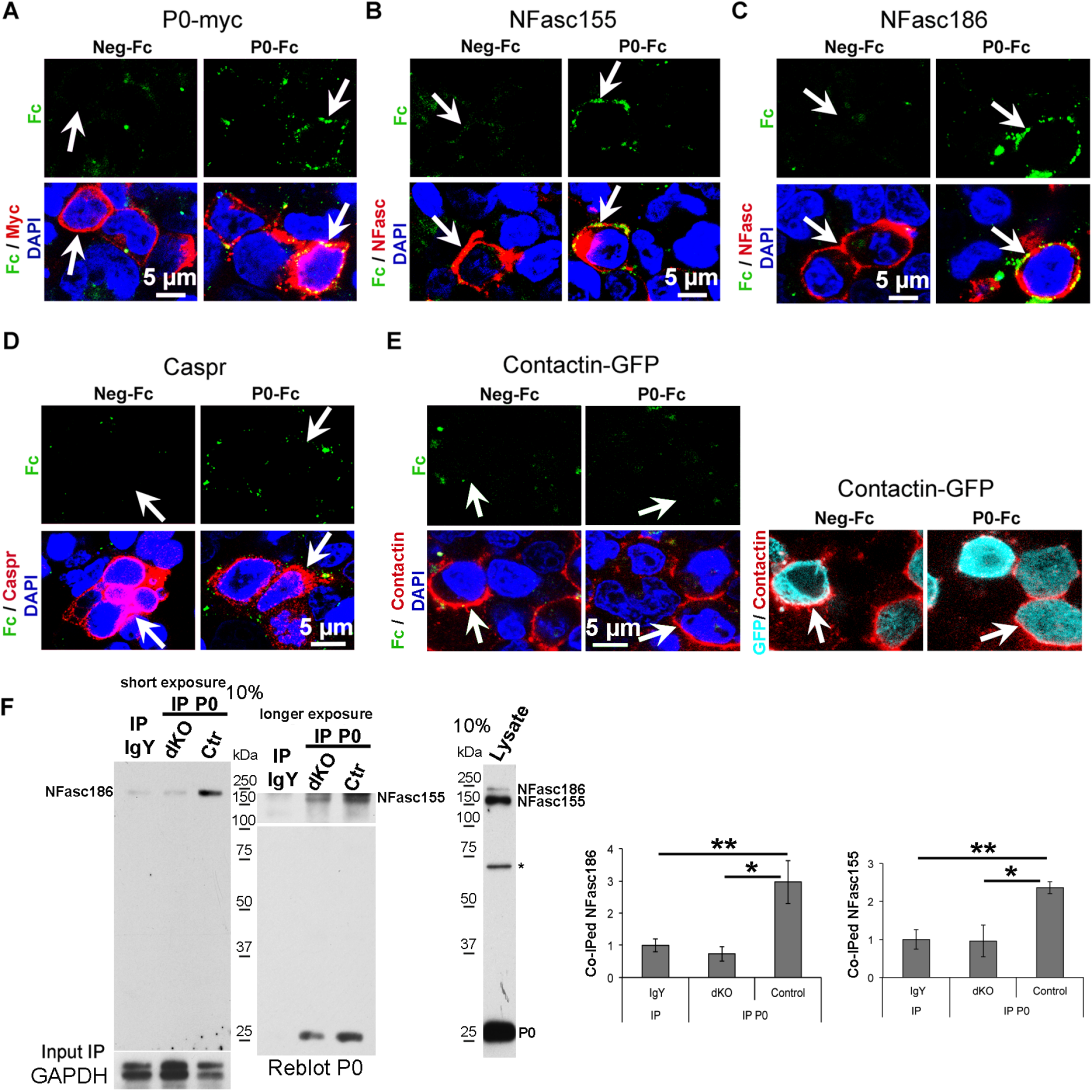
P0 interacts with neurofascins. (A–E) Adhesion assay in HEK293T cells showing homophilic adhesion of P0 extracellular domain, as well as binding to the extracellular domain of NFasc155 and NFasc186. Confocal images of P0-Fc or control-Fc (Neg-Fc) particles (green, or false-colored green for Contactin-GFP) and Myc (A, red), neurofascins (B and C, red), Caspr (D, red), or Contactin (E, red) coimmunofluorescence and GFP fluorescence (E, false-colored turquoise; right side of the panel) in HEK293T cells expressing P0-Myc (A), NFasc155 (B), NFasc186 (C), Caspr (D) or Contactin-GFP (E), indicated by arrows. Overlays appear yellow. Nuclei are labeled in blue with DAPI. Single optical sections are shown. Each experiment was done at least three times and representative pictures are shown. (F) Immunoprecipitation (IP) IgY (negative control with chicken IgY) and IP P0 (with chicken anti-P0 antibody) from adult dKO and control littermate sciatic nerve lysates and detection with pan-NFasc antibody, and western blot for total NFasc on a 10% acrylamide SDS-Page gel, and reblot of immunoprecipitations (IPs) and western blot with P0 antibody. GAPDH western blot on lysates used for IgY and P0 IPs shows input. The band marked by an asterisk is most likely nonspecific. The graphs showing the quantification of coimmunoprecipitated (IPed) NFasc186 or NFasc155 with P0 (normalized to GAPDH input and calculated as fold change compared to IgY IP which was set to 1) in sciatic nerves of three dKO and three control littermate mice at 8 wk post-tamoxifen indicate that binding of P0 to neurofascins occurs in vivo in sciatic nerves of wild type mice, but is largely lost in dKO mice. Both sciatic nerves of each animal were pooled and split into two equal volumes for each IP IgY and P0. *P*-values (unpaired two-tailed Student's *t* test): * = *p* < 0.05, ** = *p* < 0.01, error bars = SEM.

NFasc155 is necessary for the maintenance of paranodes and is the only known transmembrane component of the paranodal complex that is expressed on the SC side [27,36]. Because P0 was necessary for the maintenance of Caspr and neurofascins, and was localized in paranodes and microvilli, we hypothesized that P0 is another binding partner within the paranodal complex and of the nodal complex. To test this hypothesis, we generated Fc particles fused to the extracellular domain of P0 (P0-Fc, S12A and S12B Fig) and control Fc particles (neg-Fc, S12A Fig) and carried out adhesion assays. The extracellular domain of P0 forms homotetramers connecting two myelin wraps [18], and as expected, P0-Fc bound to HEK293T cells expressing P0-myc at their cell surface, whereas neg-Fc particles did not (Fig 7A and S13A Fig). We found that P0-Fc also decorated the surface of HEK293T cells expressing NFasc155 or NFasc186 at their plasma membrane, whereas neg-Fc particles did not (Fig 7B and 7C and S13B and S13C Fig). P0-Fc did not bind to HEK293T cells expressing Caspr or Contactin (Fig 7D and 7E and S13D and S13E Fig). These data indicate that the extracellular domain of P0 is capable of interacting with the extracellular domain of NFasc155 and of NFasc186. We then tested by coimmunoprecipitation whether these interactions also occur in vivo. Indeed, NFasc155 and NFasc186 coimmunoprecipitated with P0 (Fig 7F) in adult control sciatic nerves, showing that P0 interacts with these two neurofascins in vivo, whereas coimmunoprecipitated NFasc levels were strongly reduced in dKO sciatic nerves (Fig 7F). These data demonstrate that P0 belongs to the paranodal and nodal complexes in the adult PNS.

### Three P0 Mutants Causing Late Onset CMT Maintain Homophilic Adhesion Properties, but Their Binding to Neurofascins Is Impaired

**Fig 8:**
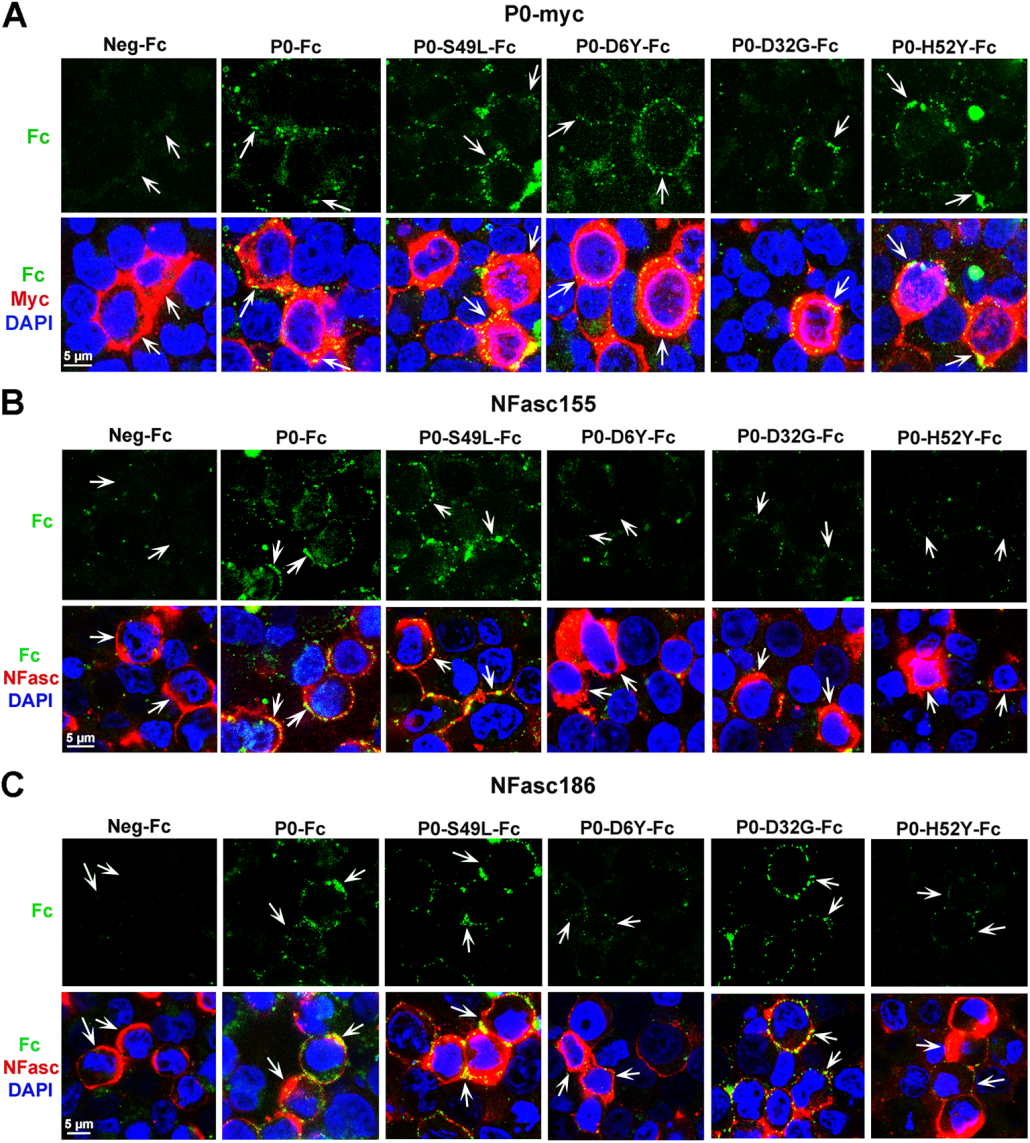
The four P0 mutations D6Y, D32G, H52Y and S49L result in three different binding profiles to neurofascins: preserved (S49L), impaired binding to NFasc155 (D32G), impaired binding to both NFasc (D6Y and H52Y), while binding to P0 is maintained for all mutants. Adhesion assay in HEK293T cells. Confocal images of P0-Fc, P0-D6Y-Fc, P0-D32G-Fc, P0-H52Y-Fc, P0-S49L-Fc or control-Fc (Neg-Fc) particles (green) and neurofascins or Myc (red) coimmunofluorescence in HEK293T cells expressing P0-myc (A), NFasc155 (B) or NFasc186 (C), indicated by arrows. Overlays appear yellow. Nuclei are labeled in blue with DAPI. Single optical sections are shown. At least three independent experiments were analyzed for each panel and representative pictures are shown.

To investigate the functional relevance of P0 interaction with neurofascins further, we aimed at disrupting the P0/NFasc interaction without altering the homophilic adhesion properties of P0. If functionally relevant, P0/NFasc interaction is likely to be affected by some of the P0 mutations that are responsible for subtypes of the human disease, CMT. We generated Fc particles fused to the extracellular domain of P0 carrying either the D6Y, or D32G, or H52Y mutations (S12B Fig), which are known to cause late onset CMT with mild demyelination [19,37–39]. We also generated the S49L P0-Fc mutant (S12B Fig) that causes either early or late onset CMT with severe demyelination, myelin decompaction and focally folded myelin [19,40–41]. While P0-D6Y-Fc, P0-D32G-Fc and P0-H52Y-Fc were able to bind to HEK293T cells expressing P0-myc (Fig 8A and S14A Fig), similar to wild type P0-Fc, they were unable to bind to NFasc155 (Fig 8B and S14B Fig). In addition, binding of P0-D6Y-Fc and P0-H52Y-Fc to NFasc186 was strongly reduced (Fig 8C and S14C Fig), while binding of P0-D32G-Fc to NFasc186 was maintained (Fig 8C and S14C Fig). P0-S49L-Fc was bound to P0-myc (Fig 8A and S14A Fig), but in contrast to the other P0 mutants, also showed binding to both neurofascins (Fig 8B and 8C and S14B and S14C Fig).

### P0/NFasc Interaction Is Critical for the Integrity of Paranodal/Nodal Structures, but Not for the Stability of Myelin

**Fig 9:**
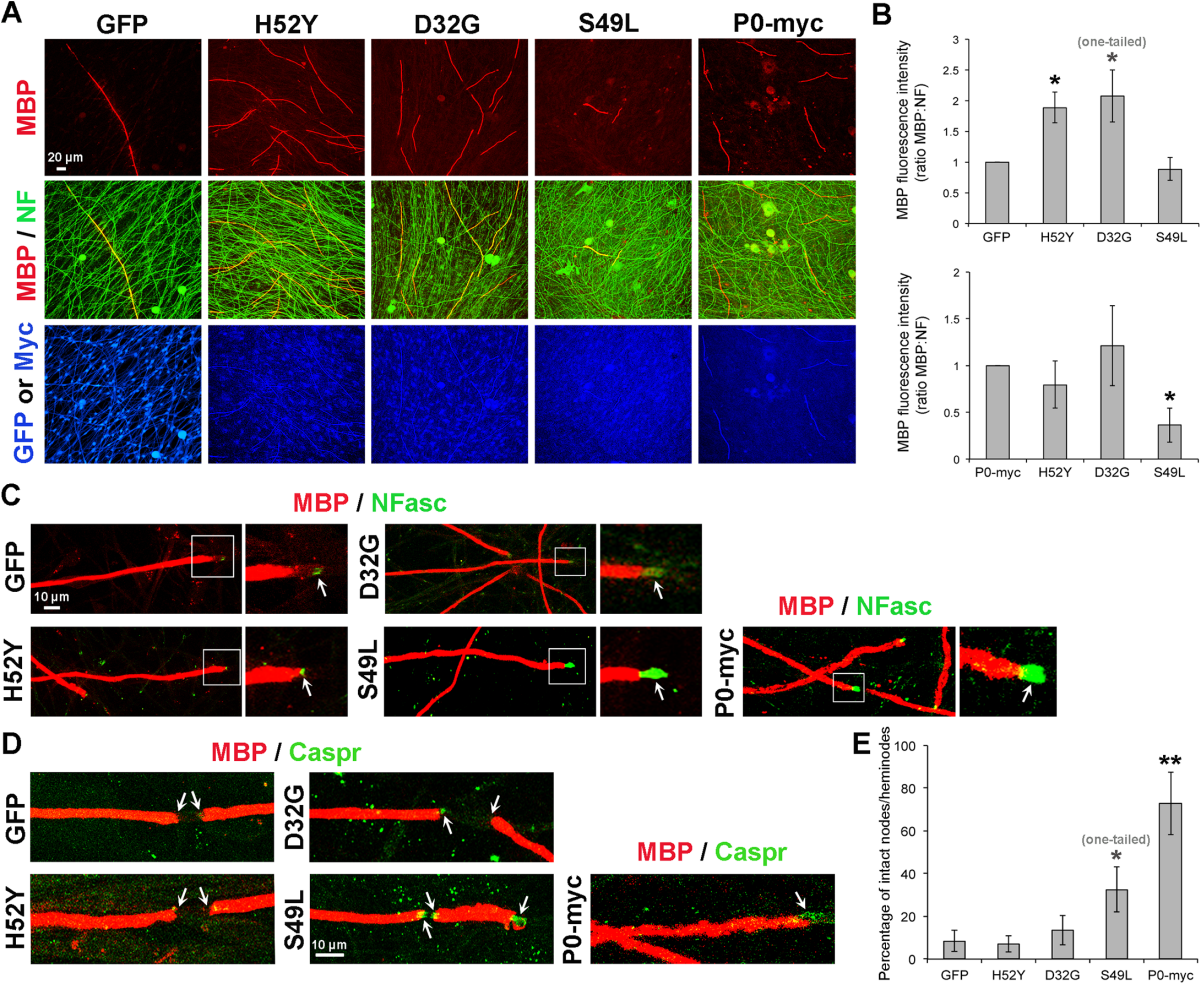
In contrast to the S49L P0 mutant, D32G and H52Y P0 mutants rescue myelination of HDAC1/2 *plp*-dKO DRG but not paranodal/nodal integrity. Coimmunofluorescence of MBP (red) and (A) neurofilament (NF, green), and Myc or GFP fluorescence (blue), or (C) neurofascins (NFasc, green), or (D) Caspr (green) in myelinated HDAC1/2 *plp*-dKO DRG cultures transduced with lentiviruses expressing either GFP, H52Y-myc, D32G-myc, S49L-myc or P0-myc, and treated with tamoxifen for 10 d after completion of myelination. A–B show that H52Y and D32G but not S49L P0 mutants are able to rescue myelination of *plp*-dKO DRG cultures, similarly to P0-myc, and C–D show that S49L, but not H52Y or D32G, P0 mutant is able to partially rescue paranodal/nodal defects of *plp*-dKO DRG cultures. In (C), pictures on the right are magnifications of the white boxes depicted on left images. Arrows indicate paranodes/nodes. In (B), quantification of MBP fluorescence intensity normalized to NF and compared to GFP or P0-myc (set to 1). DRG of six *plp*-dKO embryos were quantified (three *plp*-dKO embryos per graph, four coverslips per *plp*-dKO). In (C,D), DRG of three *plp*-dKO embryos were analyzed and representative pictures are shown. In (E), the graph represents the percentage of intact (Caspr-positive or high NFasc levels) nodes and heminodes. DRG of three *plp*-dKO embryos were quantified, four coverslips per *plp*-dKO, 80 to 300 nodes/heminodes counted per *plp*-dKO per virus. *P*-values (paired (B) and unpaired (E) two-tailed (unless stated otherwise in the figure) Student's *t* test): * = *p* < 0.05, ** = *p* < 0.01, error bars = SEM.

We showed that exogenous P0 and P0-myc were able to prevent demyelination and disruption of nodes and paranodes due to HDAC1/2 ablation in SCs of myelinated DRG cultures (Fig 5F–5I and S10 Fig). We then asked whether the loss of P0/NFasc interaction alters the ability of P0 to maintain the integrity of paranodal/nodal structures and the stability of myelin. To answer this question, we generated lentiviruses carrying myc-tagged H52Y, D32G, or S49L P0 mutants that exhibited three different NFasc binding profiles by adhesion assay (Fig 8): impaired binding to both NFasc for H52Y, impaired binding to NFasc155 for D32G, preserved binding to both NFasc for S49L. All generated mutants reached the plasma membrane of differentiated primary rat SCs (S15 Fig). By GFP fluorescence and Myc staining, we demonstrated comparable and highly efficient transduction of DRG cultures by all lentiviruses we generated (Fig 9A and S16 Fig). In HDAC1/2 *plp* **-**dKO cultures transduced at the start of the culture with lentiviruses expressing H52Y and D32G P0 mutants, MBP levels reflecting myelination were significantly higher compared to *plp* **-**dKO DRG cultures transduced with lentiviruses expressing GFP and were similar to *plp* **-**dKO DRG cultures transduced with lentiviruses expressing P0-myc (Fig 9A and 9B), indicating that H52Y and D32G P0 mutants were able, such as wild-type P0, to prevent demyelination due to the loss of HDAC1/2. However, H52Y was unable to rescue neurofascins and Caspr in paranodes and nodes of Ranvier (Fig 9C–9E), whereas D32G partly rescued neurofascins, but at low levels, and did not rescue Caspr (Fig 9C–9E). In *plp* **-**dKO DRG cultures transduced with lentiviruses expressing the S49L P0 mutant, MBP levels were not increased compared to *plp* **-**dKO cultures transduced with GFP-expressing lentiviruses and were lower compared to *plp* **-**dKO cultures transduced with lentiviruses expressing P0-myc (Fig 9A and 9B), indicating that S49L was unable to prevent demyelination due to loss of HDAC1/2. However, in the few remaining MBP-positive fibers, high neurofascin levels and Caspr were more frequently detected in nodes/heminodes compared to *plp* **-**dKO cultures transduced with GFP-expressing lentiviruses (Fig 9C–9E), but less frequently compared to *plp* **-**dKO cultures transduced with lentiviruses expressing P0-myc (*p*-value = 0.044, one-tailed unpaired Student *t* test). In parallel, we analyzed potential defects of myelination (MBP levels) and paranodes/nodes (NFasc and Caspr) in DRG cultures of control littermate embryos transduced with lentiviruses in the same conditions as *plp* **-**dKO DRG cultures, but no differences of MBP levels (S17A and S17B Fig) or in the percentage of intact nodes/heminodes (S17C–S17E Fig) were detected between GFP, H52Y, D32G, S49L, or P0-myc.

These data are consistent with the binding profiles of P0 mutants to neurofascins: preserved for S49L, impaired for H52Y and partially impaired for D32G. They are also consistent with the absence of demyelination (or mild demyelination) phenotype in late onset CMT caused by D32G and H52Y mutations, and with the strong demyelination phenotype caused by S49L P0 mutation in early or late onset CMT.

In addition to demonstrating the necessity of P0/NFasc interaction for the maintenance of paranodal/nodal integrity independent of myelin stability, these data identify the impairment of P0 binding to one or both neurofascins as a likely contributing pathogenesis mechanism of late onset CMT, caused by at least the three P0 mutants D6Y, D32G, and H52Y tested in this study. We thus demonstrate the functional relevance of P0 interaction with neurofascins and the critical dependence of the adult PNS upon this mechanism for maintenance of integrity.

## Discussion

We previously showed that HDAC1/2 direct neural crest cells into the glial lineage [14] and are necessary for SC survival and to induce the transcriptional program of myelination during development [15]. In contrast, we show here that HDAC1/2 are not required in adult SCs for expression of inducers of myelination or survival. However, we found that HDAC1/2 are essential in adult SCs to maintain the integrity of the PNS through their target gene *P0*. We demonstrate that P0 is a novel component of paranodal and nodal adhesion complexes and is critical to preserve the structure of these domains in adult peripheral nerves.

Ablation of HDAC1/2 in adult SCs resulted in partial motor and sensory loss of function with moderate demyelination/remyelination. Consistently, MBP was lost in only a few fibers. However, P0 was reduced in most fibers, suggesting that loss of P0 precedes the loss of MBP in these mutants. In addition, these data indicate that low levels of P0 are sufficient to maintain at least temporary stability of the myelin sheath in adult SCs. This is consistent with the phenotype observed in heterozygous P0 deficient mice and in a subset of patients suffering from late onset CMT due to P0 mutations [19,32]. In addition, we found widened and diffused localization and decreased levels of NFasc155 and severe loss of Caspr in paranodes of HDAC1/2 dKO nerves, while the presence of NFasc186 in nodes of Ranvier was strongly reduced. Consistent with the loss of Caspr [25,26], paranodal loops were detached from the axolemma, as a consequence of disrupted septate-like junctions. Kv1.2 K^+^ channels were mislocalized to paranodes, and nodes of Ranvier were wider in these mutants. Interestingly, Contactin levels were not significantly affected in dKO nerves. This contrasts with the reported developmental functions of Caspr [25] and NFasc155 [31], where deletion of Caspr or NFasc155 leads to loss of Contactin in paranodes. It thus appears that Caspr and NFasc155 are essential for initial clustering but not for maintenance of Contactin in paranodes of adult nerves.

Unexpectedly, we found a strong decrease of NFasc186 in nodes of HDAC1/2 dKO nerves, while Nav1.6 Na^+^ channels, Gliomedin, and Ankyrin G were maintained. During development, loss of NFasc186 leads to failure of Nav channels clustering at the node [31,42]. However, Amor and colleagues [43] show that absence of Gliomedin and NrCAM leads to loss of NFasc186, while Nav channels are maintained, as long as Ankyrin G is still present at the node. This is consistent with our present findings. In addition, the maintenance of Gliomedin in dKO nodes indicates that in the absence of NFasc186, other components of the node that interact with Gliomedin, such as NrCam [44] and/or ECM proteins [26], are able to stabilize Gliomedin localization.

We previously found in the SC lineage that HDAC1/2 have the peculiar ability to activate transcription of specific target genes when bound to the transcription factor Sox10 [14,15]. Indeed, Sox10 requires HDAC1/2 as cofactors to activate the *P0* promoter in SCs and P0 expression is barely detectable in developing HDAC1/2-null SCs [14,15]. P0-deficient mice show demyelination/remyelination [32], decreased Caspr in paranodes, mislocalization of voltage-gated K^+^ channels [34], and alteration of neurofascins localization (S9 Fig), similar to the ablation of HDAC1/2 in adult SCs. Thus, we hypothesized that demyelination and disruption of paranodes and nodes of Ranvier in adult HDAC1/2 dKO mice are due to the loss of P0. Myelinated DRG cultures from HDAC1/2 *plp*-dKO embryos, treated with tamoxifen to induce the ablation of HDAC1/2, mimicked the loss of P0, Caspr, and neurofascins in MBP-expressing fibers and the demyelination phenotype of adult HDAC1/2 dKO mice. P0 exogenously delivered by inducible lentiviruses just prior to HDAC1/2 ablation maintained paranodal/nodal localization of Caspr and neurofascins, and prevented demyelination in these cultures. This demonstrates that HDAC1/2-dependent P0 expression in SCs is essential to maintain the integrity of paranodes and nodes of Ranvier, and optimal myelination in the adult PNS.

Caspr and Contactin expressed at the axolemma and NFasc155 expressed at the paranodal glial loops form the paranodal complex and are essential for the formation of septate-like junctions [25–31]. It was thought that MBP and P0 are exclusively localized to compact myelin in internodes; however, our analyses revealed that while MBP localization is indeed restricted to internodes, P0 is also localized in paranodes and nodes of Ranvier. We show here that the maintenance of paranodes and nodes of Ranvier structure requires the interaction of P0 with NFasc155 and NFasc186. The decrease of NFasc155 and NFasc186 total protein levels (Fig 3A) is significant in dKO compared to control nerves, but moderate, and the levels of NFasc155 transcripts are unchanged in dKO compared to control sciatic nerves (S18 Fig). Thus, by interacting with NFasc, P0 may stabilize the localization of NFasc in paranodes and nodes of Ranvier. Although different pools of P0 may interact with either NFasc155 or NFasc186, our findings open the possibility of interaction between paranodal and nodal complexes through P0 as a physical linker at the interface of these complexes. This hypothesis is supported by our 3-D reconstructions in which no gap was detected between axonal and glial neurofascins (Fig 6D and 6E).

Further strengthening the functional relevance of P0 interaction with neurofascins, we demonstrate that this interaction is impaired by the three P0 mutations D6Y, D32G, and H52Y [19,37–39], which are responsible for late onset forms of the human disease, CMT. Interestingly, homophilic adhesion among P0 proteins is not affected by these three mutations. Consistently, these P0 mutants prevent demyelination but are unable to maintain neurofascins and Caspr in paranodes and nodes of HDAC1/2 *plp*-dKO DRG myelinated cultures. In contrast, S49L P0 mutation that leads to either early or late onset CMT with strong demyelination, is able to bind both NFasc and to maintain high levels of NFasc and Caspr in HDAC1/2 *plp*-dKO DRG cultures, but it is unable to prevent demyelination. In addition to unraveling a new function of P0 in maintaining the integrity of paranodes/nodes of Ranvier independently of its function in maintaining myelin stability, these data identify a previously undescribed molecular mechanism that leads to pathological consequences in some disease-causing P0 mutants and suggest that other P0 mutants leading to late onset CMT may also act through the same or related pathogenesis mechanism. Consistent with our findings, detailed morphological analysis of a sural nerve biopsy from a patient harboring a D32G P0 mutation shows frequent abnormal structure in the paranodal region [38].

NFasc155 and NFasc186 are generated by alternative splicing of the same gene and differ by only a few amino acids: their extracellular part consists of six immunoglobulin (Ig)-like domains, four fibronectin type 3 (FNIII)-like domains (1st, 2nd, and 4th are common to both NFasc, while NFasc155 has the 3rd and NFasc186 the 5th FNIII-like domain), and in addition, NFasc186 possesses a mucin-like domain [45]. It is therefore not surprising that P0 binds both NFasc by adhesion assay. We have identified two amino acids (D6 and H52) in the Ig-like domain of P0 that are required for interaction of P0 with both NFasc and one amino acid (D32) required for binding of P0 to NFasc155, but not to NFasc186 (Fig 8B and 8C). This suggests that P0 can bind to NFasc155 and NFasc186 through common, but also distinct, domains. However, whether P0 binds to both axonal and glial NFasc in vivo through the same domains remains to be determined. It is conceivable that the folding and/or flexibility of NFasc186 or NFasc155 extracellular domain in vivo allows binding to P0 through the same domains. However, our coimmunoprecipitation data of NFasc with P0 in adult mouse sciatic nerves hint towards that binding of P0 to NFasc186 is stronger than to NFasc155, indicating that P0 may interact with both NFasc through at least partially distinct domains in vivo. In addition, such as P0, NFasc are subject to post-translational modifications including glycosylation of their extracellular domain, which can potentially modify their interaction with extracellular binding partners.

Among the three amino acids D6, D32, and H52 we found required for the binding of P0 to one or both NFasc, D6 is not predicted by the crystal structure of P0 extracellular domain [18] to be directly involved in P0 homophilic adhesion, but H52 is predicted to belong to the putative adhesion interface, and D32 to the head-to-head interface of P0 proteins [18]. However, according to our data obtained by in vitro myelination and/or adhesion assay, the three amino acids D6, D32, and H52 do not appear essential for the maintenance of P0 homophilic adhesion properties. This is consistent with the late onset phenotype and mild demyelination resulting from the mutation of these residues in humans [19]. Thus, despite the putative involvement of H52 and D32 in P0 homophilic adhesion, the mutation of D32 in G (Asp32 in Gly) or H52 in Y (His52 in Tyr) may not be sufficient to dramatically alter P0–P0 interaction.

In summary, our study shows essential functions of HDAC1/2-dependent P0 expression in the maintenance of paranodal and nodal structures and of optimal myelination in the adult PNS. We demonstrate that P0 is a critical component of the paranodal and nodal adhesion complexes (our findings are summarized in S19 Fig), and we identify a novel function of P0 that is independent of the maintenance of myelin stability. This critical function of P0 in the adult PNS is impaired by at least three P0 mutations that lead to late onset CMT, while myelination is mostly preserved, thus uncovering a novel pathogenesis mechanism of these P0 mutations.

## Materials and Methods

### Ethics Statement

Isoflurane was used for mouse anesthesia, and Pentobarbital was used for mouse euthanasia. Animal use was approved by the veterinary offices of the Cantons of Zürich and of Fribourg, Switzerland. Authorization numbers: 129/2011 and 2012_05_FR. For human samples, the body donors consented in the context of the body donation program of the University of Fribourg that tissues could be used for research purposes, according to the Swiss Ethics regulation.

### Generation of Inducible Conditional Knockout Mice

Mice homozygous for floxed *Hdac1* and/or floxed *Hdac2* alleles [21] were crossed with inducible P0CreERT2 or PLPCreERT2 mice, in which expression of the Cre recombinase (Cre) is controlled by the Schwann cell-specific *P0* promoter or the Schwann cell and oligodendrocyte-specific *PLP* promoter [20]. The Cre recombinase is produced as a fusion protein with the hormone-binding domain of the estrogen receptor, allowing Cre activity to be turned on by intraperitoneal injection of tamoxifen. To ablate HDAC1 and HDAC2 in adult SCs, three to four month-old adult mice received daily injections of 2 mg tamoxifen (Sigma) for five consecutive days. Time of analysis is calculated from the last day of tamoxifen injection. As control mice, we used Cre-negative littermates of HDAC1 and/or HDAC2 knockout mice. P0 knockout (heterozygous and homozygous) mice were previously described [46]. Genotypes were determined by PCR on genomic DNA.

### Behavior

Eight weeks post-tamoxifen injections, mice were placed five times on the rotarod apparatus to test balance and motor coordination (day 1). The duration of each trial was limited to 300 s, and trials were separated by a 30 min recovery period. Latency to fall from the rotating beam was recorded. On day 2, mice were individually placed on a hot plate (52°C), and the latency for the appearance of paw licking as a sign of unpleasant heat was recorded. On day 3, footprints were recorded to analyze gait abnormalities. Mice were first trained to swiftly walk through a narrow runway (1 m in length) to a goal box. After inking their hind paws with nontoxic paint, paw prints left on the white paper lining the floor were analyzed (three runs). Parameters were stride length (distance between consecutive prints of the same paw) and base (distance between hind paw prints). All behavioral tests were carried out with the same experimental animals (six dKO and six control littermate mice, three females and three males per group). Animals of control and dKO groups had similar body length and weight.

### Collection of Human Peripheral Nerves

Samples of human median, tibial, and sciatic nerves were collected from cadaveric fresh frozen upper and lower limbs of body donors. The limbs were frozen within 24 h after death. The excised nerves were immediately fixed in PBS containing 3% paraformaldehyde and 2% sucrose, embedded in O.C.T. compound (VWR chemicals), frozen at −80°C and sectioned by cryostat for immunofluorescence and imaging.

### Plasmids and Constructions

P0 rat cDNA (kind gift from Greg Lemke and Richard Axel), pTK-Slug (Addgene plasmid 36986 [47]), pLVCT-tTR-KRAB (Addgene plasmid 11643 [48]), pCMV-rat NFasc155-Flag (kind gift from Peter Brophy), pBK/CMV-Caspr and Contactin-GFP (kind gifts from Catherine Faivre-Sarrailh), Neurofascin-186-HA (Addgene plasmid 31061 [49]), pFUSE-hIgG1-Fc2 (InvivoGen).

GFP, P0, and P0-myc were amplified by PCR using plasmid DNA as a template and primers containing BamH1 (forward primer) and Xba1 (reverse primer) restriction sites at the 5’ end. To amplify P0-myc, the reverse primer also contained the myc sequence. Primers were as follows: GFP forward, 5’-CCGGATCCCGCCACCATGGTGAGCAAGG-3’, GFP reverse 5’-CCTCTAGAGCTCGTCCATGCCGAGAGTG-3’, P0 forward, 5’-CCGGATCCCGCCACCATGGCTCCTGGGGCTCCCTC-3’, P0 reverse 5’-CCTCTAGACTATTTCTTATCCTTGCGAGAC-3’, P0-myc reverse 5’-CCTCTAGACTACAGGTCCTCCTCTGAGATCAGCTTCTGCTCTTTCTTATCCTTGCGAGACTC-3’. pTK-Slug plasmid was digested with BamH1 and Xba1 to excise Slug cDNA and GFP, P0 or P0-myc were ligated into pTK to generate pTK-GFP, pTK-P0, and pTK-P0-myc. pLVCT-tTR-KRAB already contained GFP. To generate pLVCT-tTR-KRAB-P0, and pLVCT-tTR-KRAB-P0-myc, P0 and P0-myc were excised from pTK-P0 and pTK-P0-myc by digestion with Xba1, followed by a blunt, and a digestion with BamH1. pLVCT-tTR-KRAB was digested with Sma1, blunted, and digested again with BamH1 to excise GFP. P0 or P0-myc were then ligated into pLVCT-tTR-KRAB. To generate P0-Fc construct, P0 cDNA corresponding to P0 extracellular domain (P0ex, 30–153 AA) was amplified by PCR using primers containing EcoR1 (forward primer) and BglII (reverse primer) restriction sites at the 5’ end. Primers were as follows: P0ex forward, 5’- CCGGCCGAATTCCGCCACCATGATTGTGGTTTACACGGACAG-3’, P0ex reverse, 5’-CCGGCCAGATCTCCTAGTGGGCACTTTTTCAAAGAC-3’. P0ex was then inserted into pFUSE-hIgG1-Fc2 between EcoR1 and BglII restriction sites to generate pFUSE-hIgG1-P0-Fc2. P0-D6Y-Fc, P0-D32G-Fc, P0-H52Y-Fc and P0-S49L-Fc were generated by site-directed mutagenesis, using P0-Fc as template. Primers were as follows: D6Y forward, 5’–GTGGTTTACACGTACAGGGAAGTC-3’, D6Y reverse, 5’–GACTTCCCTGTACGTGTAAACCAC–3’, D32G forward, 5’- GAATGGGTCTCAGATGGCATCTCTTTTACCTG-3’, D32G reverse, 5’- CAGGTAAAAGAGATGCCATCTGAGACCCATTC-3’, H52Y forward, 5’- GCCATTTCAATCTTCTACTATGCCAAGGGTC-3’, H52Y reverse, 5’- GACCCTTGGCATAGTAGAAGATTGAAATGGC-3’, S49L forward, 5’- GCCGAGATGCCATTTTAATCTTCCACTATGC-3’, S49L reverse, 5’- GCATAGTGGAAGATTAAAATGGCATCTCGGC-3’. To generate pTK-P0-D32G-myc, pTK-H52Y-myc and pTK-P0-S49L-myc, pTK-P0-myc, P0-D32G-Fc, P0-H52Y-Fc and P0-S49L-Fc were digested with BstEII and BsmBI, resulting in the excision of a fragment of about 300 bp located in P0 extracellular domain. The fragments containing the D32G, H52Y, or S49L mutations were re-ligated into pTK-P0-myc instead of the wild type fragment. All constructs were verified by sequencing.

### Electron Microscopy (EM) and ImmunoEM

For EM, processing of mouse sciatic nerves was carried out as previously described [50]. For ImmunoEM, we used the online optimized immunolabeling protocol from EMS, with modifications. Briefly, adult mouse sciatic nerves were fixed in situ with 3% paraformaldehyde and 0.15% glutaraldehyde in 0.1 M phosphate buffer (PB), pH 7.4, for 6 min, and postfixed in the same fixating reagent for 1 h at 4°C. Nerves were then incubated with 1% sodium borohydride for 15 min, permeabilized in 0.05% Triton X-100 in phosphate buffer for 30 min, and blocked in 1% BSA/3% goat serum/0.04% Triton X-100 in PBS for 1 h at 4°C. Nerves were then incubated with chicken anti-P0 antibody (1:500, Aves Labs) overnight at 4°C, washed and incubated with goat antichicken IgG coupled to ultra-small gold particles (1:100, <1 nm, Science Services) overnight at 4°C. Nerves were then postfixed in 2.5% glutaraldehyde for 2 h and submitted to silver enhancement using Aurion R-Gent SE-EM (Science Services) for 1 h. Nerves were then washed in ddH2O and incubated in 0.5% OsO4 for 1 h at room temperature. Nerves were then dehydrated and embedded in resin, and ultrasections (70-nm thick) were made, as described [50]. No contrasting reagent was applied. Images were acquired using a Philips CM 100 BIOTWIN equipped with a Morada side-mounted digital camera (Olympus).

### Western Blot and Immunoprecipitation (IP)

Sciatic nerves were dissected and the perineurium removed. Tissues were lysed, and IPs were carried out as described [51], with the following modifications: we used PrecipHen beads (Aves Labs, 30 μl of beads/IP for 1 h at 4°C on a rotating wheel) instead of Protein A/G Plus agarose beads, and additional washes were carried out, as recommended by the manufacturer. IPs were then analyzed by Western blotting as previously described [51].

Primary antibodies used: HDAC1 (rabbit, 1:2,000, Genetex), HDAC2 (mouse, 1:2,000, Sigma Aldrich), Sox10 (mouse, 1:500, R&D Systems), GAPDH (glyceraldehyde-3-phosphate-dehydrogenase, mouse, 1:5,000, Hytest Ltd and Genetex), P0 (rabbit, 1:2,000, kind gift from David Colman, and chicken, 1:1,000, Novus Biological or Aves Labs), Caspr (rabbit, 1:1,000, Santa-Cruz Biotechnology), total Neurofascins (rabbit, 1:1,000, kind gift from Laurence Goutebroze), Krox20 (rabbit, 1:500, Covance), MBP (rat, 1:500, Serotec), MAG (myelin-associated glycoprotein, rabbit, 1:1,000, Zymed), ABC (mouse, 1:500, Chemicon), beta-actin (mouse, 1:5,000, Sigma), Nav1.6 Na^+^ channel (mouse, 1:6, clone number K87A/10, UC Davis/NIH NeuroMab Facility), Kv1.2 K+ channel (mouse, 1:1,000, clone number K14/16, UC Davis/NIH NeuroMab Facility), Contactin-1 (goat, 1:200, R&D Systems), Gliomedin (rabbit, 1:500, Santa-Cruz Biotechnology). Two micrograms of chicken anti-P0 antibody or of chicken IgY were used per IP.

All secondary antibodies were from Jackson ImmunoResearch: light chain-specific goat anti-mouse-HRP (horse radish peroxidase), goat anti-rabbit-HRP, and goat anti-rat-HRP, and heavy chain-specific goat anti-chicken-HRP were used.

### Immunofluorescence and 3-D Reconstruction, In Situ Hybridization, TUNEL, and BrdU Assays

DRG cultures were fixed with 4% paraformaldehyde (PFA) for 15 min at RT and were washed twice with PBS. Mouse sciatic nerves were fixed in situ with 4% PFA for 10 min, dissected, embedded in O.C.T. Compound (Tissue-Tek and VWR chemicals), and frozen at −80°C. For immunofluorescence, DRG cultures and peripheral nerve cryosections (5-μm thick, mouse and human) were permeabilized and blocked for 30 min at RT in blocking buffer (0.3% Triton X-100/ 10% Goat serum or 2% BSA for primary antibodies raised in goat/PBS), and incubated with primary antibodies overnight at 4°C in blocking buffer. For staining of MBP, samples were first incubated with 100% methanol at −20°C for 5 min, prior permeabilization and blocking step. For staining of HDAC1, sciatic nerve cryosections were first incubated with acetone at −20°C for 10 min, prior permeabilization and blocking step. For staining of HDAC2, sciatic nerve cryosections were first incubated with 70% Ethanol for 5 min at room temperature, washed with PBS and incubated for 40 s with 40 μg/ml Proteinase K, prior incubation with blocking buffer.

Primary antibodies: CD68 (rat, 1:200, Serotec), P0 (mouse, 1:500, Astexx, and chicken, 1:500, Novus Biological or Aves Labs), MBP (rat, 1:50, Serotec), GFP (rabbit, 1:500, Abcam, and mouse, 1:200, clone number N86/8, UC Davis/NIH NeuroMab Facility), Caspr and Kv1.2 (mouse, 1:200, clones K65/35 and K14/16, UC Davis/NIH NeuroMab Facility), Caspr (rabbit, 1:100, Abcam), total Neurofascins (rabbit, 1:200, kind gift from Laurence Goutebroze), NFasc186 (guinea pig, 1:500, kind gift from Manzoor Bhat, and rabbit, 1:300, Abcam), Myc (mouse, 1:200, kind gift from Wilhelm Krek, and rabbit, 1:200, Genetex), NF M (chicken, 1:200, Genetex, and rabbit, 1:200, Millipore), Ankyrin-G (mouse, 1:200, clone number N106/36, UC Davis/NIH NeuroMab Facility, and mouse, 1:100, Santa-Cruz Biotechnology), Nav1.6 (rabbit, 1:200, Chemicon International), Gliomedin (rabbit, 1:500, kind gift from Elior Peles), Contactin (goat, 1:40, R&D Systems), phospho-ERM (rabbit, 1:250, Abcam), NrCam (rabbit, 1:200, Abcam), beta-Dystroglycan (mouse, 1:50, Novocastra), HDAC1 (rabbit, 1:200, Genetex), HDAC2 (rabbit, 1:200, Santa-Cruz Biotechnology). All secondary antibodies were from Jackson ImmunoResearch.

Immunofluorescence intensities of DRG myelinating cultures were quantified using Cell Profiler 2.0 software (Broad Institute) that calculates the area occupied by staining in the image, after applying a threshold.

3-D reconstructions of immunofluorescence signals were carried out using Imaris software (Bitplane) for surfaces and selected volumes rendering.

In situ hybridization with digoxigenin (DIG)-labeled riboprobes was carried out on cryosections (10-μm thick), as described [52]. Hybridization signals were visualized with NBT/BCIP (Roche Diagnostics). Antisense riboprobes were labelled with digoxigenin, according to the manufacturer’s instructions (Roche Diagnostics).

TUNEL assays were performed on sciatic nerve cryosections (10-μm thick) according to the manufacturer’s instructions (Roche). For BrdU assays, sciatic nerve cryosections (5-μm thick) were first postfixed with 4% PFA for 15 min at room temperature, incubated 5 min with 70% ethanol, and 10 min at 37°C with 2M HCl for DNA denaturation. Sections were then incubated with 40 μg/ml Proteinase K for 30–40 sec, permeabilized 2 x 5 min with 0.1% Tween/PBS, incubated in blocking buffer (30% goat serum/PBS) for 1 h at room temperature, and with mouse anti-BrdU antibody (1:20, Roche) diluted in blocking buffer overnight at 4°C. Sections were then incubated with FITC-goat antimouse IgG (1:20, Roche) for 1 h at room temperature, and labeled with DAPI for 5 min before mounting in Citifluor (Agar Scientific).

### DRG Myelinating Cultures

DRG explant cultures from E13.5 *plp*-dKO embryos and control littermates were prepared as described [53], with modifications: after 10 d in culture, myelination was induced by ascorbic acid and DRG were maintained in myelination-promoting medium for a maximum of 24 d. When applicable, 0.5 μl of highly concentrated lentiviruses was added in Neurobasal medium to DRG explants after 4 d in culture. Fifty ng/ml doxycyclin was added to the culture after 10 d in myelination-promoting medium to induce expression of GFP, P0, or P0-myc by lentiviruses, and 4-hydroxy-tamoxifen was added 4 d later to induce ablation of HDAC1 and HDAC2. Doxycyclin was continuously added, while 4-hydroxy-tamoxifen was added only twice to the medium. DRG cultures were allowed to reach a myelination plateau before tamoxifen was added.

### Generation of Lentiviruses

To produce highly concentrated lentiviral particles, six 15-cm dishes of 95% confluent HEK293T cells were cotransfected with each lentiviral construct together with the packaging constructs pLP1, pLP2, and pLP/VSVG (Invitrogen) using Lipofectamine 2000 (Invitrogen), according to the recommendations of the manufacturer (ViraPower Lentiviral Expression Systems Manual). Lentiviral particles of the six dishes were pooled, purified, and concentrated by ultracentrifugation, resuspended in 45 μl PBS, aliquoted, and stored at −80°C.

### Production of Fc Particles and Adhesion Assay

To produce P0-Fc and control-Fc (neg-Fc) particles, HEK293T cells were transfected with pFUSE-hIgG1-P0-Fc2 and pFUSE-hIgG1-Fc2, respectively, using Lipofectamine (Invitrogen) in culture medium containing 1% Ultra-low IgG FBS (Invitrogen). pFUSE-hIgG1-Fc2 contains an IL2 signal sequence that leads to secretion of the Fc particles. Culture medium that contained secreted P0-Fc and neg-Fc particles was collected 72 h after transfection and concentrated on Centricon Plus-70 10K (for neg-Fc) or 30K (for P0-Fc) filter devices (Millipore). The size and relative concentration of P0-Fc, neg-Fc, and P0 mutants-Fc were verified and analyzed by western blot (S12 Fig).

To carry out adhesion assays, HEK293T cells were transfected with either pTK-P0-myc, pCMV-rat NFasc155-Flag, pBK/CMV-Caspr, Contactin-GFP, or NFasc186-HA to express either P0-myc, NFasc155, Caspr, Contactin, or NFasc186, respectively. Forty-eight hours after transfection, cells were incubated with P0-Fc, P0-D6Y-Fc, P0-D32G-Fc, P0-H52Y-Fc, P0-S49L-Fc, or neg-Fc in 1% Ultra-low IgG FBS/DMEM for 1 h at 37°C, 5% CO_2_/95% air. Cells were then washed three times with PBS and incubated for 1 h at RT with Alexa Fluor 488-AffiniPure Goat Anti-Human IgG (1:500 in PBS, Jackson ImmunoResearch), or Cy3-AffiniPure Goat Anti-Human IgG (1:500 in PBS, Jackson ImmunoResearch) for HEK293T cells expressing Contactin-GFP. Cells were washed again three times with PBS and fixed with 4% PFA for 15 min at RT. After three washes with PBS, cells were incubated overnight at 4°C in PBS containing primary antibodies against either total Neurofascins (Rabbit, 1:200), Caspr (Mouse, 1:100), Contactin (Goat, 1:40) or P0 (Chicken, 1:200), or in PBS/0.3% Triton X-100 containing primary antibody against Myc (Mouse, 1:200). After three washes with PBS, cells were incubated for 1 h at RT with Cy5-Affinipure Goat Anti-Rabbit IgG or Alexa Fluor® 647-AffiniPure Goat Anti-Mouse IgG (1:500, Jackson ImmunoResearch) diluted in PBS or PBS/0.3% Triton X-100 for Myc staining. Cells were then washed in PBS, incubated with DAPI for 5 min, washed again, and mounted in Citifluor. Photos were acquired using a Leica TCS SP-II confocal microscope. Single optical sections are shown in Figs 7 and 8, and z-series projections are shown in S13 and S14 Figs.

### RT-PCR

Isolation of RNA was carried out using Trizol reagent (Invitrogen), and cDNA was produced using M-MLV Reverse Transcriptase (Promega), according to the manufacturer’s recommendations. Quantitative real-time PCR analyses were performed with an ABI 7000 Sequence Detection System (Applied Biosystems) using FastStart SYBR Green Master (Roche), according to manufacturer’s recommendations.

Primer sequences were as follows: for NFasc primer pair 1 (specific to NFasc155), *forward* 5'-GACTCGTCCTTAAGAAACCAC-3', *reverse* 5'-GGCAATGCATTCCAGCAGCAG-3'; for NFasc primer pair 2, *forward* 5’- CTTCACCCTCAAGGTCCTCAC-3’, *reverse* 5’- GGCAATGCATTCCAGCAGCAG-3’; for GAPDH, *forward* 5'-CGTCCCGTAGACAAAATGGT-3', *reverse* 5'-TTGATGGCAACAATCTCCAC-3'. The amplification program was as follows: 5 min at 96°C; 40 steps of 30 s at 96°C, 30 s at 59.5°C, 30 s at 72°C; and 5 min at 72°C. A post-PCR dissociation step was added to verify the specificity of the amplification products.

### Statistical Analyses and Data Availability

Experiments were performed at least three times, and *p*-values were calculated using Student's *t* tests. Data used to create Fig 1C–1H, Fig 2A and 2D, Fig 3A, 3G and 3H, Fig 4A and 4B, Fig 5B, 5E, 5G and 5I, Fig 6F, Fig 7F, Fig 9B and 9E, S1A–S1C Fig, S2B–S2D Fig, S3A–S3E Fig, S5C Fig, S6A Fig, S7B and S7C Fig, S17B and S17E Fig, and S18 Fig are deposited in the Dryad repository: http://dx.doi.org/10.5061/dryad.8f1bt [54].

## Supporting Information

S1 Fig Unchanged axon diameter in dKO and HDAC1/2 expression in single KO. (A) Percentage of axons of different calibers in control and dKO nerves measured using electron micrographs of 3 control and 3 dKO mice at 8 wk post-tamoxifen. For quantification, all axons of a randomly chosen area of 0.0108 mm^2^ (~300 axons) were quantified per animal. (B–C) Western blot of HDAC2 (B) and HDAC1 (C) in control, H1KO and H2KO sciatic nerve lysates at 7 d post-tamoxifen, and quantification of protein levels normalized to the loading control GAPDH in mutants compared to controls (= 100%) (three animals per genotype were used). The dashed lines indicate that lysates have been run on the same gel, but not on consecutives lanes. *P*-values (unpaired (A) or paired (B–C) two-tailed (unless stated otherwise in the figure) Student's *t* test): * = *p* < 0.05, ** = *p* < 0.01, *** = *p* < 0.001, error bars = SEM.
(TIF)

S2 Fig No demyelination and unchanged MBP levels, but decreased P0 levels at 5 wk post-tamoxifen in dKO and at 8 wk post-tamoxifen in H2KO sciatic nerves. (A) Electron micrograph of ultrathin cross sections of dKO sciatic nerve at 5 wk post-tamoxifen. Sciatic nerves of 3 dKO mice were analyzed and no demyelinated or remyelinated axon or macrophage were found. (B–D) Western blot of P0 (B,C) and MBP (D) in control and dKO sciatic nerve lysates at 5 wk post-tamoxifen (B,D), and in control, H1HTZ, H1KO, H2HTZ, and H2KO at 8 wk post-tamoxifen (C), and quantification of protein levels normalized to the loading control GAPDH in mutants compared to controls (= 100%) (3 animals per genotype were used). In (B,D), the dashed lines indicate that lysates were run on the same gel but not on consecutive lanes. *P*-values (unpaired (B,C,D) or paired (C, HDAC2 single mutants) two-tailed (unless stated otherwise in the figure) Student's *t* test): * = *p* < 0.05, error bars = SEM.
(TIF)

S3 Fig Further characterization of dKO phenotype at the molecular level. Western blots of MBP (A), Sox10 (B), Krox20 (C), MAG (D), and ABC (E) in lysates of control (Co) and dKO sciatic nerves at 8 wk post-tamoxifen, and quantification of protein levels normalized to GAPDH or beta-actin loading control in dKOs compared to controls (= 100%). For each experiment, three control and three dKO animals were used. *P*-values (paired two-tailed Student's *t* test): * = *p* < 0.05, error bars = SEM.
(TIF)

S4 Fig No difference in apoptosis or proliferation in control and dKO sciatic nerves. TUNEL assay (red, A) and BrdU assay (green, B) to detect apoptotic and proliferating cells, respectively, in longitudinal cryosections of control and dKO sciatic nerves at 8 wk (A) and 5 wk (B) post-tamoxifen. Nuclei are labeled in blue with DAPI.
(TIF)

S5 Fig Neurofascins and Caspr in H1HTZ, H2HTZ, and H1KO nodes/paranodes are similar to control and mildly affected in H2KO sciatic nerves. (A) Coimmunofluorescence of Caspr (green) and Contactin (red) and (B) immunofluorescence of total neurofascins (NFasc, green) in longitudinal cryosections of control and dKO sciatic nerves at 8 wk post-tamoxifen, and (C) quantification of nodes lacking NFasc186 and paranodes with low and/or asymmetric Caspr (3 animals per genotype were quantified, at least 50 nodes/paranodes per animal and 150 per genotype). *P*-values (unpaired two-tailed (unless stated otherwise in the figure) Student's *t* test): * = *p* < 0.05, error bars = SEM. Arrows indicate nodes of Ranvier. Scale bars = 5 μm.
(TIF)

S6 Fig Protein levels and localization of components of juxtaparanodes, paranodes, and nodes of Ranvier in control and dKO sciatic nerves. (A) Western blots of Kv1.2, Nav1.6, and Gliomedin, and quantification normalized to the loading control beta-actin or GAPDH. Results are presented as percentage of the control (= 100%) for Kv1.2 or as ratios to GAPDH for Nav1.6 and Gliomedin. For each experiment, 3 control and 3 dKO animals were used. *P*-values (paired (for Kv1.2) or unpaired two-tailed (unless stated otherwise in the figure) Student's *t* test): * = *p* < 0.05, ** = *p* < 0.01, error bars = SEM. (B–G) Coimmuofluorescence of Contactin (CNTN, red) with (B) Ankyrin G (AnkG, green), (C) Nav1.6 (green), (D) NrCam (green), (E) beta-dystroglycan (βDG, green), (F) phospho-Ezrin-Radixin-Moesin (pERM, green), or Gliomedin (green) on longitudinal cryosections of control and dKO sciatic nerves at 8 wk post-tamoxifen. Z-series projections of confocal images are shown. Arrows show the position of nodes of Ranvier. Scale bars = 5 μm.
(TIF)

S7 Fig Significant increase of abnormal nodes and paranodes in dKO at 5 wk post-tamoxifen. (A) Immunofluorescence of neurofascins (green) or Caspr (green) on longitudinal cryosections of control and dKO sciatic nerves at 5 wk post-tamoxifen. Z-series projections of confocal stacks are shown. Arrows show the position of nodes of Ranvier. Scale bars = 5 μm. (B) Quantification of nodes lacking NFasc186 and paranodes with low or asymmetric Caspr. (C) Quantification of normal, elongated, and abnormal (low intensity, asymmetric, irregular shape) paranodes, based on NFasc staining in paranodes. Three animals per genotype were used, 100–200 nodes and paranodes counted per animal, 350 to 500 counted per genotype. *P*-values (unpaired two-tailed (unless stated otherwise in the figure) Student's *t* test): * = *p* < 0.05, error bars = SEM.
(TIF)

S8 Fig Efficient loss of HDAC1 and HDAC2 induced by tamoxifen treatment, and myelin degradation in *plp*-dKO DRG cultures. (A) HDAC1 (green) and HDAC2 (red) coimmunofluorescence in myelinated DRG cultures of control and *plp*-dKO embryos at 8 d post-tamoxifen treatment. Note that neuron nuclei (round nuclei marked by open arrowheads) express high levels of HDAC1 and HDAC2 compared to Schwann cell nuclei (small elongated nuclei marked by arrows). Smaller images are magnifications of the white boxes depicted in the larger images. Nuclei are labeled in blue with DAPI. (B) Photographs of P0 immunofluorescence in myelinated control and *plp*-dKO DRG cultures 10 d post-tamoxifen treatment. The punctuated P0 signal in *plp*-dKO DRG suggests myelin breakdown. DRG of at least three control and three *plp*-dKO embryos were analyzed, and representative pictures are shown.
(TIF)

S9 Fig Loss of neurofascins in nodes/paranodes of P0 HTZ and P0 KO sciatic nerves. Coimmunofluorescence of total neurofascins (NFasc, red) with P0 (green) (A,B) or NFasc186 (blue), Contactin (red) and P0 (green) (A,C) on longitudinal cryosections of control, P0 HTZ and P0 KO adult (10-month old) sciatic nerves. Representative photographs of 2 control, 4 P0 HTZ, and 2 P0 KO mice are shown. Asterisks mark the position (lateral dimension) of the node of Ranvier. In (B,C), magnifications of single paranodal/nodal regions are shown. Scale bars = 5 μm.
(TIF)

S10 Fig P0-myc rescues neurofascins in *plp*-dKO DRG cultures. Coimmunofluorescence of total neurofascins (NFasc, green) and MBP (red) in control or *plp*-dKO myelinated DRG cultures transduced with lentiviruses expressing GFP (Control and *plp*-dKO) or P0-myc (*plp*-dKO + P0-myc). Z-series projections of confocal stacks are shown. Images on the right are magnifications of white boxes depicted on the left images highlighting heminodes or nodes. DRG of three control and three *plp*-dKO embryos were analyzed and representative images are shown.
(TIF)

S11 Fig P0 localization in myelinated fibers of DRG cultures and in human sciatic nerves. (A) Confocal images (z-series projections) of P0 (red) and MBP (green) coimmunofluorescence in myelinated mouse DRG cultures. Overlay appears yellow. DRG cultures of six control embryos were analyzed, and a representative P0-positive fiber is shown. White dashed lines delineate MBP signal that is apparently restricted to internodes, whereas P0 signal extends further between two internodes. Scale bar = 5 μm. (B–C) Coimmunofluorescence of total neurofascins (NFasc, red) and P0 (green) (B) or of NFasc186 (blue), Contactin (red), and P0 (green) (C) on longitudinal cryosections of adult human peripheral nerves. Median, tibial, and/or sciatic nerves of three human individuals were analyzed and representative pictures of sciatic nerves are shown. P0 was abundant in all paranodal/nodal regions we observed. Z-series projections and single optical sections are shown. Asterisks mark the position (lateral dimension) of the node of Ranvier. Scale bars = 5 μm in (B) and 10 μm in (C).
(TIF)

S12 Fig Validation of control-Fc, wild-type P0-Fc, and P0-Fc mutants production. Western blot of purified control Fc (neg-Fc) and P0-Fc particles (A) and of P0-Fc, P0-D6Y-Fc, P0-D32G-Fc, P0-H52Y-Fc, P0-D46G-Fc (silent mutation for binding to P0 and neurofascins), and P0-S49L-Fc particles (B) with antihuman IgG antibody coupled to HRP. Increasing amounts of purified neg-Fc and P0-Fc are loaded in (A) and 10 μl of each P0-Fc wild type or mutant are loaded in (B). The expected size of neg-Fc is 25.5 kDa, while the expected size of P0-Fc is around 38 kDa.
(TIF)

S13 Fig P0 extracellular domain binds to P0, NFasc155, and NFasc186 extracellular domains. (A–E) Adhesion assay in HEK293T cells. Confocal images of P0-Fc or control-Fc (Neg-Fc) particles (green, or false-colored green for Contactin-GFP) and P0 (A, red), neurofascins (B and C, red), Caspr (D, red), Contactin (E, red). or GFP (E, false-colored turquoise) coimmunofluorescence in HEK293T cells expressing P0-myc (A), NFasc155 (B), NFasc186 (C), Caspr (D), or Contactin-GFP (E). Overlays appear yellow. Nuclei are labeled in blue with DAPI. All labelings were carried out on unpermeabilized cells, and z-series projections are shown. Each experiment was done at least three times, and representative pictures are shown. Scale bars = 5 μm.
(TIF)

S14 Fig The three P0 mutations D6Y, D32G, and H52Y impair binding to one or both neurofascins, but not to P0, and S49L maintains binding to both neurofascins and to P0. Adhesion assay in HEK293T cells. Confocal images of P0-D6Y-Fc, P0-D32G-Fc, P0-H52Y-Fc, or P0-S49L-Fc particles (green) and neurofascins or P0 (red) coimmunofluorescence in HEK293T cells expressing P0-myc (A), NFasc155 (B), or NFasc186 (C). Overlays appear yellow. Nuclei are labeled in blue with DAPI. All labelings were carried out on unpermeabilized cells, and z-series projections are shown. At least three independent experiments were analyzed for each panel, and representative pictures are shown. Scale bars = 5 μm.
(TIF)

S15 Fig P0 mutants are targeted to the plasma membrane of differentiated primary rat Schwann cells. Immunofluorescence of Myc (green) counterstained with DAPI (blue) to detect myc-tagged wild-type P0 (P0-myc), D32G, H52Y, and S49L P0 mutants in primary rat Schwann cells transfected with either pTK-P0-myc, pTK-D32G-myc, pTK-H52Y-myc, or pTK-S49L-myc and cultured in differentiating conditions for 3 d. All Myc positive cells (~50 cells per construct) showed plasma membrane localization of wild-type P0-myc and mutants.
(TIF)

S16 Fig Validation of Myc staining specificity in myelinated *plp*-dKO DRG cultures efficiently transduced with lentiviruses expressing GFP. Coimmunofluorescence of MBP (Magenta, rat antibody) and Myc (red, mouse antibody), and GFP fluorescence (green) in myelinated *plp*-dKO DRG cultures transduced with lentiviruses expressing GFP and treated with tamoxifen for 10 d. Even at high exposure, Myc staining did not cross-react with MBP staining. To avoid cross-reactivity, we used multiple labeling (adsorbed against many animal species, including rat for antimouse and mouse for antirat) secondary antimouse and antirat antibodies. Antibody concentrations and staining protocol (buffers, incubation times and temperature, washes) were the same as for stainings presented in Fig 9A. Nuclei are labeled in blue with DAPI. Pictures on the right (single optical sections) are magnifications of the white boxes depicted on left images (z-series projections). Arrows indicate Schwann cell nuclei of myelinated fibers, arrowheads indicate MBP staining. DRG of three *plp*-dKO embryos were analyzed. None of the MBP-positive fibers were labeled by Myc staining.
(TIF)

S17 Fig No difference of MBP levels or of percentage of intact nodes/heminodes between control DRG cultures transduced with lentiviruses carrying GFP, H52Y, D32G, S49L P0 mutants, or P0-myc. Coimmunofluorescence of MBP (red) and (A) neurofilament (NF, green) and Myc or GFP fluorescence (blue), or (C) neurofascins (NFasc, green), or (D) Caspr (green) in myelinated HDAC1/2 control DRG cultures transduced with lentiviruses expressing either GFP, H52Y-myc, D32G-myc, S49L-myc, or P0-myc, and treated with tamoxifen for 10 d after completion of myelination. Arrows indicate paranodes/nodes. In (B), quantification of MBP fluorescence intensity normalized to NF and compared to GFP (set to 1). DRG of three control embryos were quantified, four coverslips per embryo were analyzed, and representative pictures are shown. In (E), the graph represents the percentage of intact (high NFasc levels) nodes and heminodes. DRG of three control embryos were quantified, four coverslips per control, 40 to 80 nodes/heminodes counted per control per virus. Error bars = SEM.
(TIF)

S18 Fig NFasc transcript levels are unchanged in dKO sciatic nerves. Graph showing mRNA levels of NFasc155 (primer pair 1) and NFasc (presumably also NFasc155, primer pair 2) normalized to GAPDH and measured by real-time qPCR with two different primer pairs in dKO compared to control (= 100%) sciatic nerves at 5 wk post-tamoxifen, before the influx of macrophages, but when P0 protein levels were already reduced (see S2B Fig) and the localization of NFasc at the nodes/paranodes was already significantly affected (see S7 Fig) in dKO sciatic nerves. Sciatic nerves of three dKO and three control littermate animals were used, and no significant difference was detected.
(TIF)

S19 Fig Summary of HDAC1/2-dependent P0 functions in adult PNS paranodes/nodes. P0 interacts with NFasc155 in paranodes and with NFasc186 in nodes of Ranvier. The dashed circle around NFasc155, P0, and NFasc186 indicates the hypothetical simultaneous interaction of P0 with both NFasc155 and NFasc186. Ablation of HDAC1/2 in adult SCs leads to loss of P0, which results in 1) mislocalization and decrease of NFasc155 levels, loss of Caspr and septate-like junctions, and mislocalization of Kv1.2 in paranodes, and 2) loss of NFasc186 in nodes of Ranvier. Dystroglycan, NrCam, pERM (not represented in the drawing), and Contactin remain in paranodes of dKO nerves.
(TIF)

## Abbreviations

ABC: active beta-catenin
CMT: Charcot-Marie-Tooth disease
DRG: dorsal root ganglia
FNIII: fibronectin type III
GFP: green fluorescent protein
HDAC: histone deacetylase
IG: immunoglobulin
IP: immunoprecipitation
MAG: myelin-associated glycoprotein
MBP: myelin basic protein
NF: neurofilament
P0: myelin protein zero
PNS: peripheral nervous system
SC: Schwann cell

## References

[1] Craner MJ, Newcombe J, Black JA, Hartle C, Cuzner ML, Waxman SG. Molecular changes in neurons in multiple sclerosis: altered axonal expression of NaV1.2 and NaV1.6 sodium channels and Na^+^/Ca^+^ exchanger. Proc Natl Acad Sci USA. 2004;101: 8168–8173. 15148385 10.1073/pnas.0402765101 PMC419575

[2] Mathey EK, Derfuss T, Storch MK, Williams KR, Hales K, Woolley DR, et al Neurofascin as a novel target for autoantibody-mediated axonal injury. J Exp Med. 2007;204: 2363–2372. 17846150 10.1084/jem.20071053 PMC2118456

[3] Susuki K, Rasband MN, Tohyama K, Koibuchi K, Okamoto S, Funakoshi K, et al Anti-GM1 antibodies cause complement-mediated disruption of sodium channel clusters in peripheral motor nerve fibers. J Neurosci. 2007;27: 3956–3967. 17428969 10.1523/JNEUROSCI.4401-06.2007 PMC6672537

[4] Devaux JJ, Odaka M, Yuki N. Nodal proteins are target antigens in Guillain-Barré syndrome. J Peripher Nerv Syst. 2012;17: 62–71. 10.1111/j.1529-8027.2012.00372.x 22462667

[5] Meadows LS, Malhotra J, Loukas A, Thyagarajan V, Kazen-Gillespie KA, Koopman MC, et al Functional and biochemical analysis of a sodium channel beta1 subunit mutation responsible for generalized epilepsy with febrile seizures plus type 1. J Neurosci. 2002;22: 10699–10709. 12486163 10.1523/JNEUROSCI.22-24-10699.2002 PMC6758463

[6] Cruz DA, Weaver CL, Lovallo EM, Melchitzky DS, Lewis DA. Selective alterations in postsynaptic markers of chandelier cell inputs to cortical pyramidal neurons in subjects with schizophrenia. Neuropsychopharmacology 2009;34: 2112–2124. 10.1038/npp.2009.36 19322171 PMC2721024

[7] Salzer JL, Brophy PJ, Peles E. Molecular domains of myelinated axons in the peripheral nervous system. Glia. 2008;56: 1532–1540. 10.1002/glia.20750 18803321

[8] Buttermore ED, Thaxton CL, Bhat MA. Organization and maintenance of molecular domains in myelinated axons. J Neurosci Res. 2013;91: 603–622. 10.1002/jnr.23197 23404451 PMC4049519

[9] Eshed-Eisenbach Y, Peles E. The making of a node: a co-production of neurons and glia. Curr Opin Neurobiol. 2013;23: 1049–1056. 10.1016/j.conb.2013.06.003 23831261 PMC3875870

[10] de Ruijter AJ, van Gennip AH, Caron HN, Kemp S, van Kuilenburg AB. Histone deacetylases (HDACs): characterization of the classical HDAC family. Biochem J. 2003;370: 737–749. 12429021 10.1042/BJ20021321 PMC1223209

[11] Michan S, Sinclair D. Sirtuins in mammals: insights into their biological function. Biochem J. 2007;404: 1–13. 17447894 10.1042/BJ20070140 PMC2753453

[12] Glozak MA, Sengupta N, Zhang X, Seto E. Acetylation and deacetylation of non-histone proteins. Gene. 2005;363: 15–23. 16289629 10.1016/j.gene.2005.09.010

[13] Yao YL, Yang WM. Beyond histone and deacetylase: an overview of cytoplasmic histone deacetylases and their non-histone substrates. J Biomed Biotechnol. 2011;2011: 146493 10.1155/2011/146493 21234400 PMC3014693

[14] Jacob C, Lötscher P, Engler S, Baggiolini A, Varum Tavares S, Brügger V, et al HDAC1 and HDAC2 control the specification of neural crest cells into peripheral glia. J Neurosci. 2014;34: 6112–6122. 10.1523/JNEUROSCI.5212-13.2014 24760871 PMC3996228

[15] Jacob C, Christen CN, Pereira JA, Somandin C, Baggiolini A, Lötscher P, et al HDAC1 and HDAC2 control the transcriptional program of myelination and the survival of Schwann cells. Nat Neurosci. 2011;14: 429–436. 10.1038/nn.2762 21423190

[16] Jacob C, Lebrun-Julien F, Suter U. How histone deacetylases control myelination. Mol Neurobiol. 2011;44: 303–312. 10.1007/s12035-011-8198-9 21861092

[17] Chen Y, Wang H, Yoon SO, Xu X, Hottiger MO, Svaren J, et al HDAC-mediated deacetylation of NF-κB is critical for Schwann cell myelination. Nat Neurosci. 2011;14: 437–441. 10.1038/nn.2780 21423191 PMC3074381

[18] Shapiro L, Doyle JP, Hensley P, Colman DR, Hendrickson WA. Crystal structure of the extracellular domain from P0, the major structural protein of peripheral nerve myelin. Neuron. 1996;17: 435–449. 8816707 10.1016/s0896-6273(00)80176-2

[19] Shy ME, Jáni A, Krajewski K, Grandis M, Lewis RA, Li J, et al Phenotypic clustering in MPZ mutations. Brain. 2004;127: 371–384. 14711881 10.1093/brain/awh048

[20] Leone DP, Genoud S, Atanasoski S, Grausenburger R, Berger P, Metzger D, et al Tamoxifen-inducible glia-specific Cre mice for somatic mutagenesis in oligodendrocytes and Schwann cells. Mol Cell Neurosci. 2003;22: 430–440. 12727441 10.1016/s1044-7431(03)00029-0

[21] Yamaguchi T, Cubizolles F, Zhang Y, Reichert N, Kohler H, Seiser C, et al Histone deacetylases 1 and 2 act in concert to promote the G1-to-S progression. Genes Dev. 2010;24: 455–469. 10.1101/gad.552310 20194438 PMC2827841

[22] Ribeiro S, Napoli I, White IJ, Parrinello S, Flanagan AM, Suter U, et al Injury signals cooperate with Nf1 loss to relieve the tumor-suppressive environment of adult peripheral nerve. Cell Rep. 2013;5: 126–136. 10.1016/j.celrep.2013.08.033 24075988

[23] Montgomery RL, Davis CA, Potthoff M, Haberland M, Fielitz J, Qi X, et al Histone deacetylases 1 and 2 redundantly regulate cardiac morphogenesis, growth, and contractility. Genes Dev. 2007;21: 1790–1802. 17639084 10.1101/gad.1563807 PMC1920173

[24] Ye F, Chen Y, Hoang T, Montgomery RL, Zhao XH, Bu H, et al HDAC1 and HDAC2 regulate oligodendrocyte differentiation by disrupting the β-catenin–TCF interaction. Nat Neurosci. 2009;12: 829–838. 10.1038/nn.2333 19503085 PMC2701973

[25] Bhat MA, Rios JC, Lu Y, Garcia-Fresco GP, Ching W, St. Martin M, et al. Axon-glia interactions and the domain organization of myelinated axons requires NeurexinIV/Caspr/Paranodin. Neuron. 2001;30: 369–383. 11395000 10.1016/s0896-6273(01)00294-x

[26] Colombelli C, Palmisano M, Eshed-Eisenbach Y, Zambroni D, Pavoni E, Ferri C, et al Perlecan is recruited by dystroglycan to nodes of Ranvier and binds the clustering molecule gliomedin. J Cell Biol. 2015; 208: 313–329. 10.1083/jcb.201403111 25646087 PMC4315246

[27] Tait S, Gunn-Moore F, Collinson JM, Huang J, Lubetzki C, Pedraza L, et al An oligodendrocyte cell adhesion molecule at the site of assembly of the paranodal axo-glial junction. J Cell Biol. 2000;150: 657–666. 10931875 10.1083/jcb.150.3.657 PMC2175192

[28] Rios JC, Melendez-Vasquez CV, Einheber S, Lustig M, Grumet M, Hemperly J, et al Contactin-associated protein (Caspr) and contactin form a complex that is targeted to the paranodal junctions during myelination. J Neurosci. 2000;20: 8354–8364. 11069942 10.1523/JNEUROSCI.20-22-08354.2000 PMC6773165

[29] Boyle MET, Berglund EO, Murai KK, Weber L, Peles E, Ranscht B. Contactin orchestrates assembly of the septate-like junctions at the paranode in myelinated peripheral nerve. Neuron. 2001;30: 385–397. 11395001 10.1016/s0896-6273(01)00296-3

[30] Charles P, Tait S, Faivre-Sarrailh C, Barbin G, Gunn-Moore F, Denisenko-Nehrbass N, et al Neurofascin is a glial receptor for the Paranodin/Caspr-Contactin axonal complex at the axoglial junction. Curr Biol. 2002;12: 217–220. 11839274 10.1016/s0960-9822(01)00680-7

[31] Sherman DL, Tait S, Melrose S, Johnson R, Zonta B, Court FA, et al Neurofascins are required to establish axonal domains for saltatory conduction. Neuron. 2005;48: 737–742. 16337912 10.1016/j.neuron.2005.10.019

[32] Martini R, Zielasek J, Toyka KV, Giese KP, Schachner M. Protein zero (P0)-deficient mice show myelin degeneration in peripheral nerves characteristic of inherited human neuropathies. Nat Genet. 1995;11: 281–286. 7581451 10.1038/ng1195-281

[33] Samsam M, Frei R, Marziniak M, Martini R, Sommer C. Impaired sensory function in heterozygous P0 knockout mice is associated with nodal changes in sensory nerves. J Neurosci Res. 2002;67: 167–173. 11782960 10.1002/jnr.10115

[34] Ulzheimer JC, Peles E, Levinson SR, Martini R. Altered expression of ion channel isoforms at the node of Ranvier in P0-deficient myelin mutants. Mol Cell Neurosci. 2004;25: 83–94. 14962742 10.1016/j.mcn.2003.09.015

[35] Fratta P, Saveri P, Zambroni D, Ferri C, Tinelli E, Messing A, et al P0S63del impedes the arrival of wild-type P0 glycoprotein to myelin in CMT1B mice. Hum Mol Genet. 2001;20: 2081–2090. 10.1093/hmg/ddr081 PMC3090187 21363884

[36] Pillai AM, Thaxton C, Pribisko AL, Cheng Jr-G, Dupree JL, Bhat MA. Spatiotemporal ablation of myelinating glia-specific neurofascin (Nfasc^NF155^) in mice reveals gradual loss of paranodal axoglial junctions and concomitant disorganization of axonal domains. J Neurosci Res. 2009;87: 1773–1793. 10.1002/jnr.22015 19185024 PMC2837286

[37] Mastaglia FL, Nowak KJ, Stell R, Phillips BA, Edmondston JE, Dorosz SM, et al Novel mutation in the myelin protein zero gene in a family with intermediate hereditary motor and sensory neuropathy. J Neurol Neurosurg Psychiatry 1999;67: 174–179. 10406984 10.1136/jnnp.67.2.174 PMC1736462

[38] Senderek J, Hermanns B, Lehmann U, Bergmann C, Marx G, Kabus C, et al Charcot-Marie-Tooth neuropathy type 2 and P0 point mutations: two novel amino acid substitutions (Asp61Gly; Tyr119Cys) and a possible “hotspot” on Thr124Met. Brain Pathology. 2000;10: 235–248. 10764043 10.1111/j.1750-3639.2000.tb00257.x PMC8098375

[39] Bienfait HME, Baas F, Gabreëls-Festen AAWM, Koelman JHTM, Langerhorst CT, de Visser M, et al Two amino-acid substitutions in the myelin protein zero gene of a case of Charcot–Marie–Tooth disease associated with light-near dissociation. Neuromuscular Disorders. 2002;12: 281–285. 11801400 10.1016/s0960-8966(01)00281-4

[40] Silander K, Meretoja P, Juvonen Vesa, Ignatius Jaakko, Pihko Helena, Saarinen Ari, et al Spectrum of mutations in Finnish patients with Charcot-Marie-Tooth disease and related neuropathies. Human Mutation. 1998;12: 59–68. 9633821 10.1002/(SICI)1098-1004(1998)12:1<59::AID-HUMU9>3.0.CO;2-A

[41] Fabrizi GM, Taioli F, Cavallaro T, Rigatelli F, Simonati A, Mariani G, et al Focally folded myelin in Charcot-Marie-Tooth neuropathy type 1B with Ser49Leu in the myelin protein zero. Acta Neuropathol. 2000;100: 299–304. 10965800 10.1007/s004019900175

[42] Thaxton C, Pillai AM, Pribisko AL, Dupree JL, Bhat MA. Nodes of Ranvier act as barriers to restrict invasion of flanking paranodal domains in myelinated axons. Neuron. 2011;69: 244–257. 10.1016/j.neuron.2010.12.016 21262464 PMC3035172

[43] Amor V, Feinberg K, Eshed-Eisenbach Y, Vainshtein A, Frechter S, Grumet M, et al Long-term maintenance of Na^+^ channels at nodes of Ranvier depends on glial contact mediated by gliomedin and NrCAM. J Neurosci. 2014;34: 5089–5098. 10.1523/JNEUROSCI.4752-13.2014 24719088 PMC3983794

[44] Eshed Y, Feinberg K, Poliak S, Sabanay H, Sarig-Nadir O, Spiegel I, et al Gliomedin mediates Schwann cell-axon interaction and the molecular assembly of the nodes of Ranvier. Neuron. 2005;47: 215–229. 16039564 10.1016/j.neuron.2005.06.026

[45] Kriebel M, Wuchter J, Trinks S, Volkmer H. Neurofascin: a switch between neuronal plasticity and stability. Int J Biochem Cell Biol. 2012;44: 694–697. 10.1016/j.biocel.2012.01.012 22306302

[46] Giese KP, Martini R, Lemke G, Soriano P, Schachner M. Mouse P0 gene disruption leads to hypomyelination, abnormal expression of recognition molecules, and degeneration of myelin and axons. Cell. 1992;71: 565–576. 1384988 10.1016/0092-8674(92)90591-y

[47] Guo W, Keckesova Z, Donaher JL, Shibue T, Tischler V, Reinhardt F, et al Slug and Sox9 cooperatively determine the mammary stem cell state. Cell. 2012;148: 1015–1028. 10.1016/j.cell.2012.02.008 22385965 PMC3305806

[48] Szulc J, Wiznerowicz M, Sauvain MO, Trono D, Aebischer P. A versatile tool for conditional gene expression and knockdown. Nat Methods. 2006;3: 109–116. 16432520 10.1038/nmeth846

[49] Zhang X, Davis JQ, Carpenter S, Bennett V. Structural requirements for association of neurofascin with ankyrin. J Biol Chem. 1998;273: 30785–30794. 9804856 10.1074/jbc.273.46.30785

[50] Pereira JA, Benninger Y, Baumann R, Gonçalves AF, Özçelik M, Thurnherr T, et al Integrin-linked kinase is required for radial sorting of axons and Schwann cell remyelination in the peripheral nervous system. J Cell Biol. 2009;185: 147–161. 10.1083/jcb.200809008 19349584 PMC2700520

[51] Jacob C, Grabner H, Atanasoski S, Suter U. Expression and localization of Ski determine cell type-specific TGFβ signaling effects on the cell cycle. J Cell Biol. 2008;182: 519–530. 10.1083/jcb.200710161 18695043 PMC2500137

[52] Paratore C, Suter U, Sommer L. Embryonic gene expression resolved at the cellular level by fluorescence in situ hybridization. Histochem Cell Biol. 1999;111: 435–443. 10429965 10.1007/s004180050379

[53] Stendel C, Roos A, Kleine H, Arnaud E, Ozçelik M, Sidiropoulos PN, et al SH3TC2, a protein mutant in Charcot-Marie-Tooth neuropathy, links peripheral nerve myelination to endosomal recycling. Brain. 2010;133: 2462–2474. 10.1093/brain/awq168 20826437

[54] Brügger V, Engler S, Pereira JA, Ruff S, Horn M, Welzl H, et al (2015) Data from: HDAC1/2-Dependent P0 Expression Maintains Paranodal and Nodal Integrity Independently of Myelin Stability through Interactions with Neurofascins. Dryad Digital Repository. Openly available via 10.5061/dryad.8f1bt. PMC4583457 26406915

