# Supplementary figures and images for: HDAC1/2-Dependent P0 Expression Maintains Paranodal and Nodal Integrity Independently of Myelin Stability through Interactions with Neurofascins
Source: PLoS Biol. 2015 Sep 25;13(9):e1002258. doi: 10.1371/journal.pbio.1002258 (PMC4583457; doi:10.1371/journal.pbio.1002258)

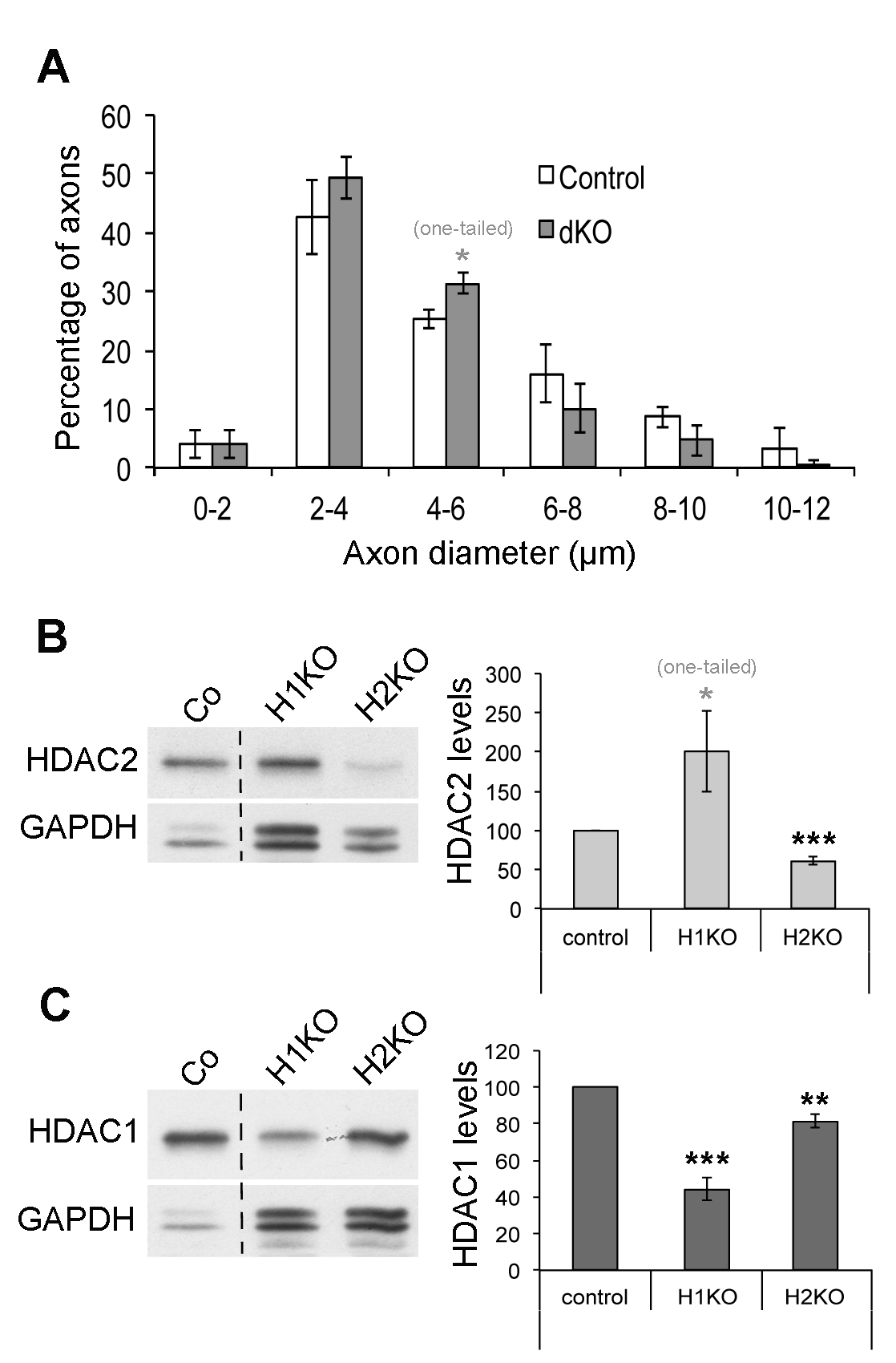

Supplement: S1 Fig — (A) Percentage of axons of different calibers in control and dKO nerves measured using electron micrographs of 3 control and 3 dKO mice at 8 wk post-tamoxifen. For quantification, all axons of a randomly chosen area of 0.0108 mm2 (~300 axons) were quantified per animal. (B–C) Western blot of HDAC2 (B) and HDAC1 (C) in control, H1KO and H2KO sciatic nerve lysates at 7 d post-tamoxifen, and quantification of protein levels normalized to the loading control GAPDH in mutants compared to controls (= 100%) (three animals per genotype were used). The dashed lines indicate that lysates have been run on the same gel, but not on consecutives lanes. P-values (unpaired (A) or paired (B–C) two-tailed (unless stated otherwise in the figure) Student's t test): * = p < 0.05, ** = p < 0.01, *** = p < 0.001, error bars = SEM. (TIF) [file pbio.1002258.s001.tif]

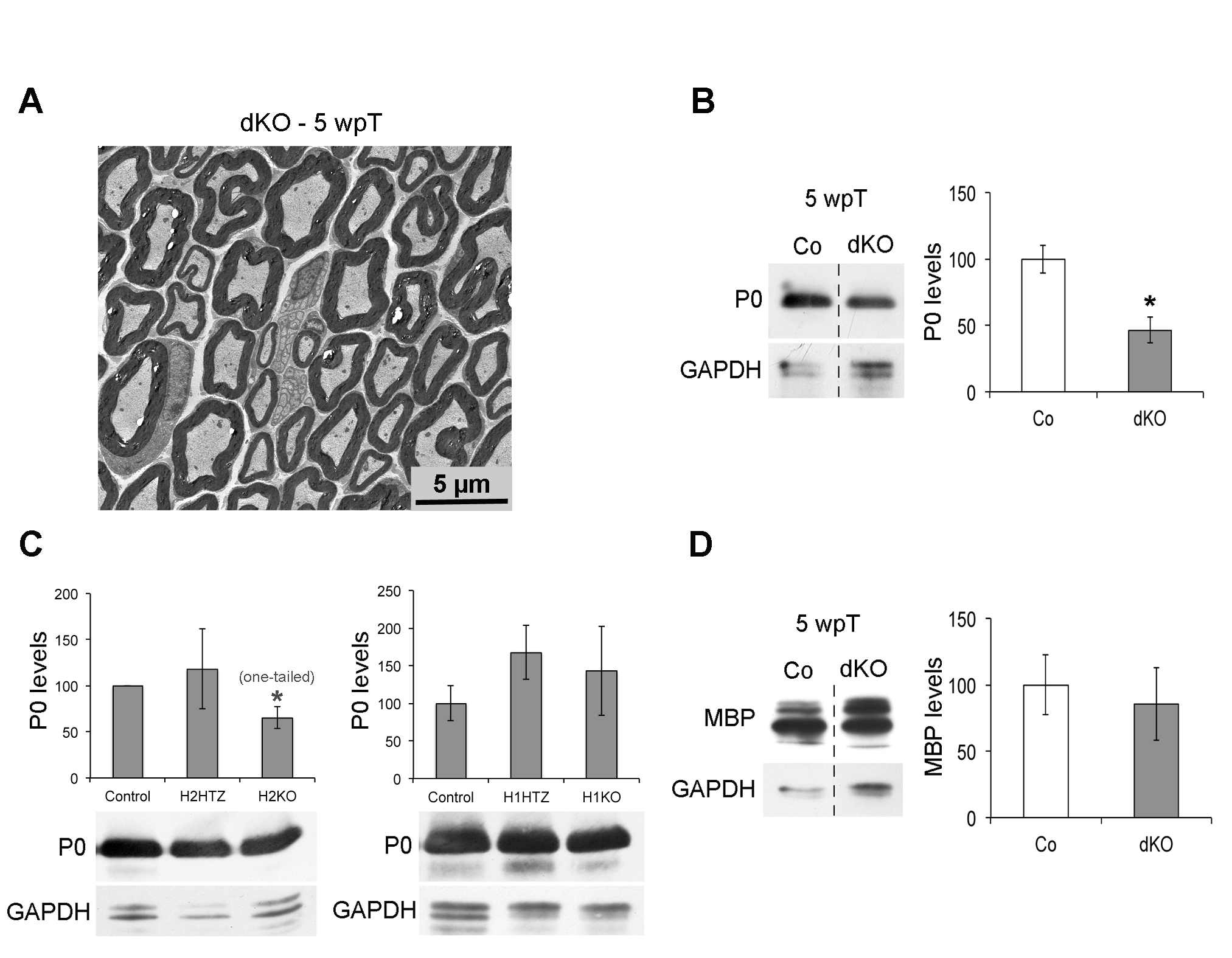

Supplement: S2 Fig — (A) Electron micrograph of ultrathin cross sections of dKO sciatic nerve at 5 wk post-tamoxifen. Sciatic nerves of 3 dKO mice were analyzed and no demyelinated or remyelinated axon or macrophage were found. (B–D) Western blot of P0 (B,C) and MBP (D) in control and dKO sciatic nerve lysates at 5 wk post-tamoxifen (B,D), and in control, H1HTZ, H1KO, H2HTZ, and H2KO at 8 wk post-tamoxifen (C), and quantification of protein levels normalized to the loading control GAPDH in mutants compared to controls (= 100%) (3 animals per genotype were used). In (B,D), the dashed lines indicate that lysates were run on the same gel but not on consecutive lanes. P-values (unpaired (B,C,D) or paired (C, HDAC2 single mutants) two-tailed (unless stated otherwise in the figure) Student's t test): * = p < 0.05, error bars = SEM. (TIF) [file pbio.1002258.s002.tif]

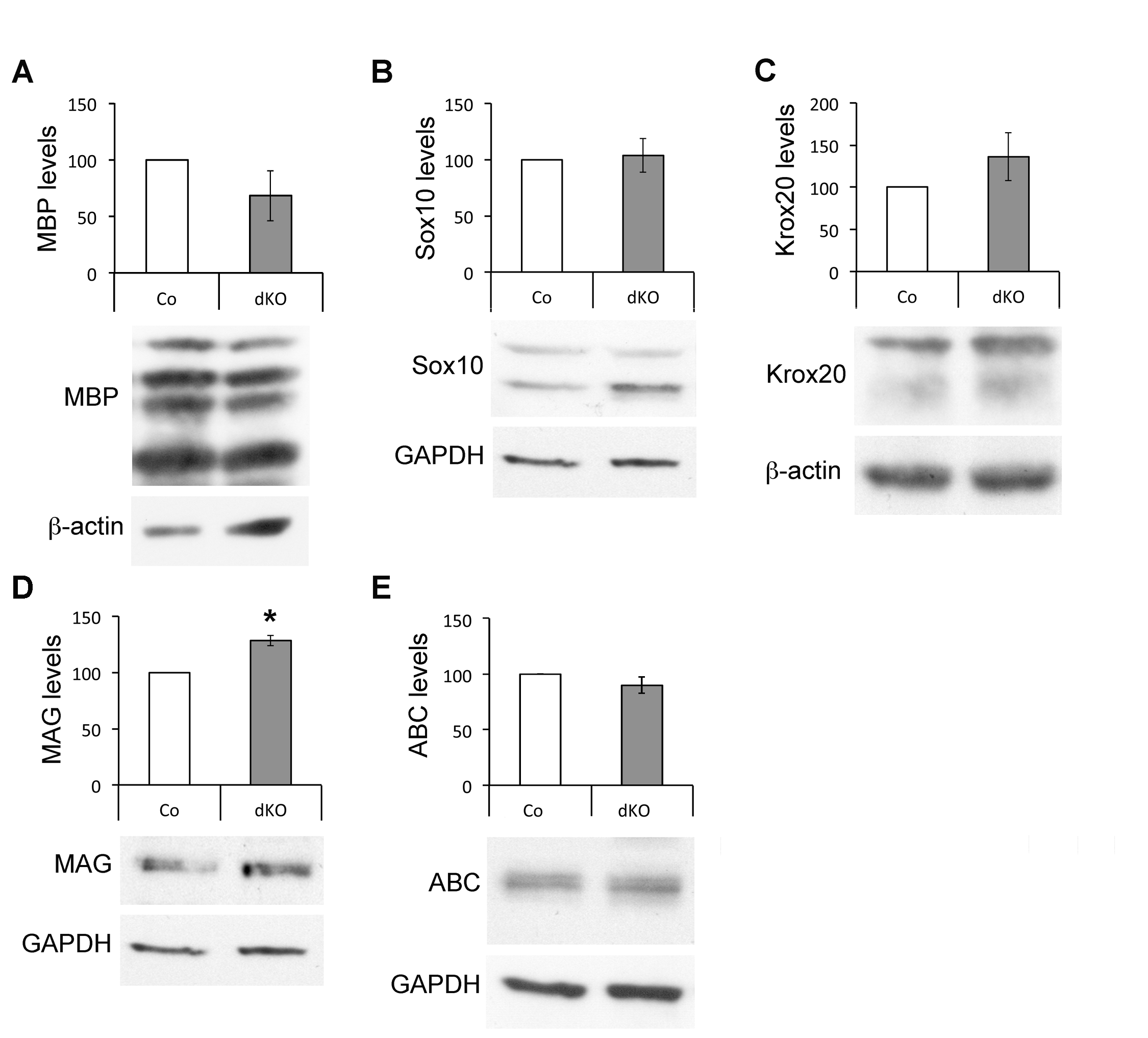

Supplement: S3 Fig — Western blots of MBP (A), Sox10 (B), Krox20 (C), MAG (D), and ABC (E) in lysates of control (Co) and dKO sciatic nerves at 8 wk post-tamoxifen, and quantification of protein levels normalized to GAPDH or beta-actin loading control in dKOs compared to controls (= 100%). For each experiment, three control and three dKO animals were used. P-values (paired two-tailed Student's t test): * = p < 0.05, error bars = SEM. (TIF) [file pbio.1002258.s003.tif]

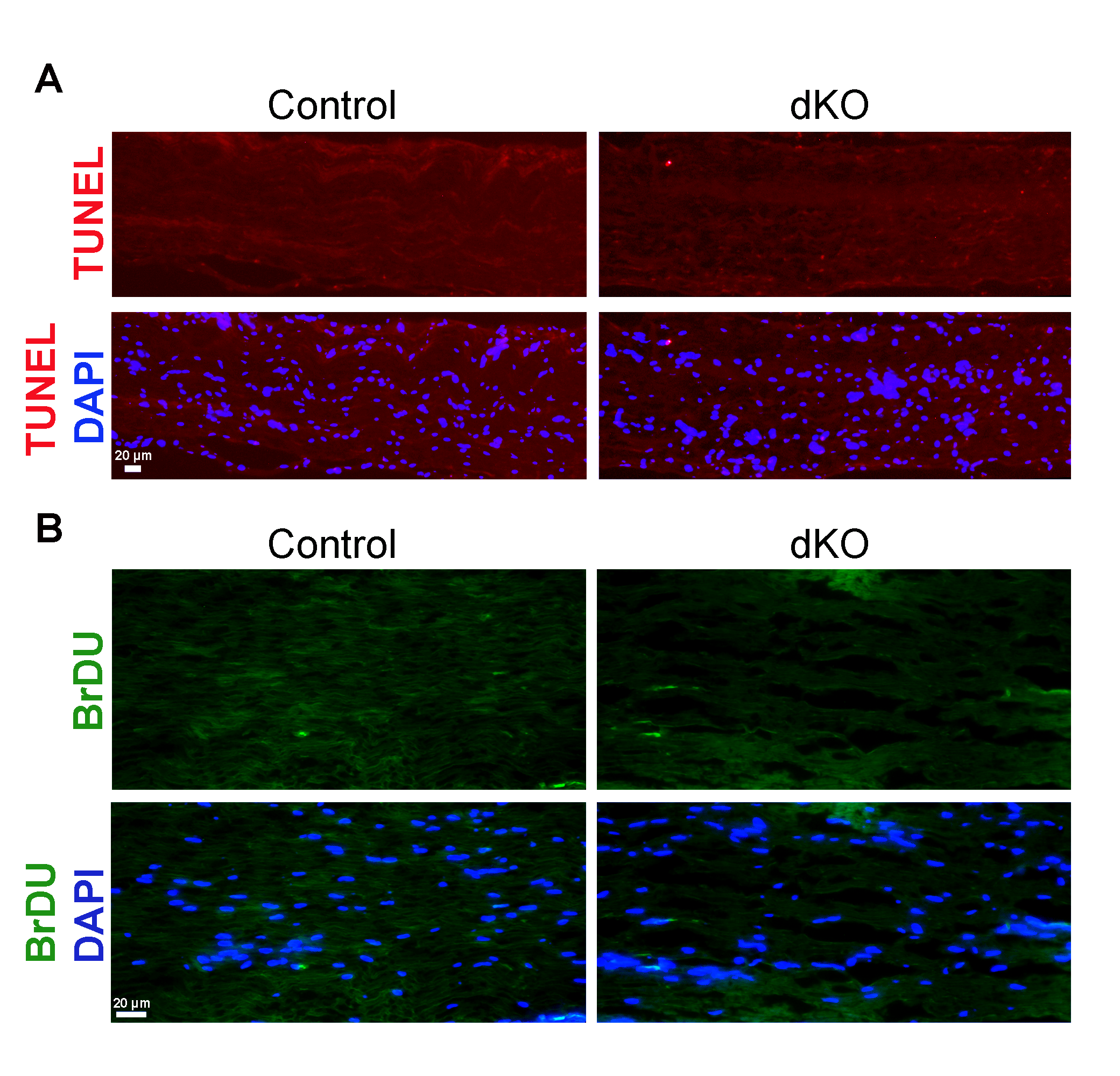

Supplement: S4 Fig — TUNEL assay (red, A) and BrdU assay (green, B) to detect apoptotic and proliferating cells, respectively, in longitudinal cryosections of control and dKO sciatic nerves at 8 wk (A) and 5 wk (B) post-tamoxifen. Nuclei are labeled in blue with DAPI. (TIF) [file pbio.1002258.s004.tif]

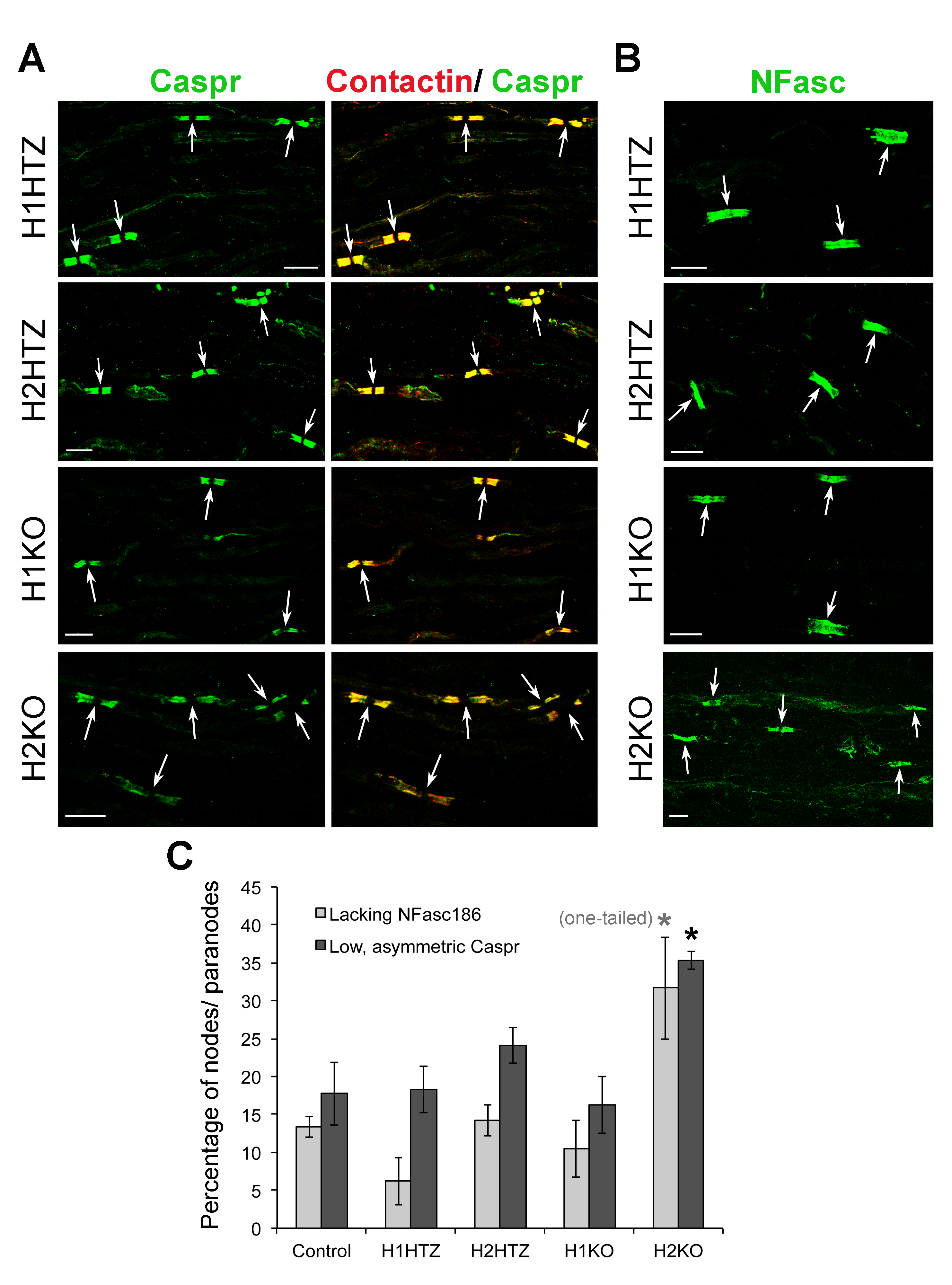

Supplement: S5 Fig — (A) Coimmunofluorescence of Caspr (green) and Contactin (red) and (B) immunofluorescence of total neurofascins (NFasc, green) in longitudinal cryosections of control and dKO sciatic nerves at 8 wk post-tamoxifen, and (C) quantification of nodes lacking NFasc186 and paranodes with low and/or asymmetric Caspr (3 animals per genotype were quantified, at least 50 nodes/paranodes per animal and 150 per genotype). P-values (unpaired two-tailed (unless stated otherwise in the figure) Student's t test): * = p < 0.05, error bars = SEM. Arrows indicate nodes of Ranvier. Scale bars = 5 μm. (TIF) [file pbio.1002258.s005.tif]

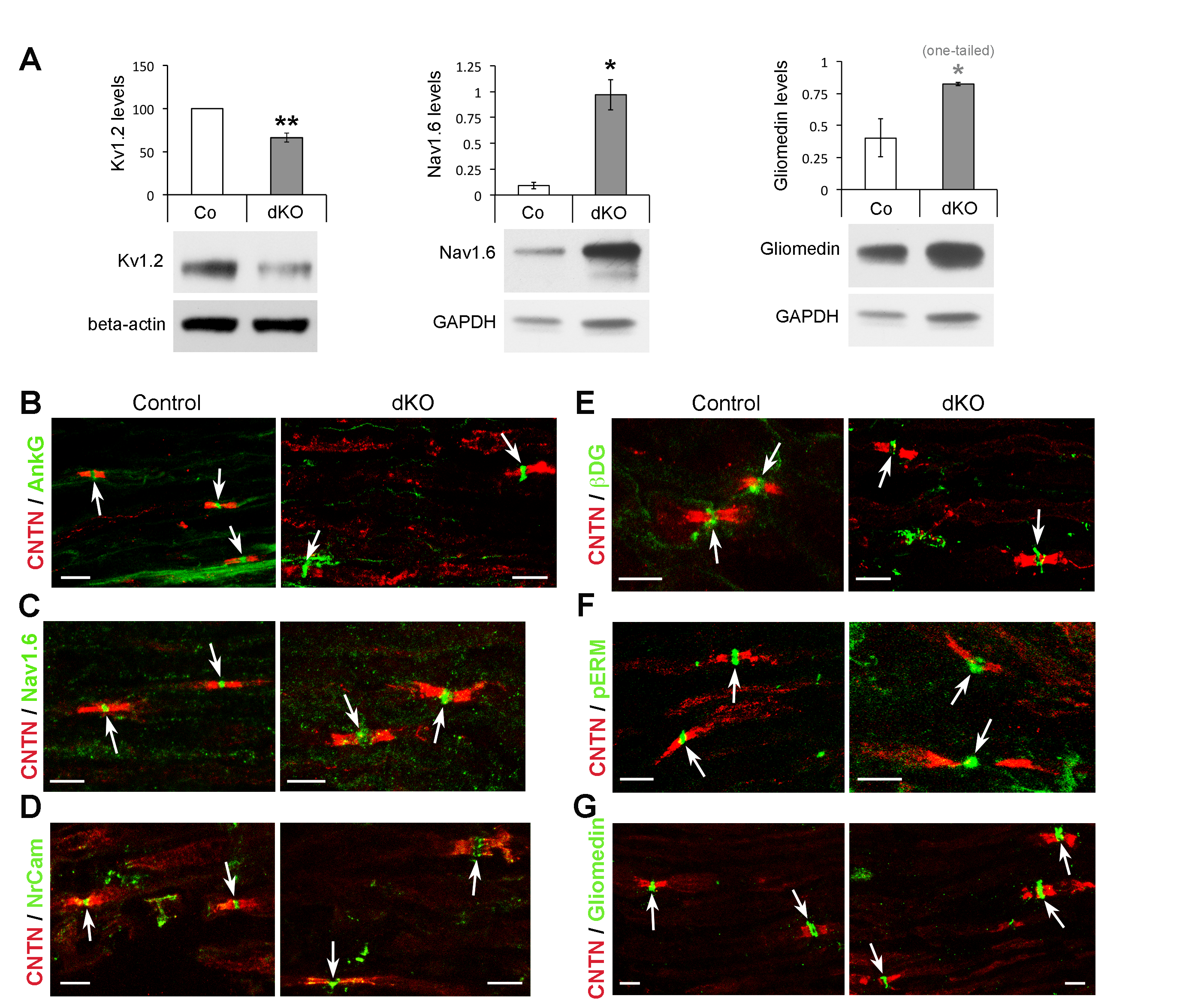

Supplement: S6 Fig — (A) Western blots of Kv1.2, Nav1.6, and Gliomedin, and quantification normalized to the loading control beta-actin or GAPDH. Results are presented as percentage of the control (= 100%) for Kv1.2 or as ratios to GAPDH for Nav1.6 and Gliomedin. For each experiment, 3 control and 3 dKO animals were used. P-values (paired (for Kv1.2) or unpaired two-tailed (unless stated otherwise in the figure) Student's t test): * = p < 0.05, ** = p < 0.01, error bars = SEM. (B–G) Coimmuofluorescence of Contactin (CNTN, red) with (B) Ankyrin G (AnkG, green), (C) Nav1.6 (green), (D) NrCam (green), (E) beta-dystroglycan (βDG, green), (F) phospho-Ezrin-Radixin-Moesin (pERM, green), or Gliomedin (green) on longitudinal cryosections of control and dKO sciatic nerves at 8 wk post-tamoxifen. Z-series projections of confocal images are shown. Arrows show the position of nodes of Ranvier. Scale bars = 5 μm. (TIF) [file pbio.1002258.s006.tif]

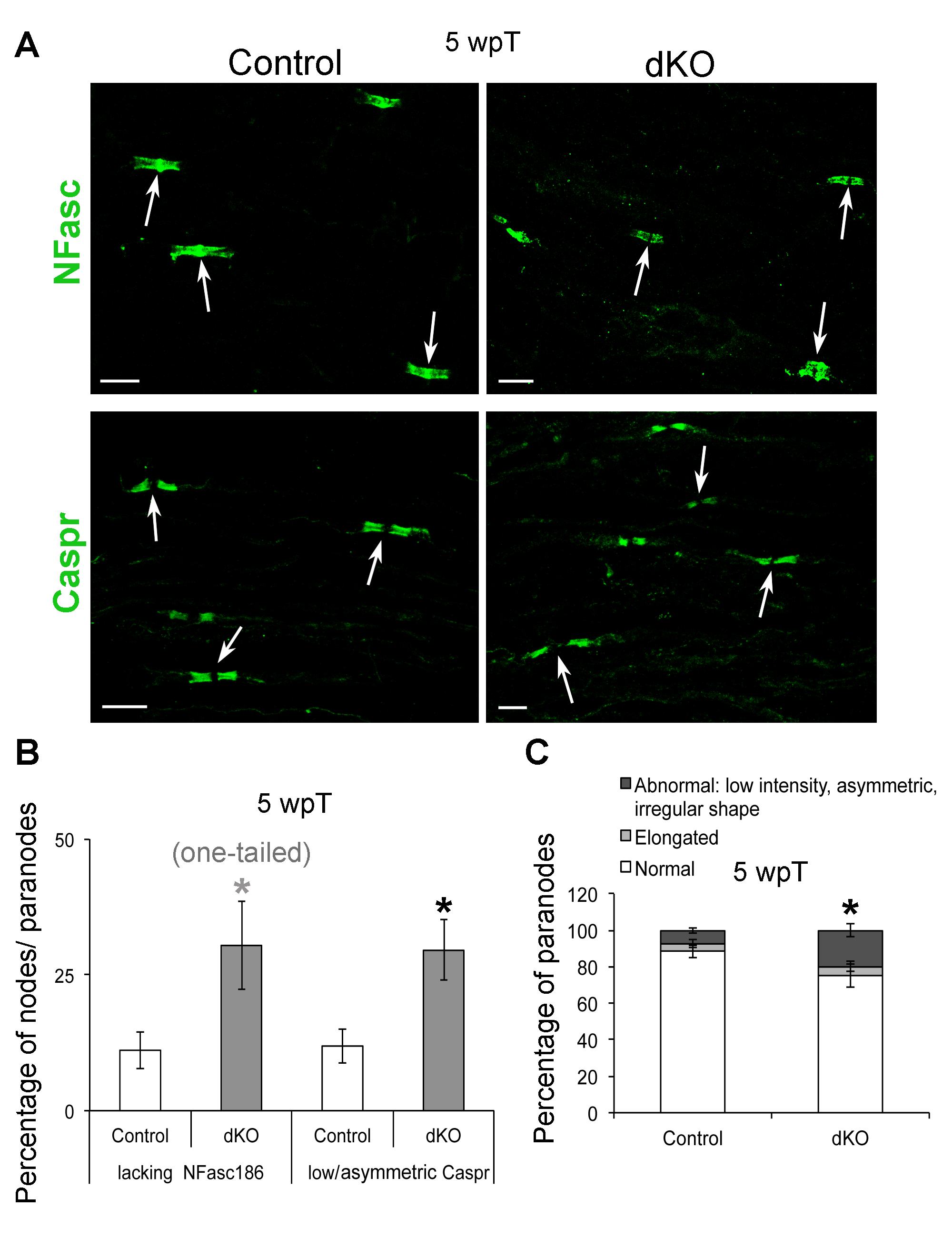

Supplement: S7 Fig — (A) Immunofluorescence of neurofascins (green) or Caspr (green) on longitudinal cryosections of control and dKO sciatic nerves at 5 wk post-tamoxifen. Z-series projections of confocal stacks are shown. Arrows show the position of nodes of Ranvier. Scale bars = 5 μm. (B) Quantification of nodes lacking NFasc186 and paranodes with low or asymmetric Caspr. (C) Quantification of normal, elongated, and abnormal (low intensity, asymmetric, irregular shape) paranodes, based on NFasc staining in paranodes. Three animals per genotype were used, 100–200 nodes and paranodes counted per animal, 350 to 500 counted per genotype. P-values (unpaired two-tailed (unless stated otherwise in the figure) Student's t test): * = p < 0.05, error bars = SEM. (TIF) [file pbio.1002258.s007.tif]

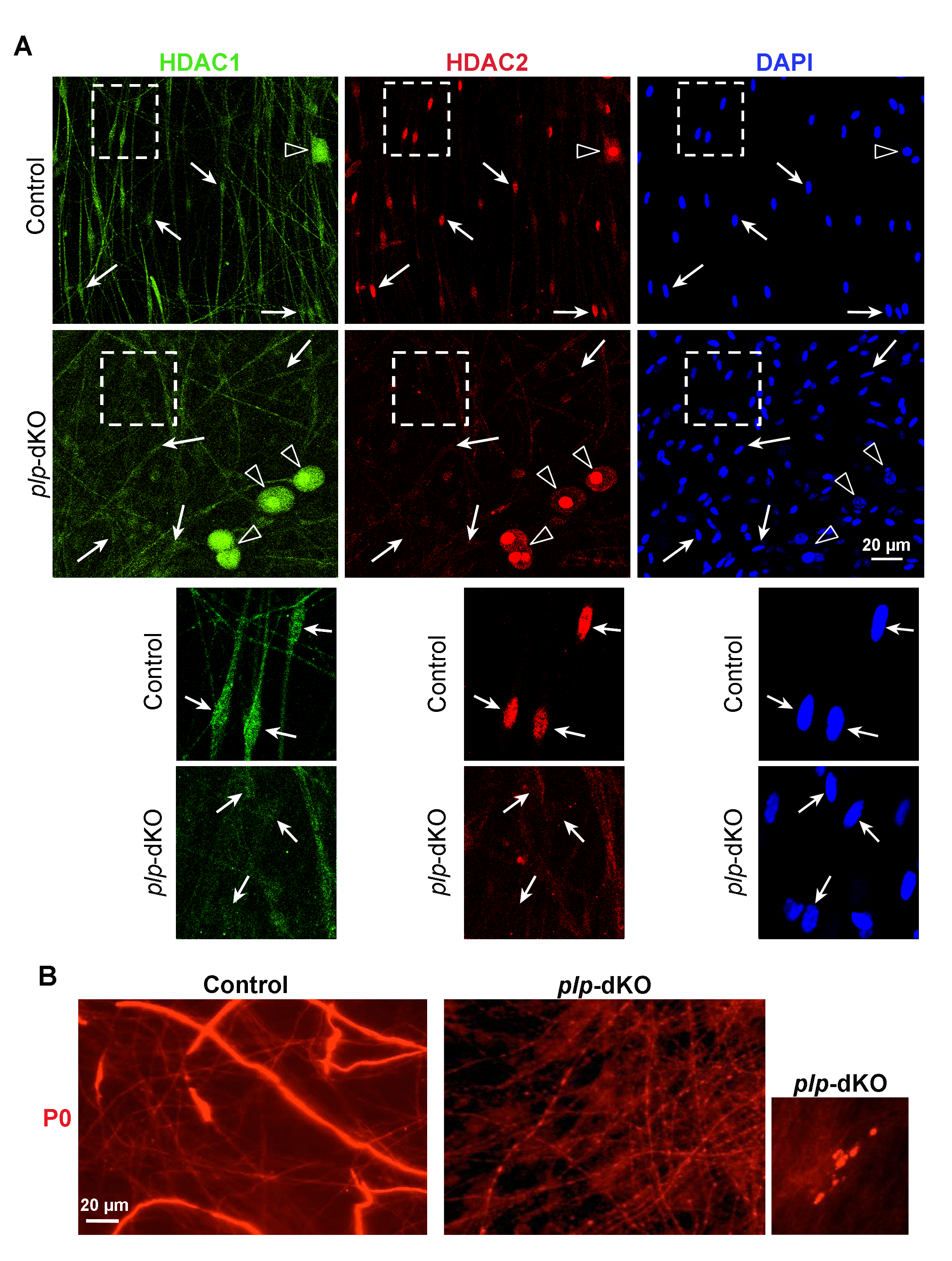

Supplement: S8 Fig — (A) HDAC1 (green) and HDAC2 (red) coimmunofluorescence in myelinated DRG cultures of control and plp-dKO embryos at 8 d post-tamoxifen treatment. Note that neuron nuclei (round nuclei marked by open arrowheads) express high levels of HDAC1 and HDAC2 compared to Schwann cell nuclei (small elongated nuclei marked by arrows). Smaller images are magnifications of the white boxes depicted in the larger images. Nuclei are labeled in blue with DAPI. (B) Photographs of P0 immunofluorescence in myelinated control and plp-dKO DRG cultures 10 d post-tamoxifen treatment. The punctuated P0 signal in plp-dKO DRG suggests myelin breakdown. DRG of at least three control and three plp-dKO embryos were analyzed, and representative pictures are shown. (TIF) [file pbio.1002258.s008.tif]

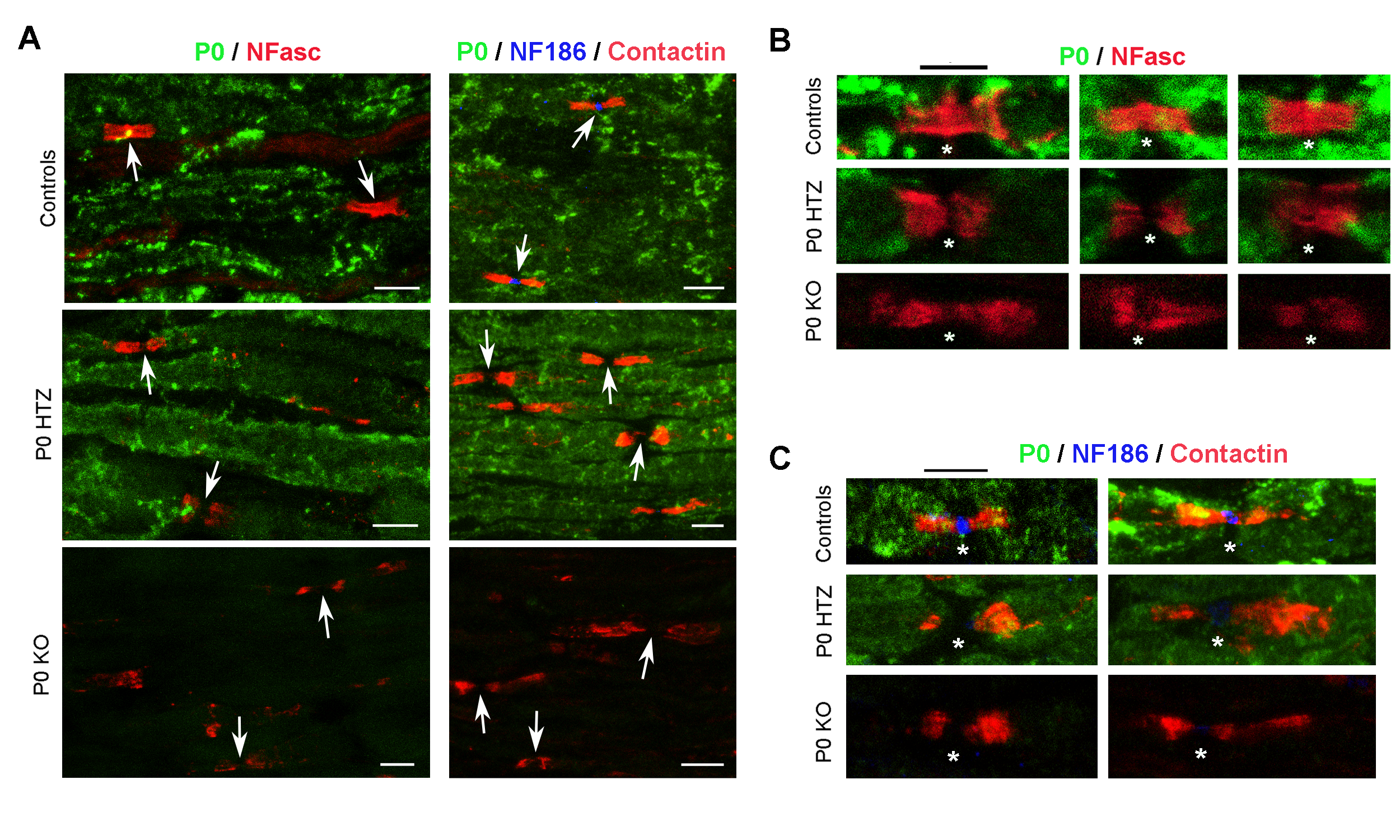

Supplement: S9 Fig — Coimmunofluorescence of total neurofascins (NFasc, red) with P0 (green) (A,B) or NFasc186 (blue), Contactin (red) and P0 (green) (A,C) on longitudinal cryosections of control, P0 HTZ and P0 KO adult (10-month old) sciatic nerves. Representative photographs of 2 control, 4 P0 HTZ, and 2 P0 KO mice are shown. Asterisks mark the position (lateral dimension) of the node of Ranvier. In (B,C), magnifications of single paranodal/nodal regions are shown. Scale bars = 5 μm. (TIF) [file pbio.1002258.s009.tif]

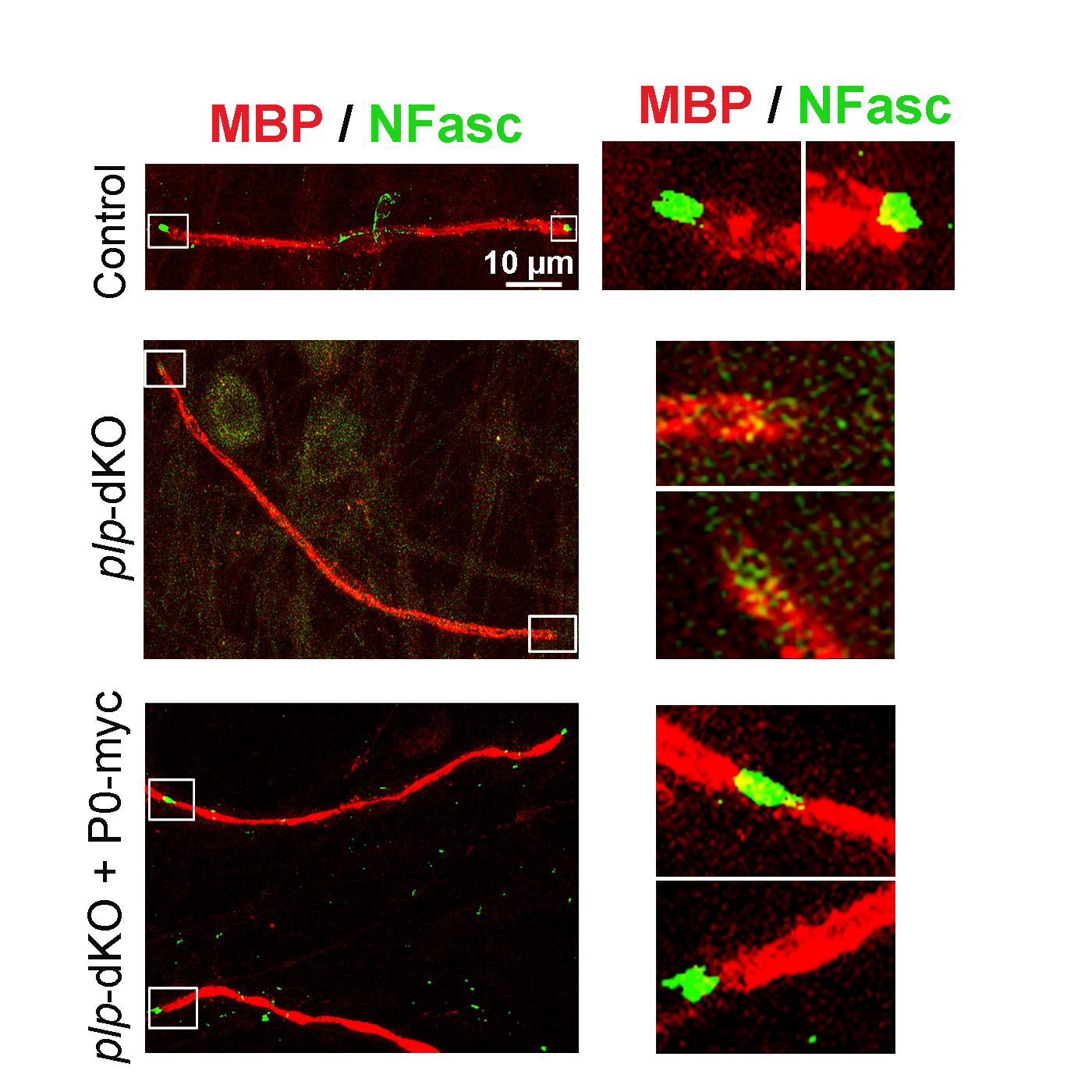

Supplement: S10 Fig — Coimmunofluorescence of total neurofascins (NFasc, green) and MBP (red) in control or plp-dKO myelinated DRG cultures transduced with lentiviruses expressing GFP (Control and plp-dKO) or P0-myc (plp-dKO + P0-myc). Z-series projections of confocal stacks are shown. Images on the right are magnifications of white boxes depicted on the left images highlighting heminodes or nodes. DRG of three control and three plp-dKO embryos were analyzed and representative images are shown. (TIF) [file pbio.1002258.s010.tif]

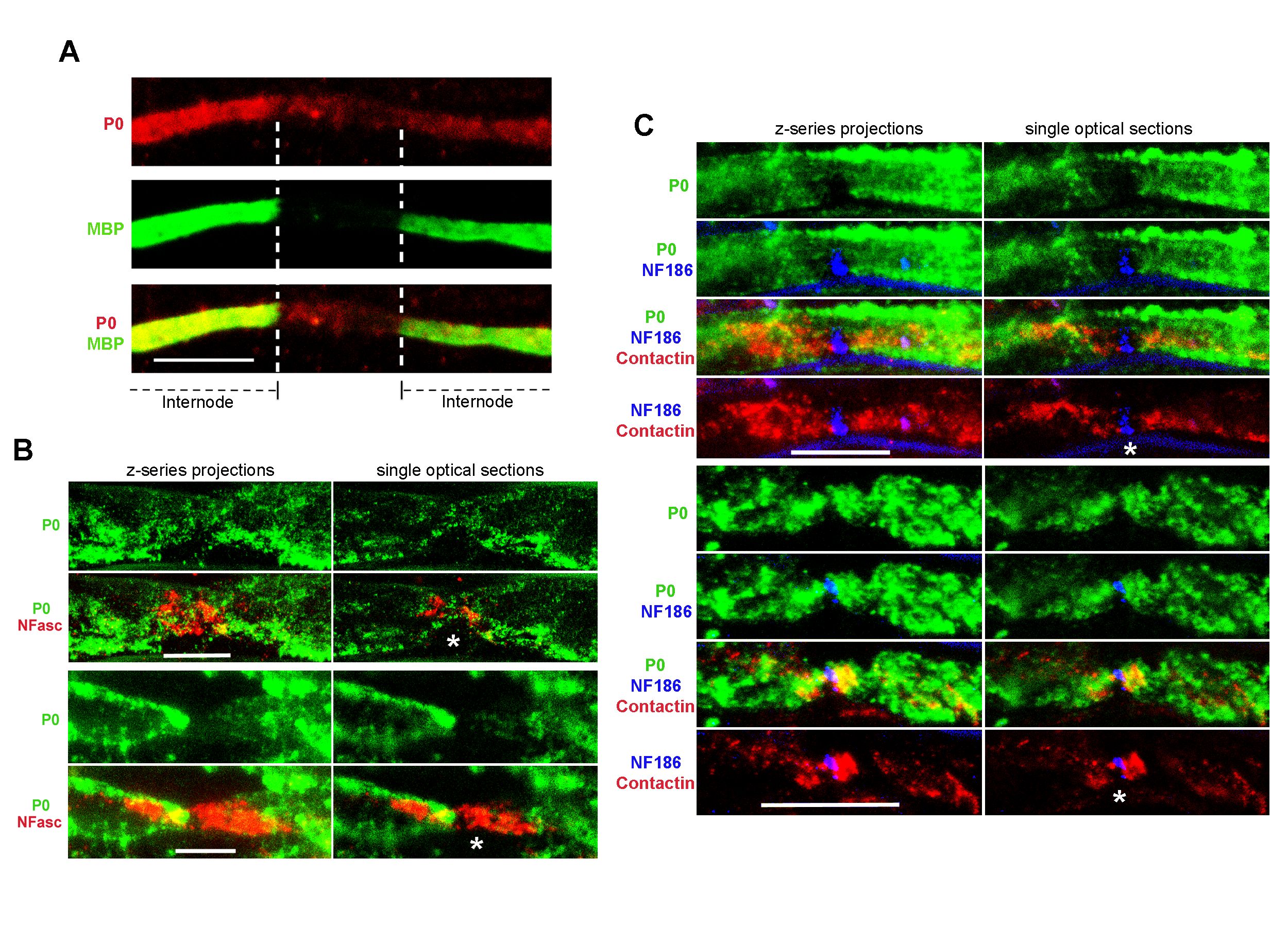

Supplement: S11 Fig — (A) Confocal images (z-series projections) of P0 (red) and MBP (green) coimmunofluorescence in myelinated mouse DRG cultures. Overlay appears yellow. DRG cultures of six control embryos were analyzed, and a representative P0-positive fiber is shown. White dashed lines delineate MBP signal that is apparently restricted to internodes, whereas P0 signal extends further between two internodes. Scale bar = 5 μm. (B–C) Coimmunofluorescence of total neurofascins (NFasc, red) and P0 (green) (B) or of NFasc186 (blue), Contactin (red), and P0 (green) (C) on longitudinal cryosections of adult human peripheral nerves. Median, tibial, and/or sciatic nerves of three human individuals were analyzed and representative pictures of sciatic nerves are shown. P0 was abundant in all paranodal/nodal regions we observed. Z-series projections and single optical sections are shown. Asterisks mark the position (lateral dimension) of the node of Ranvier. Scale bars = 5 μm in (B) and 10 μm in (C). (TIF) [file pbio.1002258.s011.tif]

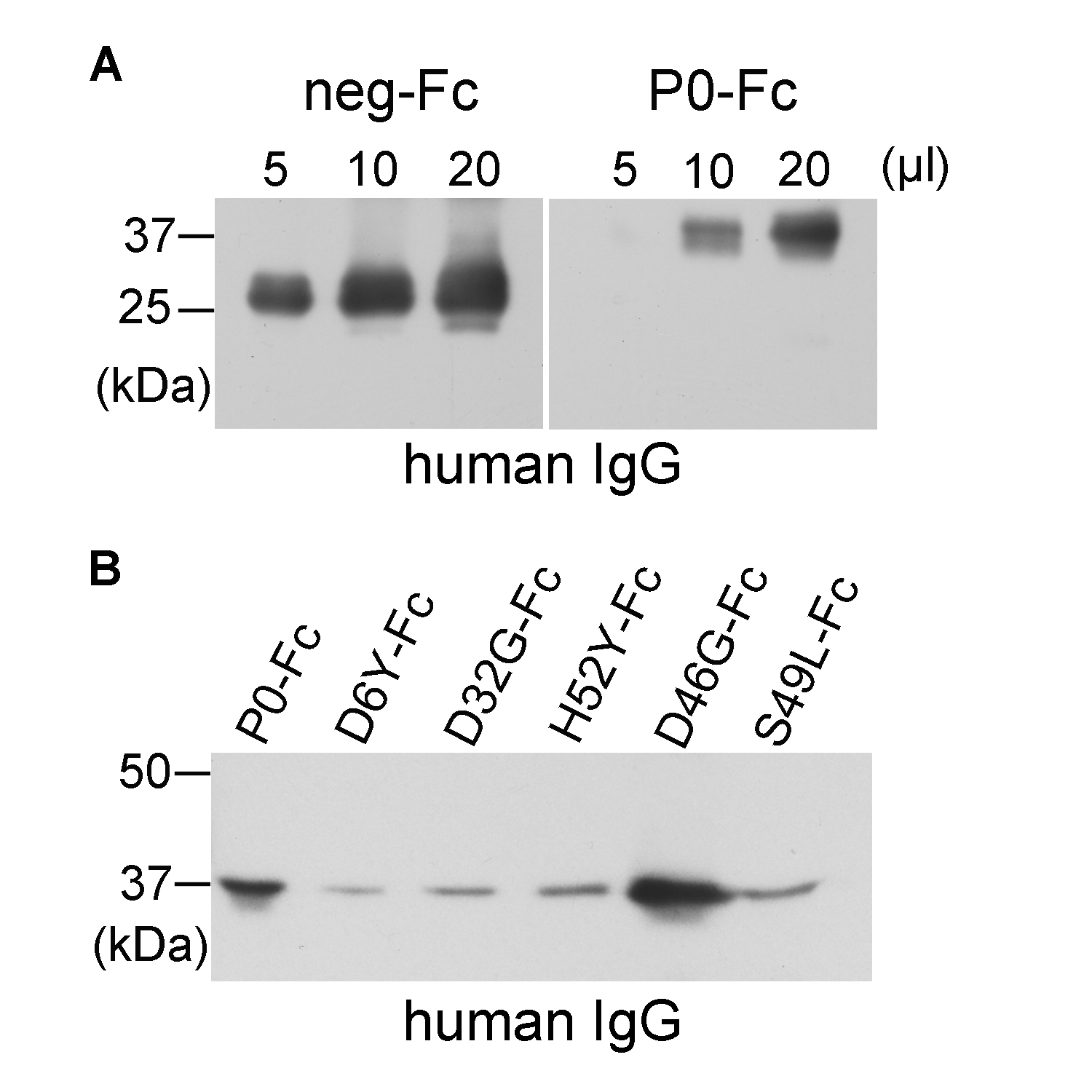

Supplement: S12 Fig — Western blot of purified control Fc (neg-Fc) and P0-Fc particles (A) and of P0-Fc, P0-D6Y-Fc, P0-D32G-Fc, P0-H52Y-Fc, P0-D46G-Fc (silent mutation for binding to P0 and neurofascins), and P0-S49L-Fc particles (B) with antihuman IgG antibody coupled to HRP. Increasing amounts of purified neg-Fc and P0-Fc are loaded in (A) and 10 μl of each P0-Fc wild type or mutant are loaded in (B). The expected size of neg-Fc is 25.5 kDa, while the expected size of P0-Fc is around 38 kDa. (TIF) [file pbio.1002258.s012.tif]

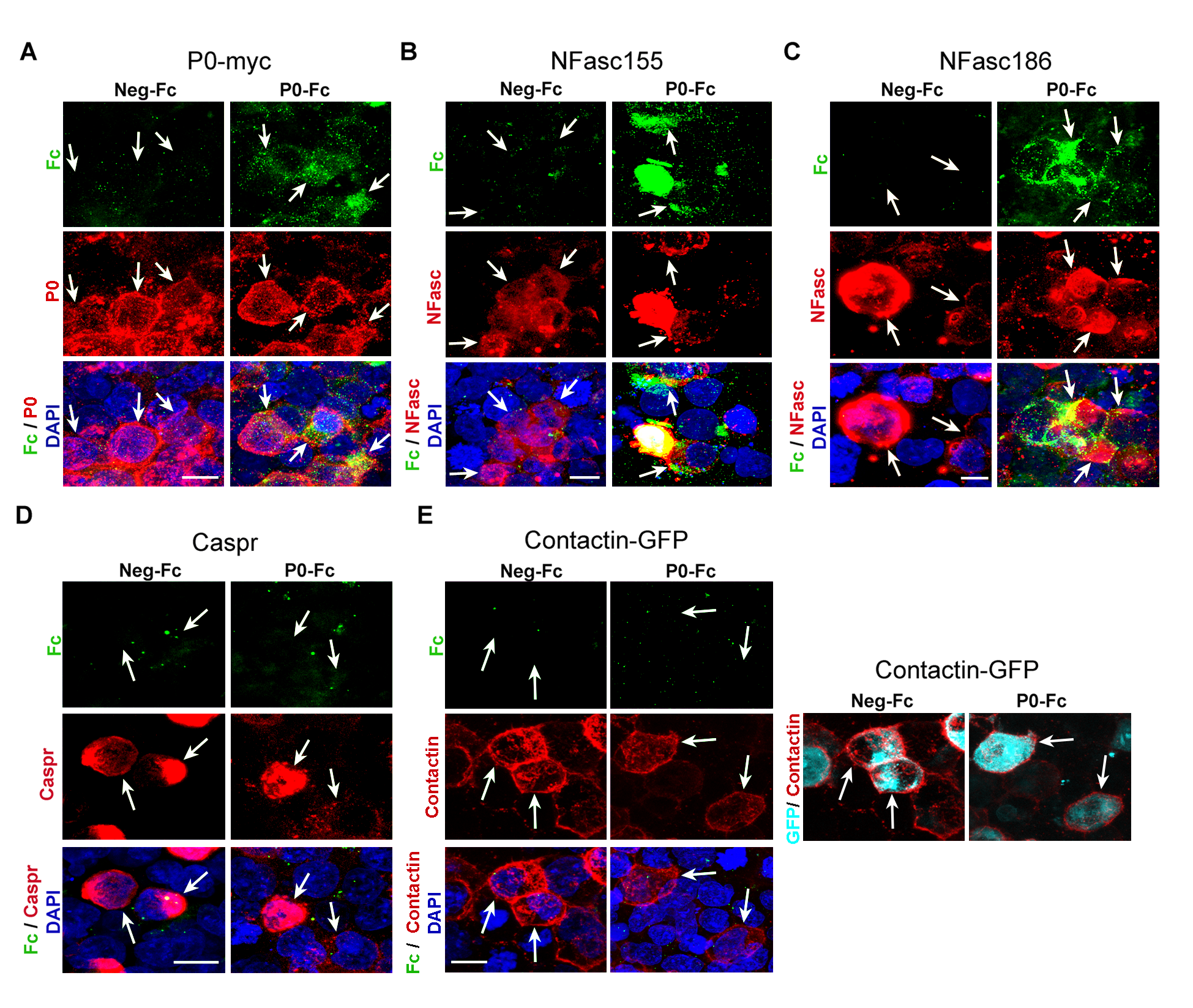

Supplement: S13 Fig — (A–E) Adhesion assay in HEK293T cells. Confocal images of P0-Fc or control-Fc (Neg-Fc) particles (green, or false-colored green for Contactin-GFP) and P0 (A, red), neurofascins (B and C, red), Caspr (D, red), Contactin (E, red). or GFP (E, false-colored turquoise) coimmunofluorescence in HEK293T cells expressing P0-myc (A), NFasc155 (B), NFasc186 (C), Caspr (D), or Contactin-GFP (E). Overlays appear yellow. Nuclei are labeled in blue with DAPI. All labelings were carried out on unpermeabilized cells, and z-series projections are shown. Each experiment was done at least three times, and representative pictures are shown. Scale bars = 5 μm. (TIF) [file pbio.1002258.s013.tif]

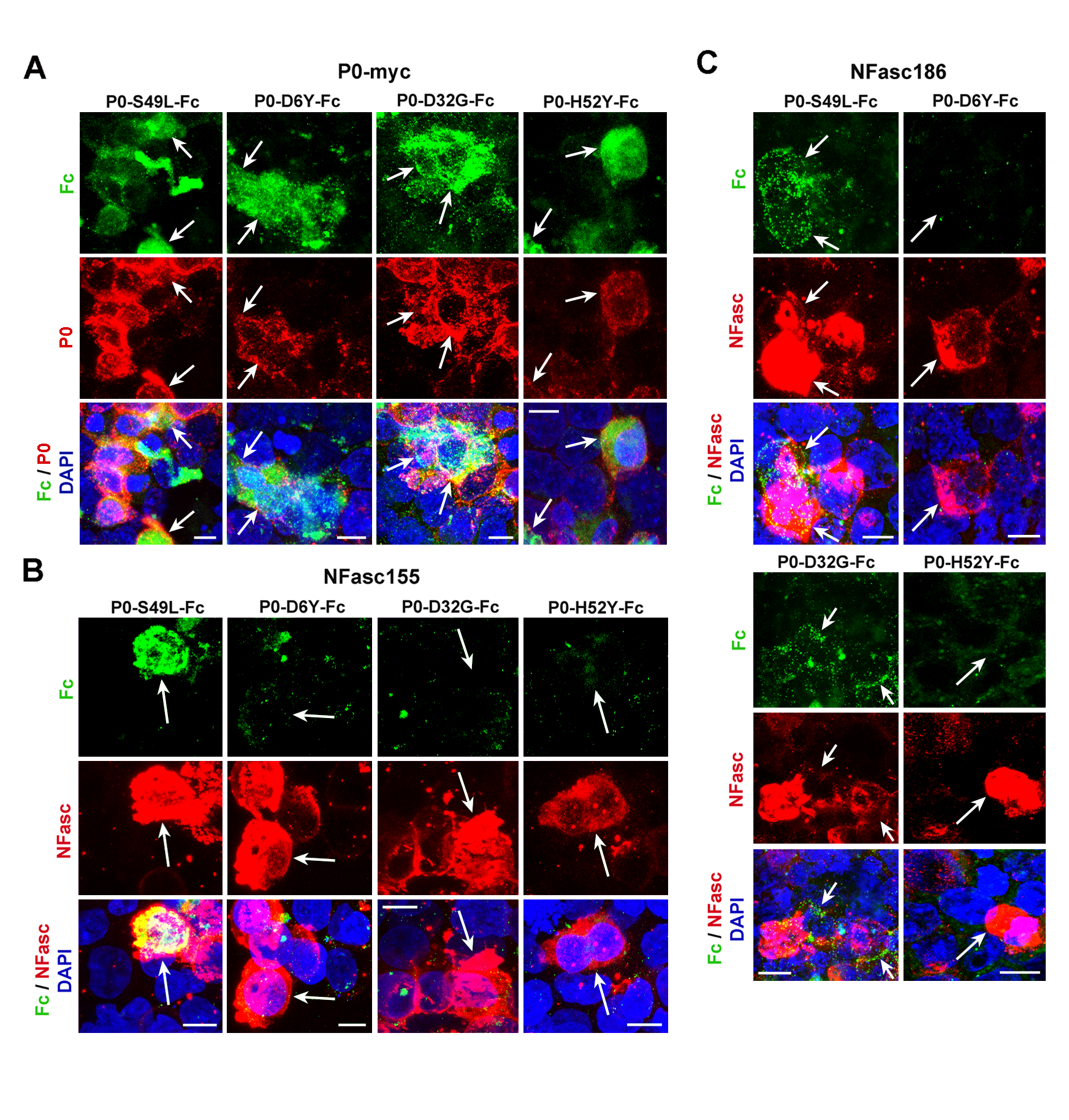

Supplement: S14 Fig — Adhesion assay in HEK293T cells. Confocal images of P0-D6Y-Fc, P0-D32G-Fc, P0-H52Y-Fc, or P0-S49L-Fc particles (green) and neurofascins or P0 (red) coimmunofluorescence in HEK293T cells expressing P0-myc (A), NFasc155 (B), or NFasc186 (C). Overlays appear yellow. Nuclei are labeled in blue with DAPI. All labelings were carried out on unpermeabilized cells, and z-series projections are shown. At least three independent experiments were analyzed for each panel, and representative pictures are shown. Scale bars = 5 μm. (TIF) [file pbio.1002258.s014.tif]

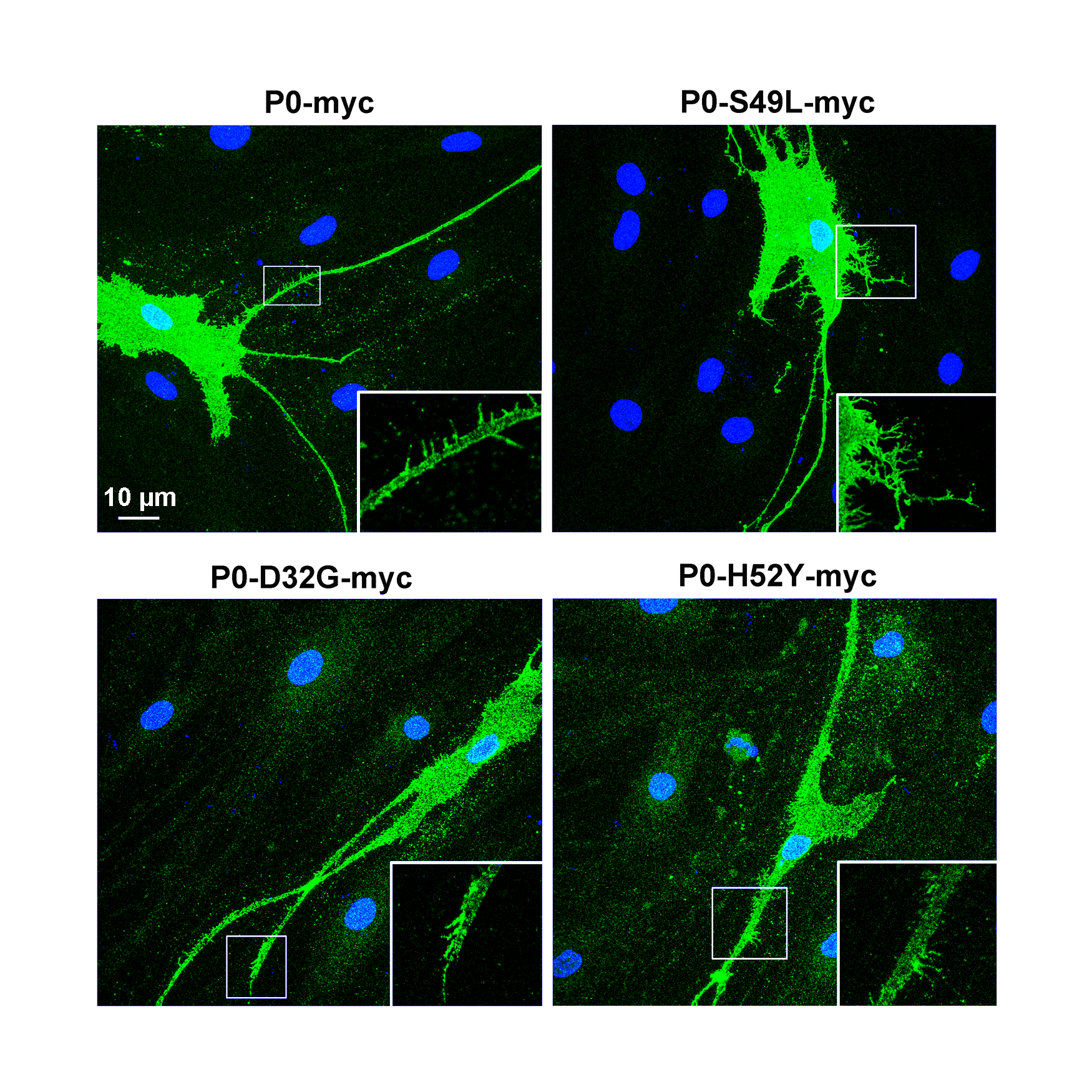

Supplement: S15 Fig — Immunofluorescence of Myc (green) counterstained with DAPI (blue) to detect myc-tagged wild-type P0 (P0-myc), D32G, H52Y, and S49L P0 mutants in primary rat Schwann cells transfected with either pTK-P0-myc, pTK-D32G-myc, pTK-H52Y-myc, or pTK-S49L-myc and cultured in differentiating conditions for 3 d. All Myc positive cells (~50 cells per construct) showed plasma membrane localization of wild-type P0-myc and mutants. (TIF) [file pbio.1002258.s015.tif]

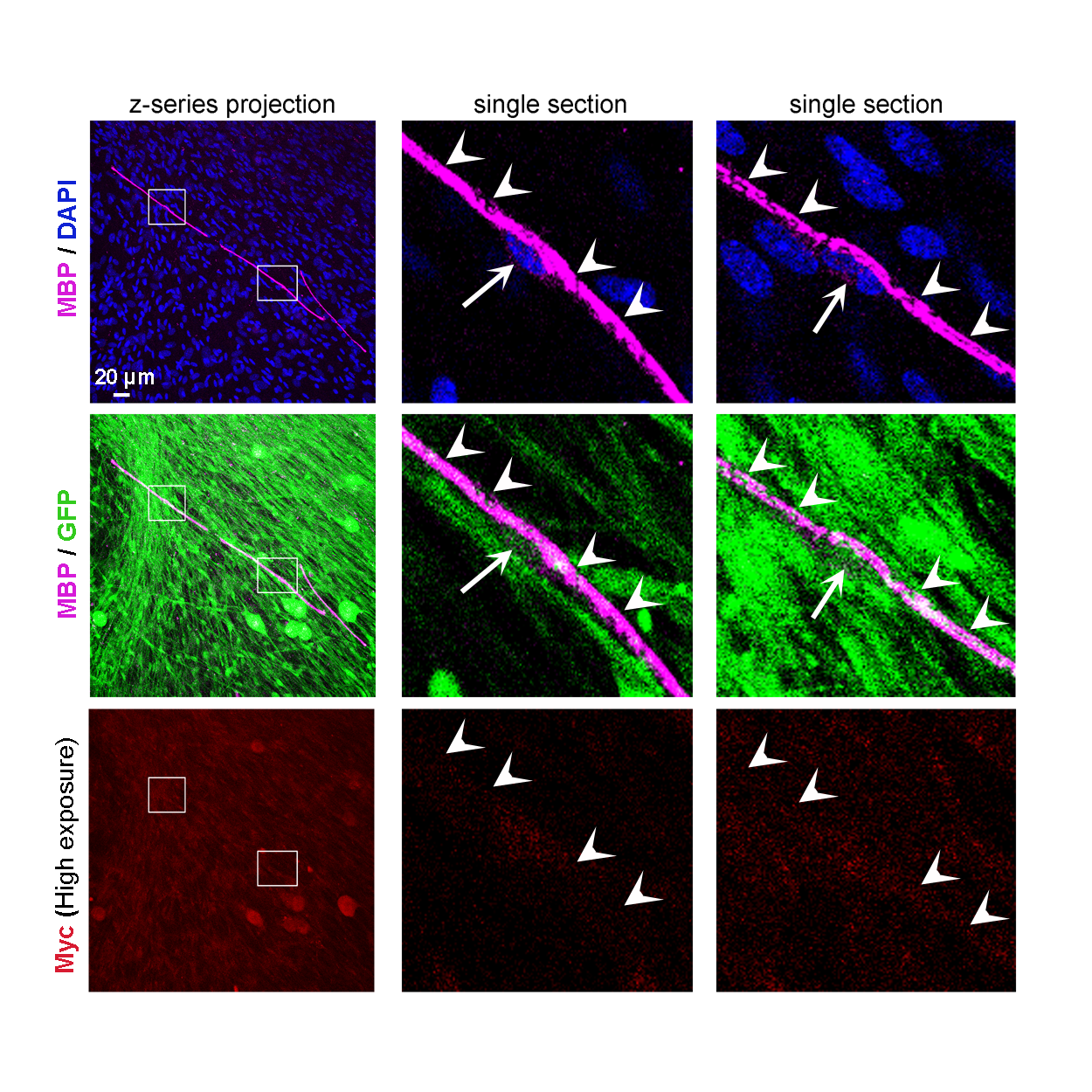

Supplement: S16 Fig — Coimmunofluorescence of MBP (Magenta, rat antibody) and Myc (red, mouse antibody), and GFP fluorescence (green) in myelinated plp-dKO DRG cultures transduced with lentiviruses expressing GFP and treated with tamoxifen for 10 d. Even at high exposure, Myc staining did not cross-react with MBP staining. To avoid cross-reactivity, we used multiple labeling (adsorbed against many animal species, including rat for antimouse and mouse for antirat) secondary antimouse and antirat antibodies. Antibody concentrations and staining protocol (buffers, incubation times and temperature, washes) were the same as for stainings presented in Fig 9A. Nuclei are labeled in blue with DAPI. Pictures on the right (single optical sections) are magnifications of the white boxes depicted on left images (z-series projections). Arrows indicate Schwann cell nuclei of myelinated fibers, arrowheads indicate MBP staining. DRG of three plp-dKO embryos were analyzed. None of the MBP-positive fibers were labeled by Myc staining. (TIF) [file pbio.1002258.s016.tif]

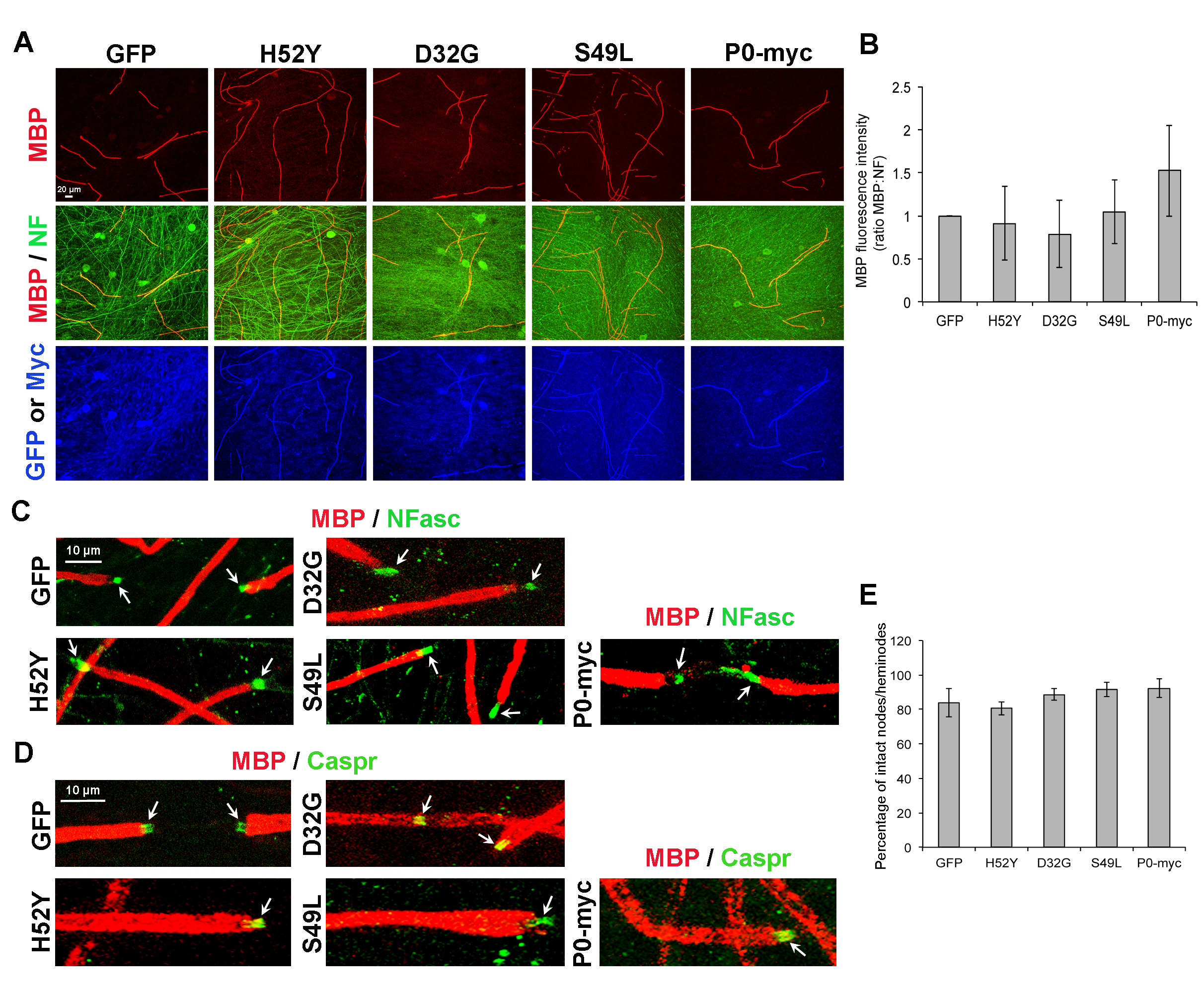

Supplement: S17 Fig — Coimmunofluorescence of MBP (red) and (A) neurofilament (NF, green) and Myc or GFP fluorescence (blue), or (C) neurofascins (NFasc, green), or (D) Caspr (green) in myelinated HDAC1/2 control DRG cultures transduced with lentiviruses expressing either GFP, H52Y-myc, D32G-myc, S49L-myc, or P0-myc, and treated with tamoxifen for 10 d after completion of myelination. Arrows indicate paranodes/nodes. In (B), quantification of MBP fluorescence intensity normalized to NF and compared to GFP (set to 1). DRG of three control embryos were quantified, four coverslips per embryo were analyzed, and representative pictures are shown. In (E), the graph represents the percentage of intact (high NFasc levels) nodes and heminodes. DRG of three control embryos were quantified, four coverslips per control, 40 to 80 nodes/heminodes counted per control per virus. Error bars = SEM. (TIF) [file pbio.1002258.s017.tif]

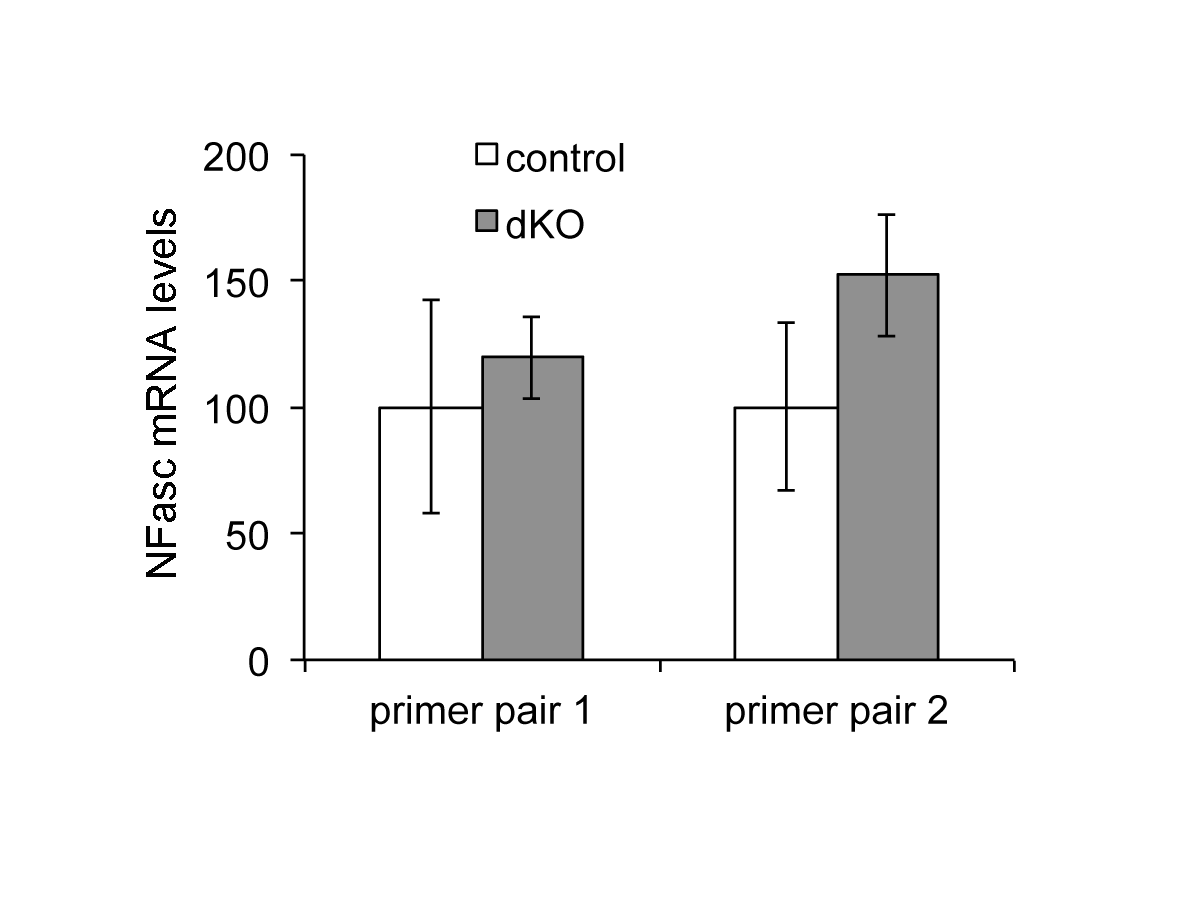

Supplement: S18 Fig — Graph showing mRNA levels of NFasc155 (primer pair 1) and NFasc (presumably also NFasc155, primer pair 2) normalized to GAPDH and measured by real-time qPCR with two different primer pairs in dKO compared to control (= 100%) sciatic nerves at 5 wk post-tamoxifen, before the influx of macrophages, but when P0 protein levels were already reduced (see S2B Fig) and the localization of NFasc at the nodes/paranodes was already significantly affected (see S7 Fig) in dKO sciatic nerves. Sciatic nerves of three dKO and three control littermate animals were used, and no significant difference was detected. (TIF) [file pbio.1002258.s018.tif]

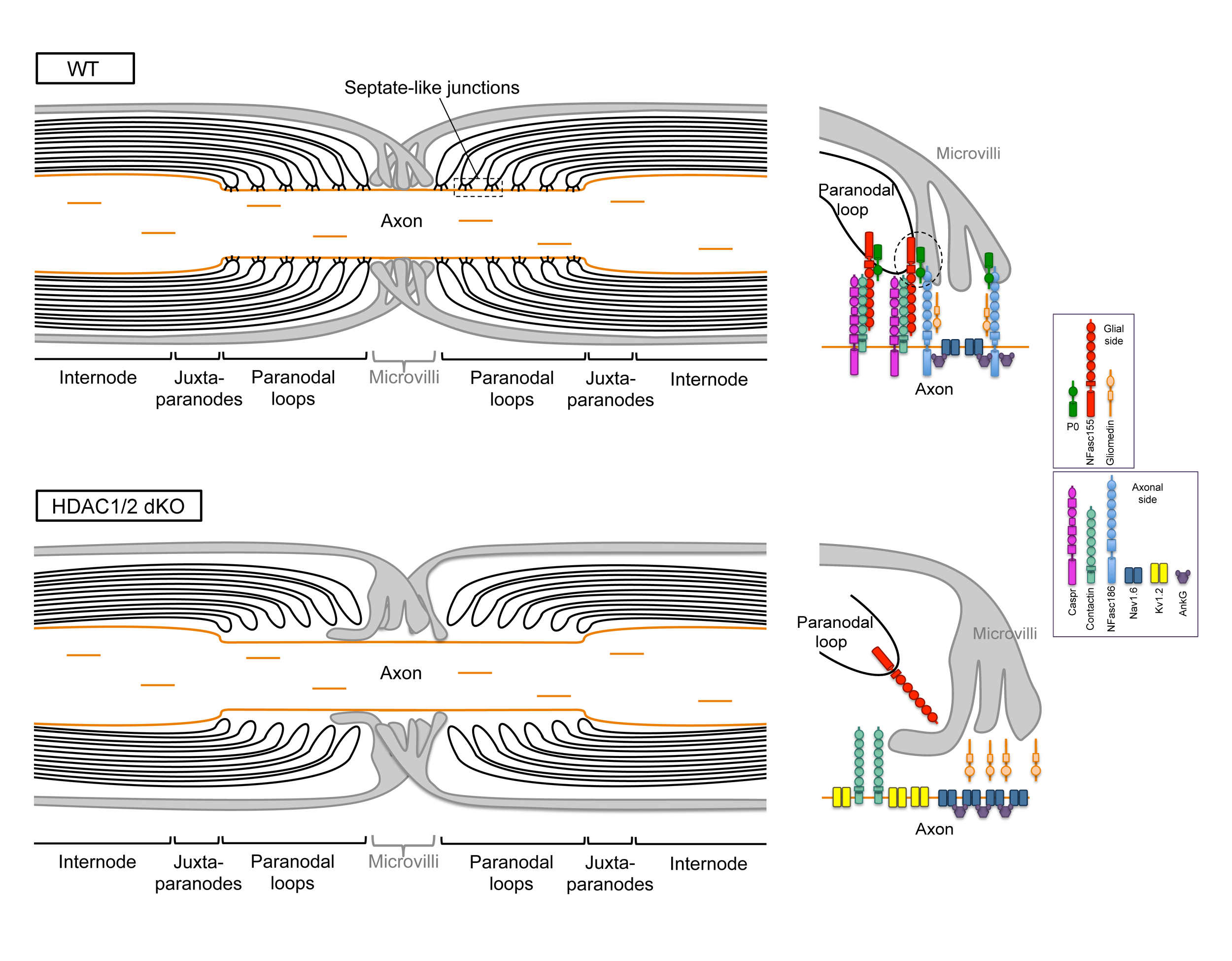

Supplement: S19 Fig — P0 interacts with NFasc155 in paranodes and with NFasc186 in nodes of Ranvier. The dashed circle around NFasc155, P0, and NFasc186 indicates the hypothetical simultaneous interaction of P0 with both NFasc155 and NFasc186. Ablation of HDAC1/2 in adult SCs leads to loss of P0, which results in 1) mislocalization and decrease of NFasc155 levels, loss of Caspr and septate-like junctions, and mislocalization of Kv1.2 in paranodes, and 2) loss of NFasc186 in nodes of Ranvier. Dystroglycan, NrCam, pERM (not represented in the drawing), and Contactin remain in paranodes of dKO nerves. (TIF) [file pbio.1002258.s019.tif]
